# Geographical analysis of evaluated chronic disease programs for Aboriginal and Torres Strait Islander people in the Australian primary health care setting: a systematic scoping review

**DOI:** 10.1186/s12889-019-7463-0

**Published:** 2019-08-14

**Authors:** Hannah Beks, Marley J. Binder, Constance Kourbelis, Geraldine Ewing, James Charles, Yin Paradies, Robyn A. Clark, Vincent L. Versace

**Affiliations:** 1 Deakin Rural Health, Deakin University, School of Medicine, Geelong, Victoria Australia; 2 College of Nursing and Health Sciences, Flinders University, GPO Box 2100, Adelaide, South Australia Australia; 3 Institute of Koorie Education, Deakin University, Geelong, Victoria Australia; 4 School of Medicine, Deakin University, Geelong, Victoria Australia; 5 Faculty of Arts and Education, Deakin University, Burwood, Victoria Australia; 6 National Centre for Farmer Health, Western District Health Service, Hamilton, Victoria Australia

**Keywords:** Aboriginal and Torres Strait Islander people, Oceanic ancestry group, Chronic disease, Primary health care, Health services, indigenous, Program evaluation, Bioethics

## Abstract

**Background:**

Targeted chronic disease programs are vital to improving health outcomes for Indigenous people globally. In Australia it is not known where evaluated chronic disease programs for Aboriginal and Torres Strait Islander people have been implemented. This scoping review geographically examines where evaluated chronic disease programs for Aboriginal people have been implemented in the Australian primary health care setting. Secondary objectives include scoping programs for evidence of partnerships with Aboriginal organisations, and use of ethical protocols. By doing so, geographical gaps in the literature and variations in ethical approaches to conducting program evaluations are highlighted.

**Methods:**

The objectives, inclusion criteria and methods for this scoping review were specified in advance and documented in a published protocol. This scoping review was undertaken in accordance with the Joanna Briggs Institute (JBI) scoping review methodology. The search included 11 academic databases, clinical trial registries, and the grey literature.

**Results:**

The search resulted in 6894 citations, with 241 retrieved from the grey literature and targeted organisation websites. Title, abstract, and full-text screening was conducted by two independent reviewers, with 314 citations undergoing full review. Of these, 74 citations evaluating 50 programs met the inclusion criteria. Of the programs included in the geographical analysis (*n* = 40), 32.1% were implemented in Major Cities and 29.6% in Very Remote areas of Australia. A smaller proportion of programs were delivered in Inner Regional (12.3%), Outer Regional (18.5%) and Remote areas (7.4%) of Australia. Overall, 90% (*n* = 45) of the included programs collaborated with an Aboriginal organisation in the implementation and/or evaluation of the program. Variation in the use of ethical guidelines and protocols in the evaluation process was evident.

**Conclusions:**

A greater focus on the evaluation of chronic disease programs for Aboriginal people residing in Inner and Outer Regional areas, and Remote areas of Australia is required. Across all geographical areas further efforts should be made to conduct evaluations in partnership with Aboriginal communities residing in the geographical region of program implementation. The need for more scientifically and ethically rigorous approaches to Aboriginal health program evaluations is evident.

**Electronic supplementary material:**

The online version of this article (10.1186/s12889-019-7463-0) contains supplementary material, which is available to authorized users.

## Background

It is well established that Indigenous people experience poorer health outcomes than non-Indigenous people globally [1]. Australian Aboriginal and Torres Strait Islander people, like other Indigenous populations in Canada, New Zealand and the United States, endure ongoing health inequities such as a high burden of chronic disease and difficulty accessing culturally safe health care [2, 3]. Chronic diseases with strong environmental and behavioural etiology, such as cardiovascular disease and Type Two Diabetes Mellitus (T2DM), contribute to approximately 80% of the mortality gap between Australian Aboriginal and non-Aboriginal people between 35 and 74 years of age [4]. Aboriginal people residing in more geographically remote areas experience further disadvantage and a higher burden of chronic disease [5]. For example, the proportion of Aboriginal people with Diabetes Mellitus in Very Remote areas of Australia is approximately twice that of Aboriginal people in Major Cities [6]. The lack of affordable fresh fruit and vegetables in these areas is one contributing factor [7].

Targeted chronic disease prevention and management programs delivered in the primary health care setting are imperative to alleviating the burden of disease and improving health outcomes for Indigenous people [3, 8]. In Australia, little progress has been made in improving health outcomes, the distribution of chronic disease, and risk factors for developing disease for Aboriginal people [2, 9]. This is despite numerous funded Aboriginal chronic disease programs implemented at a national level (e.g., Aboriginal Chronic Disease Package, 2008) and initiatives under the ‘Closing the Gap’ policy [2, 9]. The ineffectiveness of health programs has been attributed to multiple factors, including short government funding cycles, a lack of community ownership and consultation, and a ‘one size fits all’ approach to program design and implementation [10, 11]. Furthermore, only a small proportion of Aboriginal health programs have been evaluated (8%), with only 6% of program evaluations applying rigorous evaluation methodologies to measure program effectiveness [12, 13]. The paucity of Aboriginal health program evaluations has resulted in little opportunity to improve or modify existing programs in response to program outcomes, contributing to the cycle of program ineffectiveness.

The need for Aboriginal community-driven programs and governance of primary health care services, as supported by the international right to self-determination for Indigenous people, is becoming increasingly recognised as a key strategy to alleviating the burden of chronic disease [14–16]. Strong evidence supports the role of Aboriginal Community-Controlled Health Organisations (ACCHOs) and other Aboriginal organisations in improving the accessibility, appropriateness and effectiveness of primary health care services through the provision of culturally appropriate care which respects the cultural values and beliefs of Aboriginal people [8]. Therefore, the involvement of ACCHOs and other Aboriginal organisations in the design and implementation of chronic disease programs is imperative [15, 17–19]. Moreover, a community-based approach to program design, implementation, evaluation, sustainability and transferability acknowledges the diversity of Aboriginal culture, language and customs [10]. This ensures that chronic disease programs are tailored to local needs and evaluated in partnership with community, recognises the strengths and resilience of Aboriginal people, and empowers Aboriginal communities to promote their own health and wellbeing [20].

Although there has been a rhetorical shift from government initiated health programs to community-developed health programs for Aboriginal people in Australia [21], it is not known whether the distribution of chronic disease programs has been proportionate to the population distribution of Aboriginal people, or to the burden of chronic disease. Furthermore, it is not known geographically where evaluated chronic disease programs for Aboriginal people in the primary health care setting have been implemented. Therefore, the purpose of this scoping review was to identify where evaluated chronic disease programs have been implemented in the primary health care setting [22]. Specifically, this review sought to determine whether this distribution was proportionate to the Aboriginal population distribution, and burden of disease across all geographical areas of Australia and by doing so, highlight geographical gaps in the literature to identify priority areas for the implementation of chronic disease programs. Secondary objectives included scoping for evidence of partnerships with ACCHOs and other Aboriginal organisations, in addition to the use of ethical guidelines or protocols in the reporting of programs.

## Methods

This study provides a systematic scoping review geographically examining the distribution of evaluated chronic disease prevention and management programs implemented for Australian Aboriginal people in the primary health care setting which includes community-health settings, general practice clinics and ACCHOs [22]. This review was undertaken in accordance with the methodology for conducting scoping reviews as outlined in the Joanna Briggs Institute Reviewers’ Manual 2017: Methodology for JBI Scoping Reviews [23]. Search terms were designed in a PCC (Population, Concept, Context) format by the research team and in collaboration with a health librarian. The premise and methods for this review have been published elsewhere in greater detail [22]. The Preferred Reporting Items for Systematic Reviews and Meta-analyses (PRISMA) guidelines were adhered to in the reporting of this review (Additional file 1).

### Search strategy

A preliminary search was conducted in MEDLINE and CINAHL using keywords to develop a tailored search strategy for each information source. A combination of Boolean operators, truncations and Medical Subject Headings (MeSH) were used to develop database search strategies (Additional file 2). The following databases were searched: Ovid MEDLINE, CINAHL (EBSCOhost), Scopus, Embase (Elsevier), Cochrane Database of Systematic Reviews, ISI Web of Science, SocINDEX (EBSCO-host), Sociological Abstracts (ProQuest), PubMed Central and PsycINFO (OVID).

Keywords were used to search the following information sources for unpublished studies, grey literature, and trials in order to avoid publication bias: Lowitja Institute, Indigenous Healthinfonet, National Aboriginal Community Controlled Health Organisation (NACCHO), Department of Health (Australian Government), informIT, Google, Cochrane Central Trials Register of Controlled Trials, ANZ Clinical Trials Registry, ClinicalTrials.gov, WHO International Clinical trial Registry Platform (ICTRP), Primary Health Care Research and Information Service (PHCRIS), ProQuest Dissertations and Theses Global, Trove and OAIster.

### Inclusion criteria and exclusion criteria

This review considered literature based on the following criteria (Table 1).

**Table 1:** Inclusion and exclusion criteria

	Inclusion criteria	Exclusion criteria
Population	Involved Aboriginal and/or Torres Strait Islander adults 18 years of age and above who had participated in a chronic disease program Program evaluation involved over 50% Aboriginal participation or stratified analysis for Aboriginal people	Involved children or young people less than 18 years of age
Concept	Evaluated chronic disease programs involving disease prevention and/or management activities for, but not limited to, chronic diseases such as cardiovascular disease, chronic obstructive pulmonary disease, diabetes, asthma, arthritis, chronic pain, cancer, mental health conditions, chronic kidney disease, liver disease or tooth decay and/or risk factors for developing chronic diseases, such as an unhealthy weight, exceeding alcohol drinking guidelines, smoking, poor diet or physical inactivity.	Program not evaluated
Context	Program evaluated in the Australian primary health care context (e.g. ACCHOs, general practice clinics and community-health clinics)	Programs evaluated in inpatient hospital facilities and sub-acute rehabilitation facilities Outcomes not published in English

No restrictions were placed on the quality of evaluation or study design. As stated in the scoping review protocol, programs evaluated by any party to any level were included [22]. Literature published from 1 January 2006, was included in order to capture programs published since the launch of the ‘Closing the Gap’ campaign, which resulted in a greater focus on addressing health inequities experienced by Aboriginal people in Australia [9].

For consistency, the term ‘Aboriginal’ has been used throughout this review to refer to both Aboriginal and/or Torres Strait Islander people in Australia. This is due to brevity, and no disrespect is intended to any individual or group. The term ‘Indigenous’ has been reserved for the global context.

### Study selection and data extraction

Searches for published and unpublished literature were conducted by a health librarian. Titles and abstracts retrieved from the search were screened independently by two reviewers (HB and MJB). Conflicts were resolved through discussion with a third reviewer (CK). Full text review and data extraction was then conducted independently by two reviewers (HB and MJB) on selected articles. Reasons for exclusion were provided for articles that did not meet the review criteria. The reference lists of citations requiring full text review were also screened for additional citations in order to ensure that all possible literature was included.

Extracted data were categorised under the following headings: author, year of publication, year of program implementation, location of program implementation/evaluation, evaluation methods, involvement of an ACCHO/other Aboriginal organisation and reference to Australian National Health and Medical Research Council’s (NHMRC) ‘Values and Ethics: Guidelines for Ethical Conduct in Aboriginal and Torres Strait Islander Health Research’ guideline, and other ethical protocols [24, 25]. Geographical coordinates were then assigned to included programs based on the extracted data. Where specific implementation sites were not stated, the approximate location(s) were geocoded and coordinates extracted. If this information was unavailable, the corresponding author was contacted. If studies did not specify where the program was evaluated, the institution listed for the first author was used as a proxy for place of evaluation. This assumed that first authorship implied a lead role in the evaluation.

Coordinates were then exported to ArcGIS® ArcMap™ and overlayed with the Remoteness Areas of Australia for analysis [26]. To define remoteness, the Australian Statistical Geography Standard (ASGS) was applied, which is a categorisation of the Accessibility/Remoteness Index of Australia (ARIA+) [27]. Areas are classified as: i) Major Cities of Australia, ii) Inner Regional Australia, iii) Outer Regional Australia, iv) Remote Australia, and v) Very Remote Australia. Euclidean distance between the implementation site and evaluation were also extracted in ArcGIS® ArcMap™ [26]. Summary statistics were produced to examine the distance between implementation site(s) and place of evaluation. Locations of implementation and evaluation were stratified by Remoteness Area and cross-tabulated.

The extracted data, synthesis of findings and review outcomes, were critically reviewed for culturally appropriateness by two Aboriginal researchers, as stated in the review protocol [22].

## Results

**Fig. 1:**
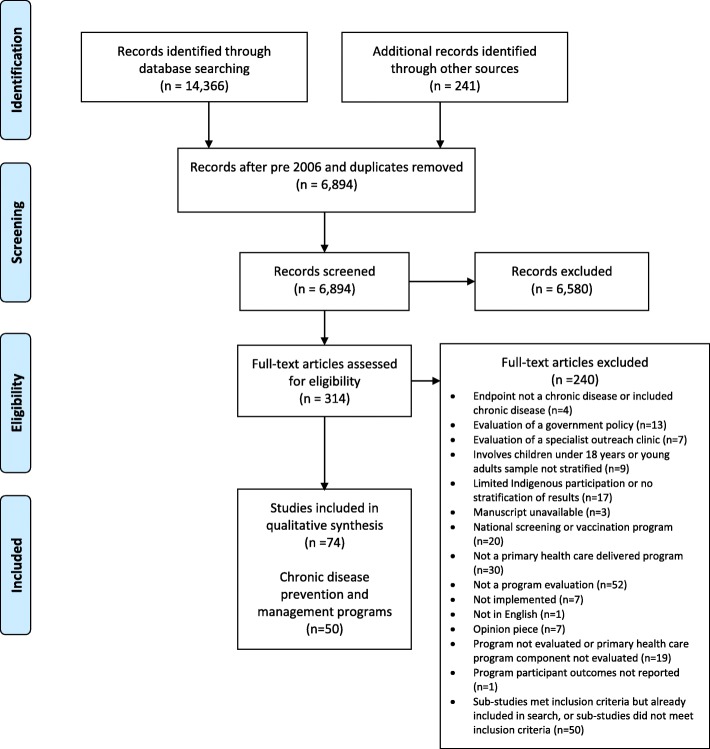
PRISMA diagram of the systematic review process for this review

Database searches yielded 14,366 citations. An additional 241 citations were retrieved from a search of the grey literature and targeted organisation websites. A total of 6894 title and abstracts were screened, with duplicates removed. The full texts of 314 citations were screened for relevance to the review criteria, identifying 74 pertinent records evaluating 50 chronic disease prevention and management programs (Fig. 1 – PRISMA Flow Diagram). One of these records included reference to three evaluated programs [28].

Reasons for excluding records were provided (Additional file 3). The most frequent reason provided for exclusion was that the record was ‘not a program evaluation’ (*n* = 52), followed by ‘Sub-studies met inclusion criteria but already included in search, or sub-studies did not meet inclusion criteria’ (*n* = 50) and ‘not a primary health care delivered program’ (*n* = 30). Excluded records included 20 records which focused on evaluating national screening and vaccination programs. These were excluded as findings were based on national or state-wide aggregate data which would have been difficult to include in the geographical analysis.

### Finding 1: heterogeneity of included programs

Citations meeting the review criteria (*n* = 74) included evaluated programs (*n* = 50) that addressed multiple chronic diseases (*n* = 16), a specific chronic disease (cardiovascular disease n = 5, diabetes mellitus *n* = 6, chronic kidney disease *n* = 3, liver disease *n* = 2, mental illness *n* = 4, oral disease n = 2 and polycystic ovarian syndrome n = 1) or risk factors for developing chronic disease (drug and alcohol misuse n = 2, poor nutrition and physical inactivity n = 3 and smoking n = 6) (Table 2). Of the included programs, 74% (*n* = 37) aimed to prevent and/or manage chronic disease using disease-specific screening, early intervention or treatment strategies, with the remaining programs (*n* = 13) applying general health promotion approaches to disease prevention, such as empowering participants to implement activities to improve their health.

**Table 2:** Characteristics of included program evaluations

Program name	Citation	Years of program	Type of program	Targeted chronic disease(s)/risk factor(s)	Evaluation study design	Aboriginal participant sample size	Evaluation outcome measures
Cooking Classes for Diabetes Program	Aboriginal Health & Medical Research Council 2009 [28] Abbott, Davison, Moore & Rubinstein 2010 [29] Abbott, Davison, Moore & Rubinstein 2012 [30]	2002–2007	Health promotion	Diabetes, Poor nutrition	Qualitative - post program semi-structured interviews	73 program participants, 23 interview participants (4 m, 19 f)	Participant experience
Health Lifestyle and Weight Management Program	Aboriginal Health & Medical Research Council 2009 [28]	2005–2008	Health promotion and chronic disease prevention	Poor nutrition, physical inactivity	Mixed methods -pre, interim and post program quantitative and qualitative measures	Not reported	Clinical measures: BMI, height, weight, blood pressure, blood sugar level, waist, chest and hip ratio Participant experience
Healthy Food Awareness Program	Aboriginal Health & Medical Research Council 2009 [28]	2008	Chronic disease prevention and management	Poor nutrition, physical inactivity, smoking, obesity, renal disease, diabetes and other chronic diseases	Not reported	Not reported	Not reported
‘No More Dhonga’ Short Course	Adams et al. 2006 [31]	2004	Health promotion and chronic disease prevention	Smoking	Mixed methods-interim and post program measures	32 participants	Stakeholder feedback Course attendance and smoking quit rate
Home-Based, Outreach case Management of chronic disease Exploratory (HOME) Study program	Askew et al. 2016 [32]	Not reported	Chronic disease management	Diabetes type 2, cardiovascular disease, respiratory disease, kidney disease	Mixed methods-post program semi-structured interviews, pre, interim and post program quantitative measures	41 participants, data collected from 37 participants (32 m, 68% f)	Feasibility, acceptability and appropriateness of model
Renal Treatment Program	Bailie et al. 2006 [33]	1995–1999	Chronic disease management	End state renal disease	Quantitative-interrupted time series of pre/post quantitative measures	266 participants, data collected from 98 participants	Clinical measure: blood pressure
Moorditj Djena program	Ballestas et al. 2014 [34]	2011-ongoing	Chronic disease management	Diabetes type 2, peripheral arterial disease, peripheral neuropathy	Mixed methods- interim program focus groups, interviews and review of quantitative data	Data collected from 702 participants (majority Aboriginal – not specified) Participation not reported for qualitative data	Program delivery, quality of implementation and organizational context
Nurse-led practitioner project for chronic kidney disease	Barrett et al. 2015 [35]	2012-ongoing	Chronic disease management	Chronic kidney disease	Quantitative-clinical audit	187 participants	Rates of detection and improvement in chronic disease management
Flinders self-management model (CCSM)	Battersby et al. 2008 [36]	2001–2002	Chronic disease management	Diabetes	Mixed methods-pilot study with pre, interim and post quantitative data, post program focus group	60 participants (28 m, 32 f)	Program acceptability and clinical outcomes (HbA1c, Diabetes Assessment Form, SF-12)
Polycystic Ovarian Syndrome clinic program	Boyle et al. 2017 [37]	2012–2013	Chronic disease management	Polycystic Ovarian Syndrome (PCOS)	Mixed methods-post implementation evaluation using clinical audit, semi-structured interviews and focus groups	Clinical audit involved 36 f participants, interviews with 8 clinicians and focus group with 8 f participants	Process evaluation of program fidelity, barriers and enablers and whether the program met community needs
Diabetic retinopathy screening program	Brazionis et al. 2018 [38]	2014–2016	Chronic disease prevention and management	Diabetes	Quantitative- cross-sectional study design	301 participants (33% m, 67% f)	Clinical effectiveness: diabetic retinopathy prevalence rates and severity compared to other screening programs
Primary Health Care Outreach program of Aboriginal Health Checks	Burgess et al. 2011 [39]	2005	Chronic disease management	Cardiovascular disease and other chronic diseases	Quantitative- interrupted time series study with pre/post measures	64 participants (43 m, 21 f)	Clinical measures (absolute cardiovascular risk, blood pressure, BMI), follow up appointments and outcomes
12 week exercise and nutrition program	Canuto et al. 2012 [40] Canuto 2013 [41] Canuto et al. 2013 [42]	2010–2011	Health promotion	Poor nutrition, physical inactivity	Mixed methods-pragmatic randomised trial with mixed methods process evaluation	100 f participants at baseline, 41 lost to follow up. Not reported how many participated in interviews	Program effectiveness on waist circumference, weigh and biomedical metabolic markers Factors influencing program attendance
Healthy Lifestyle Programme (HELP)	Chan et al. 2007 [43]	Not reported	Chronic disease management	Diabetes, cardiovascular risk factors	Quantitative- pre and post study	101 participants	Effectiveness of a lifestyle intervention on clinical measures
Cardiac failure education program	Clark et al. 2014 [44] Clark et al. 2015 [45]	Not reported	Chronic disease management	Cardiovascular disease	Mixed methods-pilot study with pre and post data	5 participants (3 m, 2 f)	Feasibility and acceptability of resource
Drug and alcohol screening intervention	Clifford et al. 2013 [46]	Not reported	Chronic disease prevention	Drug and alcohol misuse	Quantitative- pre and post study	314 participants	Proportion of clients with alcohol screening
Health literacy intervention	Crengle et al. 2017 [47]	2013	Chronic disease management	Cardiovascular disease	Quantitative-multi-site pre and post study	171 participants, 11 lost to follow up	Effect of intervention on medication knowledge
Grog mob	D’Abbs et al. 2013 [48]	2008–2009	Chronic disease prevention	Risky alcohol behaviour	Mixed methods-descriptive analysis of post program data	49 participants	Examine whether program met its objectives, document implementation processes and gauge the impact on client outcomes
Cardiac and pulmonary secondary prevention program	Davey et al. 2014 [49]	2011–2013	Chronic disease prevention and management	Cardiovascular and pulmonary disease	Mixed methods-pre and post study	92 participants (36 m, 56 f), qualitative feedback from 51 participants	Program uptake and effectiveness
Smoking cessation program	DiGiacomo et al. 2007 [50]	2005–2006	Chronic disease prevention	Smoking	Quantitative- case review	37 participants (10 m, 27 f)	Screening rates and quit attempts
‘Heart health’ program cardiac secondary prevention	Dimer et al. 2010 [51] Dimer et al. 2012 [52] Dimer et al. 2013 [53] Maiorana et al. 2012 [54] Maiorana et al. 2015 [55]	2009–2010	Chronic disease prevention and management	Cardiovascular disease	Mixed methods-pre and post data, interviews, yarning sessions and questionnaires	98 participants (35 m, 63 f)	Uptake and effectiveness of program on lifestyle and cardiovascular risk factors
Intensive quit smoking intervention	Eades et al. 2012 [56]	2005–2009	Health promotion and chronic disease prevention	Smoking	Quantitative-randomised controlled trial	263 f participants	Effectiveness of intervention on smoking rates
Give up the smokes program	Gould, McGechan & Zwan 2010 [57]	2007–2008	Health promotion and chronic disease prevention	Smoking	Quantitative- pre and post study	10 participants	Cultural appropriateness of program
Diabetes Management and Care program	Gracey et al. 2006 [58]	2002	Chronic disease prevention and management	Diabetes, poor nutrition, physical inactivity	Quantitative- pre and post study	418 participants (181 m, 237 f)	Impact of program on clinical measures
Koorie Men’s health day	Isaacs & Lampitt 2014 [59]	Not reported	Health promotion and chronic disease prevention	Mental illness	Mixed methods-descriptive study	20 m participants (data available for 17)	Model outcomes
Oral health literacy program	Ju et al. 2017 [60]	Not reported	Health promotion	Oral health	Quantitative-randomised controlled trial	400 participants at baseline, 106 lost to follow up	Oral health literacy
Oral health periodontal program	Kapellas et al. 2013 [61] Kapellas et al. 2014a [62] Kapellas et al. 2014b [63] Kapellas et al. 2017 [64]	2010–2012	Chronic disease prevention and management	Oral health	Quantitative-randomised controlled trial	273 participants, follow up data available for 169	Improvements in clinical outcomes
Structured chronic disease care planning program	Kowanko et al. 2012 [65]	2008–2011	Chronic disease management	All chronic diseases	Mixed methods-Participatory Action Research framework	36 participants involved in longitudinal study, otherwise not reported	Impact of chronic disease self-management strategies on health outcomes
Nurse-led Chronic Kidney Disease program	Lawton et al. 2016 [66]	2007-ongoing	Chronic disease management	Chronic kidney disease	Quantitative-interrupted time series	Not reported	Improvement in rate of chronic kidney disease detection and clinical markers
Walk about Together Program (WAT)	Longstreet et al. 2008 [67]	2003–2005	Health promotion	Unhealthy weight, poor nutrition	Quantitative-pre and post study	100 participants (12% m, 88% f).	Nutrient intake of program participants
Be Our Ally Beat Smoking (BOABS) program	Marley et al. 2014a [68] Marley et al. 2014b [18]	2009–2012	Health promotion	Smoking	Mixed methods-randomised controlled trial with qualitative component	168 randomised, 19 lost to follow up	Efficacy of smoking cessation program at 12 months follow up
Getting better at chronic care program	McDermott et al. 2015 [69] Schmidt, Campbell & McDermott 2016 [70] Segal et al. 2016 [71]	2011–2013	Chronic disease management	Diabetes and other chronic diseases	Mixed methods-pragmatic cluster randomised controlled trial with qualitative component and economic analysis	213 participants randomised (38% m, 62% female), 24 lost to follow up, 21 interview participants	Program effectiveness in improving care of participants with diabetes Experience of health workers implementing program Program cost-effectiveness
Work it out program	Mills et al. 2017 [72]	2012–2014	Chronic disease prevention and management	Cardiovascular disease	Quantitative- quasi-experimental with pre and post data	85 participants	Impact on clinical outcomes at 12 weeks post implementation
Mental illness brief intervention program	Nagel & Thompson 2008 [73] Nagel et al. 2008 [74]	2004–2007	Chronic disease management	Mental illness	Mixed methods-randomised controlled trial with qualitative component	49 participants	Program effectiveness on clinical outcomes
Get Healthy Service program	Quinn et al. 2017 [75]	2009–2015	Health promotion	All chronic diseases	Mixed methods-pre and post study with qualitative component	30 participants interviewed (5 m, 25 f), quantitative data collection involved 1462 participants	Program reach and impact on lifestyle risk factors
Antiviral therapy Hepatitis C program	Read et al. 2017 [76]	2016-ongoing	Chronic disease prevention and management	Hepatitis C	Quantitative-observational cohort study	23 participants	Efficacy of program
Quality Assurance for Aboriginal & Torres Strait Islander Medical Services (QAAMS) program	Shephard 2006 [77] Shephard et al. 2017 [78] Spaeth, Shephard & Schatz 2014 [79]	1999-ongoing	Chronic disease management	Diabetes	Mixed methods-key stakeholder and client questionnaire with open questions, case studies, comparison of baseline and post implementation data, longitudinal quality assurance data, before and after study design	161 participants completed client questionnaire, 907 program participants	Program satisfaction Quality assurance and imprecision Clinical and operational efficiency
Point-of-Care in Aboriginal Hands	Shepherd et al. 2006 [80]	2001-ongoing	Chronic disease management	All chronic diseases	Mixed methods-interviews, comparison of baseline and post implementation data	Data collected from 626 participants	Community acceptability of program
Western Desert Kidney Health Screening program	Sinclair et al. 2016 [81]	2012	Chronic disease prevention and management	Chronic kidney disease, diabetes	Qualitative-interviews	26 participants (11 m, 15 f)	Community acceptability of program
COACH programme	Ski et al. 2017 [82]	Not reported	Chronic disease prevention and management	Cardiovascular disease	Quantitative-longitudinal outcomes in participants	Not reported	Program effectiveness in reducing cardiovascular risk
Diabetic retinopathy screening program	Spurling et al. 2010 [83]	2007–2009	Chronic disease management	Diabetes	Mixed methods-semi-structured interviews, descriptive analysis of demographic data and screening rates	132 participants (60 m, 72 f)	Program impact and accessibility
Indigenous adult health checks program	Spurling, Hayman & Cooney 2009 [84]	2007–2008	Chronic disease prevention and management	All chronic diseases	Quantitative- cross-sectional study	413 participants	Evaluate role of program
Shared medical appointment program	Stevens et al. 2016 [85]	Not reported	Chronic disease prevention and management	All chronic diseases	Mixed methods-post program questionnaires, interviews and field notes	14 m participants	Program acceptability and appropriateness
Community singing program	Sun & Buys 2012 [86] Sun & Buys 2013a [87] Sun & Buys 2013b [88] Sun & Buys 2013c [89] Sun & Buys 2013d [90] Sun & Buys 2013e [91] Sun & Buys 2013f [92] Sun & Buys 2016 [93]	2010–2012	Chronic disease management	Cardiovascular disease, diabetes, cancer, depression, psychosis	Mixed methods-pre and post study design with numerous outcome measures, questionnaires, focus group sessions	45 participants	Program effectiveness and impact
Home Medicines Review program	Swain 2016 [94] Swain & Barclay 2015 [95]	2001-ongoing	Chronic disease management	All chronic diseases	Mixed methods-focus group sessions with indigenous consumers, interviews with health workers, cross-sectional survey with pharmacists	102 participants	Usefulness of program for Indigenous people Facilitators and barriers to program uptake
‘Yaka Narali’ Tackling Indigenous Smoking program	Tane et al. 2016 [96]	2009-ongoing	Health promotion	Smoking	Qualitative-interviews	30 participants	Program effectiveness
Ngangkari Program	Togni 2017 [97]	Not reported	Chronic disease management	Mental illness, Social and Emotional Wellbeing	Qualitative-interviews and focus group sessions	18 participants	Developmental evaluation of program model
Deadly Liver Mob program	Treloar et al. 2018 [98]	2013-ongoing	Health promotion and chronic disease prevention	Hepatitis C	Mixed methods-pre and post study with qualitative component	Quantitative data collected from 710 participants, 19 participant interviews	Program acceptability
Music therapy program	Truasheim 2014 [99]	2012	Chronic disease management	All chronic diseases	Mixed methods-survey data and some clinical measures	13 participants (4 m, 9 f)	Examine cultural safety of program
Perinatal mental health program	Verrier et al. 2013 [100]	Not reported	Chronic disease prevention and management	Mental illness, Social and Emotional Wellbeing	Mixed methods-pre and post study with quantitative and qualitative data	Not reported	Program impact

The data collection methods of program evaluations varied, with over half of the program evaluations using a mixed methods approach (*n* = 26, 52%), followed by a quantitative only (*n* = 19, 38%) or qualitative only (*n* = 4, 8%) approach. The methods of evaluation were not reported for one program [28], however, a summary of outcomes were provided; hence the program was included in the review. Of the included programs, only seven were evaluated using a randomised controlled trial (RCT) study design, with only one study (an RCT) including an economic evaluation of program cost-effectiveness.

### Finding 2: geographical distribution of programs

Four of the included programs were excluded from the geographical analysis as programs were implemented state-wide or nationally (Home Medicines Review Program [94, 95], Get Healthy Service Program [75], QAAMS Program [77–79] and COACH Program [82]). Five of the included programs were also excluded from the geographical analysis as authors did not respond to the request for additional information. Geographical coordinates for program implementation sites were available for 41 of the included programs (82% of all included programs). However, one program was omitted from the analyses as the evaluation was undertaken outside of Australia as part of a multi-site program evaluation [47]. A total of 81 implementation sites for the 40 programs (80% of all included programs) with available locations were geo-coded and geographically analysed (Table 3).

**Table 3:** Geographical location of included programs^a^

Evaluation n (%)							
Implementation n (%)	Major Cities	Inner Regional	Outer Regional	Remote	Very Remote	Total	Aboriginal population (%)^b^
Major Cities	26 (32.1)					26 (32.1)	37.4
Inner Regional		10 (12.3)				10 (12.3)	23.7
Outer Regional	14 (17.3)		1 (1.2)			15 (18.5)	20.3
Remote	3 (3.7)		1 (1.2)	2 (2.5)		6 (7.4)	6.7
Very Remote	15 (18.5)		6 (7.4)	2 (2.5)	1 (1.2)	24 (29.6)	11.9
Total	58 (71.6)	10 (12.3)	8 (9.9)	4 (4.9)	1 (1.2)	81 (100)	100

^a^Excludes included programs implemented at a national or state level (Home Medicines Review Program [94, 95], Get Healthy Service Program [75], QAAMS Program [77–79] and COACH Program [82]), programs where geographical coordinates were not provided by authors (n = 5) and one program where the evaluation was undertaken overseas as part of a multi-site program evaluation [47]

^b^2016 Australian Aboriginal population distribution across Remoteness Areas [101]

Of the included programs in the geographical analysis (*n* = 40), 32.1% were implemented for Aboriginal people residing in Major Cities of Australia and 29.6% for Aboriginal people residing in Very Remote Australia. The remaining programs were implemented for Aboriginal people residing in the intermediate remoteness areas of Inner Regional Australia, Outer Regional Australia, and Remote Australia (12.3, 18.5 and 7.4% respectively).

**Fig. 2:**
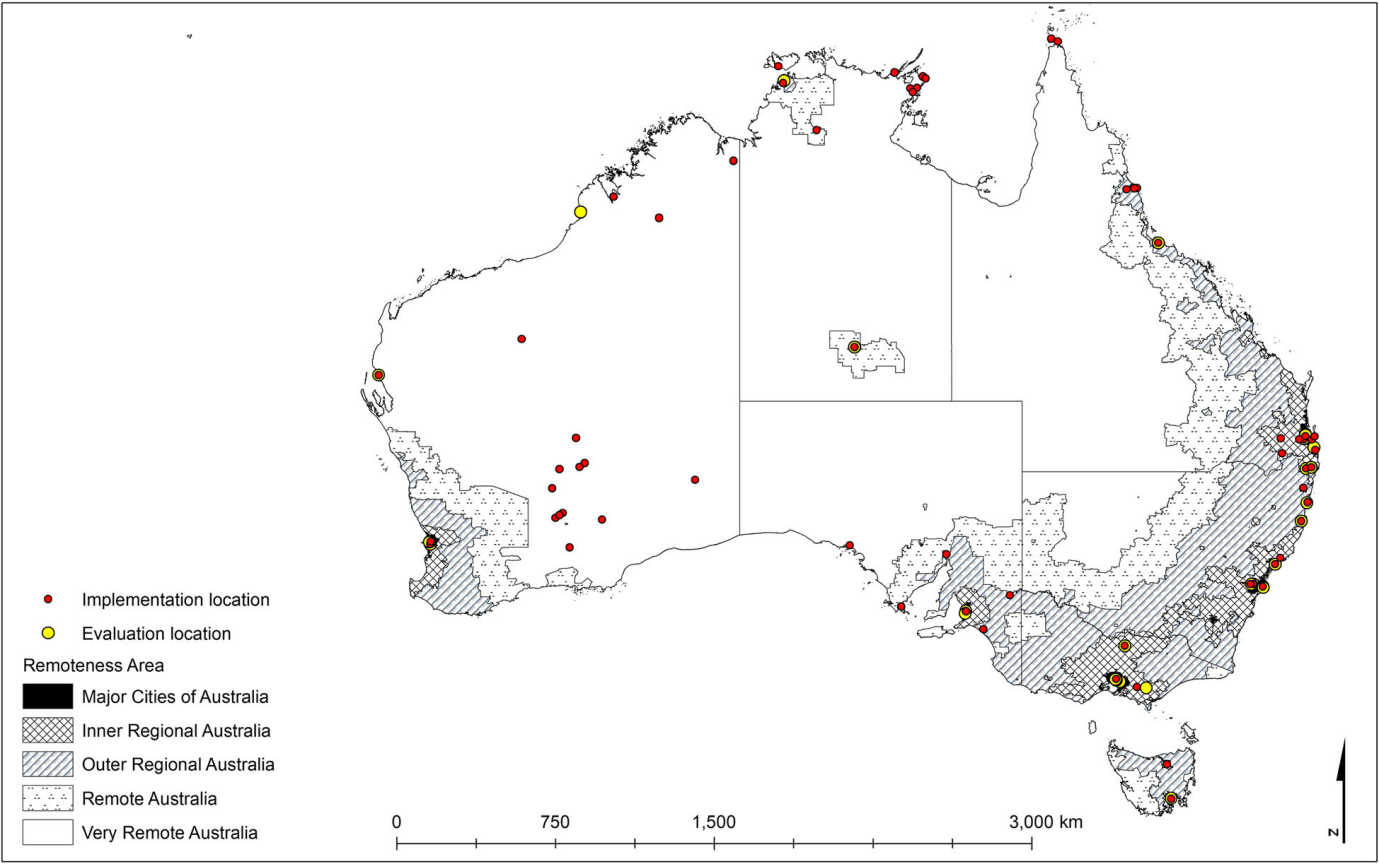
Locations of the implementation and evaluation of programs in relation to the Remoteness Areas of Australia [27]

The location of program evaluation was reported for 25 of the programs included in the geographical analysis. First author affiliation was used as a proxy for the location of program evaluation for the remaining 15 programs. Evaluation activity was predominately undertaken in Major Cities of Australia (71.6%), with the remaining studies declining in order of remoteness. Of the identifiable implementation locations (*n* = 81), 18 (22%) of these also had an evaluation undertaken on site. For studies with the site(s) of implementation and evaluation available, the mean distance between implementation and evaluation was 660 km (95% CI 470–850; maximum 3041; median 223). A visual representation of the distribution of included programs is provided (Fig. 2).

The sample size of programs retrieved for highly prevalent chronic diseases, such as cardiovascular disease and T2DM, was deemed insufficient to geographically analyse whether the distribution of evaluated programs were proportionate to the burden of chronic disease across all Remoteness areas.

### Finding 3: ethical approaches to program evaluation

Of the 50 programs included in the review, 39 (78%) reported on the involvement of an ACCHO in the implementation or evaluation process (Additional file 4). Of the included programs that did not report on the involvement of an ACCHO, six of these referred to the involvement of another Aboriginal organisation (12%). Overall, 90% (*n* = 45) of the included programs collaborated with an Aboriginal organisation in the implementation and/or evaluation of the program.

When examining the affiliation of the first author of the included citations (*n* = 74), 74% (*n* = 55) were associated with a university or research institution, 9.5% (n = 7) with an ACCHO or other Aboriginal organisation, 9.5% (n = 7) with a non-Aboriginal health service or Non-Government Organisation (NGO) and 7% (n = 5) with both a university or research institution and an ACCHO.

Of the 74 citations retrieved, seven explicitly referred to the NHMRC’s ‘Values and Ethics: Guidelines for Ethical Conduct in Aboriginal and Torres Strait Islander Health Research’ as a guideline underpinning the evaluation design and conduct [24]. However, 70% of citations (*n* = 52), particularly those published in a peer-reviewed journal, included a formal statement of ethical review and approval by a Human Research Ethics Committee (HREC) affiliated with a research institution or university. Only 23 citations (31%) discussed the use of other ethical protocols or a community-based ethical review process. These citations varied broadly in their descriptions of adhering to local cultural guidelines or consulting with an appointed Aboriginal advisory group. For example, Askew et al. (2016) [32] described the formation of a research advisory group consisting of both Aboriginal community members and experienced researchers who provided research governance and oversight, whereas Treloar et al. (2018) [98] described consulting with an Aboriginal advisory group in the program development phase rather than the evaluation process. Consideration of cultural sensitivities was also discussed broadly in some papers, including processes undertaken to build rapport with collaborating Aboriginal communities prior to the conduct of an evaluation [59, 72] and the receipt of cultural guidance or support from a steering group of Aboriginal people or Elders [40, 83].

## Discussion

This review highlights the paucity of Aboriginal chronic disease program evaluations conducted in the primary health care setting across all geographical regions of Australia. Previous studies have acknowledged that only a small proportion of Aboriginal health programs have been subject to an evaluation process [12, 13]. Therefore, the included programs in this review are not representative of all chronic disease programs implemented for Aboriginal people across Australia. Of those included, the majority targeted highly prevalent chronic diseases, such as cardiovascular disease and T2DM, or risk factors for developing chronic disease, such as smoking and physical inactivity [2]. Due to the heterogeneity of programs across all geographical regions and small sample size, the review was unable to ascertain whether the spread of programs was proportionate to the distribution of chronic disease across all Remoteness Areas of Australia. For example, it was difficult to conclude whether a greater focus on the management of T2DM for Aboriginal people residing in Very Remote areas is required, where the prevalence of T2DM is approximately twice of Aboriginal people residing in Major Cities [6]. However, the small proportion of evaluated social and emotional wellbeing (SEWB) programs (e.g., mental health programs) was noted across all geographical regions, supporting the need for tailored early intervention and screening SEWB programs for Aboriginal people [19]. Internationally, tailored programs for mental health prevention have been deemed particularly important for Indigenous people, particularly those including an exploration of cultural identity [102, 103].

Overall, Major Cities and Very Remote areas of Australia displayed similar levels of chronic disease program implementation activity, with less activity noted for Inner and Outer Regional (IOR) areas and Remote areas of Australia. A greater focus on chronic disease programs for Very Remote Aboriginal people when compared to IOR or Remote Aboriginal people could be informed by national data which indicates that the burden of chronic disease in Aboriginal people increases with Remoteness [6]. However, less Aboriginal people reside in Very Remote areas when compared to IOR areas (11.9% compared to 23.7 and 20.3% respectively), suggesting there is a need for the evaluation of chronic disease programs for Aboriginal people residing in IOR areas [101]. Across all geographical areas in Australia, it is anticipated that the demand for chronic disease prevention programs will increase over time, due to a higher Aboriginal population growth rate when compared to non-Aboriginal populations as indicated by 2017 national data (2.26 babies per Aboriginal woman compared to 1.75 babies per non-Aboriginal woman) [104, 105]. The demand for chronic disease programs may also increase for Indigenous people in other countries (e.g., Canada) experiencing similar population growth (2.2 babies per Aboriginal woman compared to 1.6 babies per non-Aboriginal woman in Canada) [106].

When considering evaluation activity, higher levels of evaluation were noted for Major Cities (71.6%) when compared to Very Remote areas (1.2%). This is despite the fact that national data indicates that less Aboriginal people reside in Major Cities compared to the total Australian population (37% compared to 73% respectively) [101]. Further to this, the proportion of Aboriginal people is higher in all other Remoteness Areas of Australia, relative to the total Australian population [101]. This finding suggests there is a need for more Aboriginal community-led research as supported by the broader literature [15, 19, 20]. However, caution should be applied in interpreting these findings as first author affiliation was used as a proxy for the location of program evaluation for 15 of the 40 programs included in the geographical analysis. The rationale for this assumption was that first authorship implied a lead role in the evaluation.

When examining first author affiliation for all included citations (*n* = 74), 74% (*n* = 55) of citations were associated with a university or research institution, with only 9.5% (n = 7) citations associated with an ACCHO or other Aboriginal organisation. A previous review of Aboriginal health programs in Australia also found that the majority of program evaluations (72%) were led by a research institution or university rather than an Aboriginal community organisation [107]. However, first author affiliation with a research institution or university does not necessarily mean that the evaluation did not have significant Aboriginal community input; particularly as 90% (*n* = 45) of included programs provided details of collaborating with an ACCHO or other Aboriginal organisation in the development or evaluation of the program. Strong support for the appropriateness of ACCHOs as a collaborating organisation for activities involving Aboriginal people is found in the literature [108]. Generally, ACCHOs are geographically accessible to Aboriginal people and valued for the provision of culturally safe primary health care [8, 109, 110].

Although the majority of programs partnered with an ACCHO or Aboriginal organisation, it is difficult to ascertain for all programs, the degree of community ownership and involvement in the evaluation process. This includes steps taken by evaluators to ensure the evaluation process was ethically and culturally appropriate for Aboriginal people [20]. As reporting the formal ethical review of a research project is a standard requirement for publication in a peer-reviewed journal, both nationally and internationally, it is not surprising that the majority of citations (70%, *n* = 52) provided a statement of formal review by an appointed committee (e.g. HREC). However, only 31% of the included citations (*n* = 23) provided some evidence of actions taken to adhere to Aboriginal community-based ethical protocols, or engagement with an Aboriginal advisory group in the design of the program or conduct of the evaluation. Indeed, a statement of formal ethical review does not provide sufficient detail describing how Aboriginal people were consulted and included in the evaluation process. Other Aboriginal program evaluation frameworks and models of Aboriginal health research should also be consulted, which are valuable in informing approaches to conducting program evaluations in partnership with Aboriginal people [20, 25, 111, 112]. Program evaluations of Aboriginal programs excluding partnerships, often lack relevance and integrity, and fail to translate to outcomes for Aboriginal people [12, 113].

### Limitations

It is important to acknowledge the limitations of this review. The selection criteria of the review influenced the geographical spread of studies retrieved. National screening and vaccination programs were excluded as program evaluations used national aggregate data. Geographical findings may also have been impacted by the exclusion of other programs which met the review criteria, but were excluded from the geographical analysis due to state-wide or national program implementation (Home Medicines Review Program [94, 95], Get Healthy Service Program [75], QAAMS Program [77–79] and COACH Program [82]). Furthermore, authors of five programs did not respond with additional information regarding the geographical program implementation locations which may also have influenced the analysis.

It is not known what proportion of evaluated chronic disease programs or implemented chronic disease programs have been included; a limitation cited by a similar review [107]. It is also possible that evaluated programs targeting more distal risk factors for chronic disease may have been overlooked. The availability of evaluation reports may also have influenced the types of citations retrieved. A recent investigation into the evaluation of health programs implemented for Aboriginal people in Australia found that only 33% of evaluation reports were available [20]. Further to this, it is acknowledged that a substantial amount of literature pertaining to Australian Aboriginal people is published in the grey literature [114]. Although the authors have made every effort to conduct a thorough search of the grey literature, it is possible some evaluation reports may not have been captured in this scoping review.

### Recommendations

A greater focus is required on evaluating chronic disease prevention and management programs for Aboriginal people across all geographical areas, particularly for Aboriginal people residing in Inner and Outer Regional areas of Australia. In addition, there is a need to focus on evaluating Social and Emotional Wellbeing (SEWB) programs developed for Aboriginal people. Programs should be implemented and evaluated in collaboration with partnering ACCHOs or other Aboriginal organisations, with an emphasis on accountability, sustainability, capacity-building, ownership and Aboriginal strengths. This includes equipping Aboriginal organizations with skills in conducting program evaluations. Evaluation reporting should be transparent in describing ethical approaches to conducting the program evaluation in partnership with Aboriginal communities. Furthermore, an evaluation process should be integrated into the design of Aboriginal health programs. Evaluation outcomes should be publicly available, ideally through the peer-reviewed literature, in order to build the evidence around the effectiveness of chronic disease programs for Indigenous peoples globally.

## Conclusions

A greater focus on the implementation and evaluation of chronic disease prevention and management programs for Aboriginal people in Australia is required, particularly for Aboriginal people residing in Inner and Outer Regional Areas of Australia. There is also a need to conduct evaluations of Social and Emotional Wellbeing (SEWB) programs across all geographical regions. This review highlights the need for more ethically rigorous approaches to Aboriginal health program evaluations which engage Aboriginal people in all stages of program design, implementation, evaluation and sustainability.

## Additional files

Additional file 1: PRISMA 2009 Checklist. This file contains the PRISMA checklist and co-relating page numbers to the items for reporting. (DOCX 25 kb)

Additional file 2: Electronic search results and terms. This file contains a table of search results and terms used to retrieve studies from databases (DOCX 43 kb)

Additional file 3: Excluded Studies. This file contains a table of excluded studies and reasons for exclusion. (DOCX 68 kb)

Additional file 4: Data extraction: evidence of partnerships and reference to ethical guidelines. This file contains a table of data extracted in relation to the secondary objectives of this review; scoping evidence of partnerships with Indigenous organizations and ethical approaches to undertaking a program evaluation. (DOCX 95 kb)

## Data Availability

Not applicable.

## Abbreviations

ACCHO: Aboriginal Community-Controlled Health Organisation
BMI: Body Mass Index
HbA1C: Glycated Haemoglobin
HREC: Human Research Ethics Committee
IOR: Inner and Outer Regional
NGO: Non-Government Organisation
NHMRC: National Health and Medical Research Council
QAAMS: Quality Assurance for Aboriginal & Torres Strait Islander Medical Services
RCTs: Randomised Controlled Trials
SEWB: Social and Emotional Wellbeing
T2DM: Type Two Diabetes Mellitus
