# Supplementary material for: Geographical analysis of evaluated chronic disease programs for Aboriginal and Torres Strait Islander people in the Australian primary health care setting: a systematic scoping review
Source: BMC Public Health. 2019 Aug 14;19:1115. doi: 10.1186/s12889-019-7463-0 (PMC6694647; doi:10.1186/s12889-019-7463-0)
Supplement: Supplementary file 2 — Electronic search results and terms. This file contains a table of search results and terms used to retrieve studies from databases (DOCX 43 kb) [file 12889_2019_7463_MOESM2_ESM.docx]

**Additional File 2. Electronic search results and terms**

| Ovid Medline | 2016 |
| --- | --- |
| PsycInfo | 592 |
| CINAHL | 691 |
| PMC | 199 |
| Web of Science | 2081 |
| Scopus | 2561 |
| Sociological Abstracts | 133 |
| INFORMIT | 2353 |
| Cochrane | 2 |
| Embase | 3529 |
| SI | 200 |
| Total | 14366 |
| Pre 2006 | 143 |
| Duplicate papers | 7407 |
| Total left to screen | 6816 |

Database searches were conducted on the 24 May 2018. Gray literature searches were conducted between 24 May and 23 July 2018.

Database(s): **Ovid MEDLINE(R) Epub Ahead of Print, In-Process & Other Non-Indexed Citations, Ovid MEDLINE(R) Daily, Ovid MEDLINE and Versions(R)**

Search strategy:

| **#** | **Searches** |
| --- | --- |
| 1 | Oceanic Ancestry Group/ |
| 2 | Aborigin*.tw,kf. |
| 3 | (Torres Strait Islander or TSI or Torres or Strait).tw,kf. |
| 4 | First Nation*.tw,kf. |
| 5 | First People*.tw,kf. |
| 6 | Indigen*.tw,kf. |
| 7 | or/1-6 |
| 8 | exp Australia/ |
| 9 | (Australia* or victoria* or tasmania* or queensland* or new south wales or northern territory).tw,kf. |
| 10 | or/8-9 |
| 11 | exp Chronic Disease/ |
| 12 | (chronic disease* or chronic condition* or chronic illness* or non-communicable disease* or noncommunicable disease* or non communicable disease*).tw,kf. |
| 13 | Comorbidity/ |
| 14 | Multimorbidity/ |
| 15 | (comorbid* or multimorbid*).tw,kf. |
| 16 | exp Cardiovascular Diseases/ |
| 17 | ((coronary or heart or cardiac* or cardiovascula*) adj2 (syndrome* or disease* or event* or occlusion* or stenos* or thrombo* or attack*)).tw,kf. |
| 18 | (CHD or CVD or angina).tw,kf. |
| 19 | exp Respiratory Tract Diseases/ |
| 20 | ((lung* or pulmonary or bronchi* or airway or respiratory) adj3 (disease* or disorder*)).tw,kf. |
| 21 | (COPD* or asthma* or bronchiectasis or emphysema).tw,kf. |
| 22 | exp Endocrine System Diseases/ |
| 23 | ((endocrine or hormon* or thyroid or adrenal or pituitary or parathyroid or pancrea*) adj3 (disease* or disorder* or hyposecret* or hypersecret*)).tw,kf. |
| 24 | (hypothyroidism or hyperthyroidism or hyperparathyroidism or hypocyclemia or Hypopituitarism or cushing* or addison*).tw,kf. |
| 25 | exp Kidney Diseases/ |
| 26 | ((kidney or renal or urologic*) adj3 (disease* or disorder* or fail* or insufficien*)).tw,kf. |
| 27 | (ESRF or ESKF or ESRD or ESKD or CKF or CKD or CRF or CRD).tw,kf. |
| 28 | (predialysis or pre-dialysis).tw,kf. |
| 29 | (nephroma* or nephrit*).tw,kf. |
| 30 | exp Neoplasms/ |
| 31 | (cancer* or neoplas* or tumo* or carcinoma* or hodgkin* or nonhodgkin* or adenocarcinoma* or leuk?emia* or metasta* or malignan* or lymphoma* or sarcoma* or melanoma* or myeloma* or oncolog*).tw,kf. |
| 32 | exp Musculoskeletal Diseases/ |
| 33 | ((musculoskeletal* or bone or tendon* or joint* or cartilage or muscular or rheumati*) adj3 (disease* or disorder* or impair*)).tw,kf. |
| 34 | (osteoarthr* or Fibromyalgia or Tendonitis or tunnel syndrom*).tw,kf. |
| 35 | ((hip or knee or back or spinal or spine or neck or pelvis or pelvic) adj3 (problem* or degenerat* or disorder* or disease*)).tw,kf. |
| 36 | (disc* adj2 (herniat* or burst or ruptur* or bulg*)).tw,kf. |
| 37 | "nutritional and metabolic diseases"/ or exp metabolic diseases/ |
| 38 | ((nutrition* or metabolic or glucose) adj3 (disease* or disorder* or syndrome* or imbalance*)).tw,kf. |
| 39 | (diabet* or T2DM or DM or NIDDM or metabolic syndrome*).tw,kf. |
| 40 | (glyc?emic adj3 control*).tw,kf. |
| 41 | (insulin adj2 (sensitiv* or resistan*)).tw,kf. |
| 42 | Mental Health/ |
| 43 | Mental Disorders/ |
| 44 | exp anxiety disorders/ |
| 45 | exp mood disorders/ |
| 46 | (mental health or mental* ill* or mood disorder* or affective disorder* or regulation disorder* or depressive or depression or dysthymi* or anxiety or psychiatric).tw,kf. |
| 47 | exp Pain/ |
| 48 | pain*.tw,kf. |
| 49 | exp Tooth Diseases/ |
| 50 | ((tooth or teeth or oral or dental) adj2 (disorder* or disease* or abnormal*)).tw,kf. |
| 51 | exp Eye Diseases/ |
| 52 | ((eye* or optic* or cornea* or orbital or retina* or visual* or vision*) adj3 (disease* or disorder* or impair* or loss)).tw,kf. |
| 53 | (glaucoma or retinopath* or cataract*).tw,kf. |
| 54 | exp Liver Diseases/ |
| 55 | ((liver or hepati*) adj3 (disease* or disorder* or syndrome* or alcohol* or fail* or fatty)).tw,kf. |
| 56 | (hepatitis or cirrho*).tw,kf. |
| 57 | exp Life Style/ |
| 58 | ((healthy or unhealthy or sedentary or inactiv* or activ* or poor) adj3 (life style or lifestyle or diet* or nutrition* or eating or behav*)).tw,kf. |
| 59 | (physical activit* or exerc*).tw,kf. |
| 60 | exp Alcohol Drinking/ |
| 61 | (alcohol* or drinking).tw,kf. |
| 62 | exp Substance-Related Disorders/ |
| 63 | ((drug* or substance* or prescription*) adj3 (abus* or addiction* or dependence)).tw,kf. |
| 64 | (petrol* adj2 sniffing).tw,kf. |
| 65 | Health Behavior/ |
| 66 | Health Risk Behaviors/ |
| 67 | ((health* or unhealthy or risk*) adj5 behavio*).tw,kf. |
| 68 | exp Smoking/ |
| 69 | Smoking Cessation/ |
| 70 | Smoking Reduction/ |
| 71 | "Tobacco Use Cessation"/ |
| 72 | (tobacco or smoking or smoker* or cigarette*).tw,kf. |
| 73 | exp body weight/ |
| 74 | (overweight or unhealthy weight or obese or obesity or BMI or body mass index).tw,kf. |
| 75 | Risk Factors/ |
| 76 | or/11-75 |
| 77 | exp Health Education/ |
| 78 | Community Health Planning/ |
| 79 | exp Primary Health Care/ |
| 80 | (general practic* or primary health or clinic*).tw,kf. |
| 81 | Health Services/ |
| 82 | Health Services, Indigenous/ |
| 83 | Community Health Services/ |
| 84 | (communit* adj3 (led or based or directed)).tw,kf. |
| 85 | (health adj3 (planning or service* or education or literacy*)).tw,kf. |
| 86 | ((aborigin* or indigenous*) adj3 (co-op* or coop* or health or service* or organisation*)).tw,kf. |
| 87 | (ACCHOs or AMS or AHS or ACCHS).tw,kf. |
| 88 | (intervention* or project* or program* or strateg* or model* or initiative* or promotion*).tw,kf. |
| 89 | or/77-88 |
| 90 | and/7,10,76,89 |
| 91 | limit 90 to (english language and yr="2006 -Current") |
| 92 | exp child/ not (exp child/ and adult/) |
| 93 | adolescents/ not (adolescents/ and adults/) |
| 94 | (child* or infant* or teen* or youth* or adolescen* or p?ediatric*).ti. |
| 95 | exp animals/ not (exp animals/ and humans/) |
| 96 | or/92-95 |
| 97 | 91 not 96 |

Database(s): **PsycINFO**
Search Strategy:

| **#** | **Searches** |
| --- | --- |
| 1 | indigenous populations/ |
| 2 | Aborigin*.tw,id. |
| 3 | (Torres Strait Islander or TSI or Torres or Strait).tw,id. |
| 4 | First Nation*.tw,id. |
| 5 | First People*.tw,id. |
| 6 | Indigen*.tw,id. |
| 7 | or/1-6 |
| 8 | (Australia* or victoria* or tasmania* or queensland* or new south wales or northern territory).tw,id. |
| 9 | exp chronic illness/ or exp comorbidity/ |
| 10 | (chronic disease* or chronic condition* or chronic illness* or non-communicable disease* or noncommunicable disease* or non communicable disease*).tw,id. |
| 11 | exp cardiovascular disorders/ or Hypertension/ or Arteriosclerosis/ or Cholesterol/ or Lipids/ |
| 12 | ((coronary or heart or cardiac* or cardiovascula*) adj2 (syndrome* or disease* or event* or occlusion* or stenos* or thrombo* or attack*)).tw,id. |
| 13 | (CHD or CVD or angina).tw,id. |
| 14 | exp Respiratory Tract Disorders/ |
| 15 | ((lung* or pulmonary or bronchi* or airway or respiratory) adj3 (disease* or disorder*)).tw,id. |
| 16 | (COPD* or asthma* or bronchiectasis or emphysema).tw,id. |
| 17 | exp endocrine disorders/ |
| 18 | ((endocrine or hormon* or thyroid or adrenal or pituitary or parathyroid or pancrea*) adj3 (disease* or disorder* or hyposecret* or hypersecret*)).tw,id. |
| 19 | (hypothyroidism or hyperthyroidism or hyperparathyroidism or hypocyclemia or Hypopituitarism or cushing* or addison*).tw,id. |
| 20 | exp Kidney Diseases/ |
| 21 | ((kidney or renal or urologic*) adj3 (disease* or disorder* or fail* or insufficien*)).tw,id. |
| 22 | (ESRF or ESKF or ESRD or ESKD or CKF or CKD or CRF or CRD).tw,id. |
| 23 | (predialysis or pre-dialysis).tw,id. |
| 24 | (nephroma* or nephrit*).tw,id. |
| 25 | exp NEOPLASMS/ |
| 26 | (cancer* or neoplas* or tumo* or carcinoma* or hodgkin* or nonhodgkin* or adenocarcinoma* or leuk?emia* or metasta* or malignan* or lymphoma* or sarcoma* or melanoma* or myeloma* or oncolog*).tw,id. |
| 27 | exp Musculoskeletal Disorders/ |
| 28 | ((musculoskeletal* or bone or tendon* or joint* or cartilage or muscular or rheumati*) adj3 (disease* or disorder* or impair*)).tw,id. |
| 29 | (osteoarthr* or Fibromyalgia or Tendonitis or tunnel syndrom*).tw,id. |
| 30 | ((hip or knee or back or spinal or spine or neck or pelvis or pelvic) adj3 (problem* or degenerat* or disorder* or disease*)).tw,id. |
| 31 | (disc* adj2 (herniat* or burst or ruptur* or bulg*)).tw,id. |
| 32 | exp metabolism disorders/ |
| 33 | ((nutrition* or metabolic or glucose) adj3 (disease* or disorder* or syndrome* or imbalance*)).tw,id. |
| 34 | (diabet* or T2DM or DM or NIDDM or metabolic syndrome*).tw,id. |
| 35 | (glyc?emic adj3 control*).tw,id. |
| 36 | (insulin adj2 (sensitiv* or resistan*)).tw,id. |
| 37 | exp Mental Health/ |
| 38 | exp Anxiety Disorders/ |
| 39 | exp affective disorders/ |
| 40 | (mental health or mental* ill* or mood disorder* or affective disorder* or regulation disorder* or depressive or depression or dysthymi* or anxiety or psychiatric).tw,id. |
| 41 | exp PAIN/ |
| 42 | pain*.tw,id. |
| 43 | ((tooth or teeth or oral or dental) adj2 (disorder* or disease* or abnormal*)).tw,id. |
| 44 | exp Eye Disorders/ |
| 45 | ((eye* or optic* or cornea* or orbital or retina* or visual* or vision*) adj3 (disease* or disorder* or impair* or loss)).tw,id. |
| 46 | (glaucoma or retinopath* or cataract*).tw,id. |
| 47 | exp liver disorders/ |
| 48 | ((liver or hepati*) adj3 (disease* or disorder* or syndrome* or alcohol* or fail* or fatty)).tw,id. |
| 49 | (hepatitis or cirrho*).tw,id. |
| 50 | exp Lifestyle/ |
| 51 | ((healthy or unhealthy or sedentary or inactiv* or activ* or poor) adj3 (life style or lifestyle or diet* or nutrition* or eating or behav*)).tw,id. |
| 52 | (physical activit* or exerc*).tw,id. |
| 53 | exp alcohol drinking patterns/ |
| 54 | (alcohol* or drinking).tw,id. |
| 55 | exp Drug Abuse/ |
| 56 | ((drug* or substance* or prescription*) adj3 (abus* or addiction* or dependence)).tw,id. |
| 57 | (petrol* adj2 sniffing).tw,id. |
| 58 | exp Health Behavior/ |
| 59 | risk taking/ |
| 60 | ((health* or unhealthy or risk*) adj5 behavio*).tw,id. |
| 61 | Smoking Cessation/ |
| 62 | (tobacco or smoking or smoker* or cigarette*).tw,id. |
| 63 | exp body weight/ |
| 64 | (overweight or unhealthy weight or obese or obesity or BMI or body mass index).tw,id. |
| 65 | Risk Factors/ |
| 66 | or/9-65 |
| 67 | exp Health Education/ |
| 68 | Community Health Planning/ |
| 69 | exp Primary Health Care/ |
| 70 | (general practic* or primary health or clinic*).tw,id. |
| 71 | Health Care Services/ |
| 72 | Community Health/ |
| 73 | (communit* adj3 (led or based or directed)).tw,id. |
| 74 | (health adj3 (planning or service* or education or literacy*)).tw,id. |
| 75 | ((aborigin* or indigenous*) adj3 (co-op* or coop* or health or service* or organisation*)).tw,id. |
| 76 | (ACCHOs or AMS or AHS or ACCHS).tw,id. |
| 77 | (intervention* or project* or program* or strateg* or model* or initiative*).tw,id. |
| 78 | or/67-77 |
| 79 | 7 and 8 and 66 and 78 |
| 80 | limit 79 to (english language and yr="2006 -Current") |
| 81 | (child* or infant* or teen* or youth* or adolescen* or p?ediatric*).ti. |
| 82 | 80 not 81 |

Database(s): **CINAHL (EBSCOhost)**

Search strategy:

| **#** | **Query** |
| --- | --- |
| S1 | (MH "Indigenous Peoples") OR (MH "Aborigines") |
| S2 | TI Aborigin* OR AB Aborigin* |
| S3 | TI ( "Torres Strait Islander" OR TSI OR Torres OR Strait ) OR AB ( "Torres Strait Islander" OR TSI OR Torres OR Strait ) |
| S4 | TI "First Nation*" OR AB "First Nation*" |
| S5 | TI "First People*" OR AB "First People*" |
| S6 | TI Indigen* OR AB Indigen* |
| S7 | S1 OR S2 OR S3 OR S4 OR S5 OR S6 |
| S8 | (MH "Australia+") |
| S9 | TI ( Australia* OR victoria* OR tasmania* OR queensland* OR "new south wales" OR "northern territory" ) OR AB ( Australia* OR victoria* OR tasmania* OR queensland* OR "new south wales" OR "northern territory" ) |
| S10 | S8 OR S9 |
| S11 | (MH "Chronic Disease") |
| S12 | TI ( "chronic disease*" OR "chronic condition*" OR "chronic illness*" OR "non-communicable disease*" OR "noncommunicable disease*" OR "non communicable disease*" ) OR AB ( "chronic disease*" OR "chronic condition*" OR "chronic illness*" OR "non-communicable disease*" OR "noncommunicable disease*" OR "non communicable disease*" ) |
| S13 | (MH "Comorbidity") |
| S14 | TI ( comorbid* OR multimorbid* ) OR AB ( comorbid* OR multimorbid* ) |
| S15 | (MH "Cardiovascular Diseases+") |
| S16 | TI ( (coronary OR heart OR cardiac* OR cardiovascula*) N2 (syndrome* OR disease* OR event* OR occlusion* OR stenos* OR thrombo* OR attack*) ) OR AB ( (coronary OR heart OR cardiac* OR cardiovascula*) N2 (syndrome* OR disease* OR event* OR occlusion* OR stenos* OR thrombo* OR attack*) ) |
| S17 | TI ( CHD OR CVD OR angina ) OR AB ( CHD OR CVD OR angina ) |
| S18 | (MH "Respiratory Tract Diseases+") |
| S19 | TI ( (lung* OR pulmonary OR bronchi* OR airway OR respiratory) N3 (disease* OR disorder*) ) OR AB ( (lung* OR pulmonary OR bronchi* OR airway OR respiratory) N3 (disease* OR disorder*) ) |
| S20 | TI ( COPD* OR asthma* OR bronchiectasis OR emphysema ) OR AB ( COPD* OR asthma* OR bronchiectasis OR emphysema ) |
| S21 | (MH "Endocrine Diseases+") |
| S22 | TI ( (endocrine OR hormon* OR thyroid OR adrenal OR pituitary OR parathyroid OR pancrea*) N3 (disease* OR disorder* OR hyposecret* OR hypersecret*) ) OR AB ( (endocrine OR hormon* OR thyroid OR adrenal OR pituitary OR parathyroid OR pancrea*) N3 (disease* OR disorder* OR hyposecret* OR hypersecret*) ) |
| S23 | TI ( hypothyroidism OR hyperthyroidism OR hyperparathyroidism OR hypocyclemia OR Hypopituitarism OR cushing* OR addison* ) OR AB ( hypothyroidism OR hyperthyroidism OR hyperparathyroidism OR hypocyclemia OR Hypopituitarism OR cushing* OR addison* ) |
| S24 | (MH "Kidney Diseases+") |
| S25 | TI ( (kidney OR renal OR urologic*) N3 (disease* OR disorder* OR fail* OR insufficien*) ) OR AB ( (kidney OR renal OR urologic*) N3 (disease* OR disorder* OR fail* OR insufficien*) ) |
| S26 | TI ( ESRF OR ESKF OR ESRD OR ESKD OR CKF OR CKD OR CRF OR CRD ) OR AB ( ESRF OR ESKF OR ESRD OR ESKD OR CKF OR CKD OR CRF OR CRD ) |
| S27 | TI ( predialysis OR pre-dialysis ) OR AB ( predialysis OR pre-dialysis ) |
| S28 | TI ( nephroma* OR nephrit* ) OR AB ( nephroma* OR nephrit* ) |
| S29 | (MH "Neoplasms+") |
| S30 | TI ( cancer* OR neoplas* OR tumo* OR carcinoma* OR hodgkin* OR nonhodgkin* OR adenocarcinoma* OR leuk?emia* OR metasta* OR malignan* OR lymphoma* OR sarcoma* OR melanoma* OR myeloma* OR oncolog* ) OR AB ( cancer* OR neoplas* OR tumo* OR carcinoma* OR hodgkin* OR nonhodgkin* OR adenocarcinoma* OR leuk?emia* OR metasta* OR malignan* OR lymphoma* OR sarcoma* OR melanoma* OR myeloma* OR oncolog* ) |
| S31 | (MH "Musculoskeletal Diseases+") |
| S32 | TI ( (musculoskeletal* OR bone OR tendon* OR joint* OR cartilage OR muscular OR rheumati*) N3 (disease* OR disorder* OR impair*) ) OR AB ( (musculoskeletal* OR bone OR tendon* OR joint* OR cartilage OR muscular OR rheumati*) N3 (disease* OR disorder* OR impair*) ) |
| S33 | TI ( osteoarthr* OR Fibromyalgia OR Tendonitis OR "tunnel syndrom*" ) OR AB ( osteoarthr* OR Fibromyalgia OR Tendonitis OR "tunnel syndrom*" ) |
| S34 | TI ( (hip OR knee OR back OR spinal OR spine OR neck OR pelvis OR pelvic) N3 (problem* OR degenerat* OR disorder* OR disease*) ) OR AB ( (hip OR knee OR back OR spinal OR spine OR neck OR pelvis OR pelvic) N3 (problem* OR degenerat* OR disorder* OR disease*) ) |
| S35 | TI ( disc* N2 (herniat* OR burst OR ruptur* OR bulg*) ) OR AB ( disc* N2 (herniat* OR burst OR ruptur* OR bulg*) ) |
| S36 | (MH "Nutritional and Metabolic Diseases") OR (MH "Metabolic Diseases+") |
| S37 | TI ( (nutrition* OR metabolic OR glucose) N3 (disease* OR disorder* OR syndrome* OR imbalance*) ) OR AB ( (nutrition* OR metabolic OR glucose) N3 (disease* OR disorder* OR syndrome* OR imbalance*) ) |
| S38 | TI ( diabet* OR T2DM OR DM OR NIDDM OR "metabolic syndrome*" ) OR AB ( diabet* OR T2DM OR DM OR NIDDM OR "metabolic syndrome*" ) |
| S39 | TI glyc*emic N3 control* OR AB glyc*emic N3 control* |
| S40 | TI ( insulin N2 (sensitiv* OR resistan*) ) OR AB ( insulin N2 (sensitiv* OR resistan*) ) |
| S41 | (MH "Mental Health") |
| S42 | (MH "Mental Disorders") |
| S43 | (MH "Anxiety Disorders+") |
| S44 | (MH "Affective Disorders+") |
| S45 | TI ( "mental health" OR "mental* ill*" OR "mood disorder*" OR "affective disorder*" OR "regulation disorder*" OR depressive OR depression OR dysthymi* OR anxiety OR psychiatric ) OR AB ( "mental health" OR "mental* ill*" OR "mood disorder*" OR "affective disorder*" OR "regulation disorder*" OR depressive OR depression OR dysthymi* OR anxiety OR psychiatric ) |
| S46 | (MH "Pain+") |
| S47 | TI pain* OR AB pain* |
| S48 | (MH "Tooth Diseases+") |
| S49 | TI ( (tooth OR teeth OR oral OR dental) N2 (disorder* OR disease* OR abnormal*) ) OR AB ( (tooth OR teeth OR oral OR dental) N2 (disorder* OR disease* OR abnormal*) ) |
| S50 | (MH "Eye Diseases+") |
| S51 | TI ( (eye* OR optic* OR cornea* OR orbital OR retina* OR visual* OR vision*) N3 (disease* OR disorder* OR impair* OR loss) ) OR AB ( (eye* OR optic* OR cornea* OR orbital OR retina* OR visual* OR vision*) N3 (disease* OR disorder* OR impair* OR loss) ) |
| S52 | TI ( glaucoma OR retinopath* OR cataract* ) OR AB ( glaucoma OR retinopath* OR cataract* ) |
| S53 | (MH "Liver Diseases+") |
| S54 | TI ( (liver OR hepati*) N3 (disease* OR disorder* OR syndrome* OR alcohol* OR fail* OR fatty) ) OR AB ( (liver OR hepati*) N3 (disease* OR disorder* OR syndrome* OR alcohol* OR fail* OR fatty) ) |
| S55 | TI ( hepatitis OR cirrho* ) OR AB ( hepatitis OR cirrho* ) |
| S56 | (MH "Life Style+") |
| S57 | TI ( (healthy OR unhealthy OR sedentary OR inactiv* OR activ* OR poor) N3 (life style OR lifestyle OR diet* OR nutrition* OR eating OR behav*) ) OR AB ( (healthy OR unhealthy OR sedentary OR inactiv* OR activ* OR poor) N3 (life style OR lifestyle OR diet* OR nutrition* OR eating OR behav*) ) |
| S58 | TI ( "physical activit*" OR exerc* ) OR AB ( "physical activit*" OR exerc* ) |
| S59 | (MH "Alcohol Drinking+") |
| S60 | TI ( alcohol* OR drinking ) OR AB ( alcohol* OR drinking ) |
| S61 | (MH "Substance Use Disorders+") |
| S62 | TI ( (drug* OR substance* OR prescription*) N3 (abus* OR addiction* OR dependence) ) OR AB ( (drug* OR substance* OR prescription*) N3 (abus* OR addiction* OR dependence) ) |
| S63 | TI petrol* N2 sniffing OR AB petrol* N2 sniffing |
| S64 | (MH "Health Behavior") |
| S65 | (MH "Risk Taking Behavior+") |
| S66 | TI ( (health* OR unhealthy OR risk*) N5 behavio* ) OR AB ( (health* OR unhealthy OR risk*) N5 behavio* ) |
| S67 | (MH "Smoking+") |
| S68 | (MH "Smoking Cessation") |
| S69 | TI ( tobacco OR smoking OR smoker* OR cigarette* ) OR AB ( tobacco OR smoking OR smoker* OR cigarette* ) |
| S70 | (MH "Body Weight+") |
| S71 | TI ( overweight OR "unhealthy weight" OR obese OR obesity OR BMI OR "body mass index" ) OR AB ( overweight OR "unhealthy weight" OR obese OR obesity OR BMI OR "body mass index" ) |
| S72 | (MH "Risk Factors") OR (MH "Cardiovascular Risk Factors") |
| S73 | S11 OR S12 OR S13 OR S14 OR S15 OR S16 OR S17 OR S18 OR S19 OR S20 OR S21 OR S22 OR S23 OR S24 OR S25 OR S26 OR S27 OR S28 OR S29 OR S30 OR S31 OR S32 OR S33 OR S34 OR S35 OR S36 OR S37 OR S38 OR S39 OR S40 OR S41 OR S42 OR S43 OR S44 OR S45 OR S46 OR S47 OR S48 OR S49 OR S50 OR S51 OR S52 OR S53 OR S54 OR S55 OR S56 OR S57 OR S58 OR S59 OR S60 OR S61 OR S62 OR S63 OR S64 OR S65 OR S66 OR S67 OR S68 OR S69 OR S70 OR S71 OR S72 |
| S74 | (MH "Health Education+") |
| S75 | (MH "Primary Health Care") |
| S76 | TI ( "general practic*" OR "primary health" OR clinic* ) OR AB ( "general practic*" OR "primary health" OR clinic* ) |
| S77 | (MH "Health Services") |
| S78 | (MH "Health Services, Indigenous") |
| S79 | (MH "Community Health Services") |
| S80 | TI ( communit* N3 (led OR based OR directed) ) OR AB ( communit* N3 (led OR based OR directed) ) |
| S81 | TI ( health N3 (planning OR service* OR education OR literacy*) ) OR AB ( health N3 (planning OR service* OR education OR literacy*) ) |
| S82 | TI ( (aborigin* OR indigenous*) N3 (co-op* OR coop* OR health OR service* OR organisation*) ) OR AB ( (aborigin* OR indigenous*) N3 (co-op* OR coop* OR health OR service* OR organisation*) ) |
| S83 | TI ( ACCHOs OR AMS OR AHS OR ACCHS ) OR AB ( ACCHOs OR AMS OR AHS OR ACCHS ) |
| S84 | TI ( intervention* OR project* OR program* OR strateg* OR model* OR initiative* OR promotion* ) OR AB ( intervention* OR project* OR program* OR strateg* OR model* OR initiative* OR promotion* ) |
| S85 | S74 OR S75 OR S76 OR S77 OR S78 OR S79 OR S80 OR S81 OR S82 OR S83 OR S84 |
| S86 | S7 AND S10 AND S73 AND S85 |
| S87 | ((MH "Child+") NOT ((MH "Child+") AND (MH "Adult+"))) |
| S88 | ((MH "Adolescence") NOT ((MH "Adolescence") AND (MH "Adult+"))) |
| S89 | TI child* OR infant* OR teen* OR youth* OR adolescen* OR pediatric* OR paediatric* |
| S90 | ((MH "Animals+") NOT ((MH "Animals+") AND (MH "Human") )) |
| S91 | S87 OR S88 OR S89 OR S90 |
| S92 | S86 NOT S91 |

Database(s): **Embase**

Search strategy:

| **#** | **Query** |
| --- | --- |
| 1 | 'australian aborigine'/exp |
| 2 | 'indigenous people'/de OR 'indigenous australian'/de |
| 3 | 'aborigin*':ti,ab,kw OR 'torres strait islander':ti,ab,kw OR 'tsi':ti,ab,kw OR 'torres':ti,ab,kw OR 'strait':ti,ab,kw OR 'first nation*':ti,ab,kw OR 'first people*':ti,ab,kw OR 'indigen*':ti,ab,kw |
| 4 | #1 OR #2 OR #3 |
| 5 | 'australia'/exp |
| 6 | 'australia*':ti,ab,kw OR 'victoria*':ti,ab,kw OR 'tasmania*':ti,ab,kw OR 'queensland*':ti,ab,kw OR 'new south wales':ti,ab,kw OR 'northern territory':ti,ab,kw |
| 7 | #5 OR #6 |
| 8 | 'chronic disease'/exp |
| 9 | 'chronic disease*':ti,ab,kw OR 'chronic condition*':ti,ab,kw OR 'chronic illness*':ti,ab,kw OR 'non-communicable disease*':ti,ab,kw OR 'noncommunicable disease*':ti,ab,kw OR 'non communicable disease*':ti,ab,kw OR 'comorbid*':ti,ab,kw OR 'multimorbid*':ti,ab,kw |
| 10 | '((coronary':ti,ab,kw OR 'heart':ti,ab,kw OR 'cardiac*':ti,ab,kw OR (('cardiovascula*)' NEAR/2 '(syndrome*'):ti,ab,kw) OR 'disease*':ti,ab,kw OR 'event*':ti,ab,kw OR 'occlusion*':ti,ab,kw OR 'stenos*':ti,ab,kw OR 'thrombo*':ti,ab,kw OR 'attack*))':ti,ab,kw |
| 11 | 'chd':ti,ab,kw OR 'cvd':ti,ab,kw OR 'angina':ti,ab,kw |
| 12 | '((lung*':ti,ab,kw OR 'pulmonary':ti,ab,kw OR 'bronchi*':ti,ab,kw OR 'airway':ti,ab,kw OR (('respiratory)' NEAR/3 '(disease*'):ti,ab,kw) OR 'disorder*))':ti,ab,kw |
| 13 | 'copd*':ti,ab,kw OR 'asthma*':ti,ab,kw OR 'bronchiectasis':ti,ab,kw OR 'emphysema':ti,ab,kw |
| 14 | '((endocrine':ti,ab,kw OR 'hormon*':ti,ab,kw OR 'thyroid':ti,ab,kw OR 'adrenal':ti,ab,kw OR 'pituitary':ti,ab,kw OR 'parathyroid':ti,ab,kw OR (('pancrea*)' NEAR/3 '(disease*'):ti,ab,kw) OR 'disorder*':ti,ab,kw OR 'hyposecret*':ti,ab,kw OR 'hypersecret*))':ti,ab,kw |
| 15 | 'hypothyroidism':ti,ab,kw OR 'hyperthyroidism':ti,ab,kw OR 'hyperparathyroidism':ti,ab,kw OR 'hypocyclemia':ti,ab,kw OR 'hypopituitarism':ti,ab,kw OR 'cushing*':ti,ab,kw OR 'addison*':ti,ab,kw |
| 16 | '((kidney':ti,ab,kw OR 'renal':ti,ab,kw OR (('urologic*)' NEAR/3 '(disease*'):ti,ab,kw) OR 'disorder*':ti,ab,kw OR 'fail*':ti,ab,kw OR 'insufficien*))':ti,ab,kw |
| 17 | '((musculoskeletal*':ti,ab,kw OR 'bone':ti,ab,kw OR 'tendon*':ti,ab,kw OR 'joint*':ti,ab,kw OR 'cartilage':ti,ab,kw OR 'muscular':ti,ab,kw OR (('rheumati*)' NEAR/3 '(disease*'):ti,ab,kw) OR 'disorder*':ti,ab,kw OR 'impair*))':ti,ab,kw |
| 18 | 'osteoarthr*':ti,ab,kw OR 'fibromyalgia':ti,ab,kw OR 'tendonitis':ti,ab,kw OR 'tunnel syndrom*':ti,ab,kw |
| 19 | '((hip':ti,ab,kw OR 'knee':ti,ab,kw OR 'back':ti,ab,kw OR 'spinal':ti,ab,kw OR 'spine':ti,ab,kw OR 'neck':ti,ab,kw OR 'pelvis':ti,ab,kw OR (('pelvic)' NEAR/3 '(problem*'):ti,ab,kw) OR 'degenerat*':ti,ab,kw OR 'disorder*':ti,ab,kw OR 'disease*))':ti,ab,kw OR (('(disc*' NEAR/2 '(herniat*'):ti,ab,kw) OR 'burst':ti,ab,kw OR 'ruptur*':ti,ab,kw OR 'bulg*))':ti,ab,kw |
| 20 | '((nutrition*':ti,ab,kw OR 'metabolic':ti,ab,kw OR (('glucose)' NEAR/3 '(disease*'):ti,ab,kw) OR 'disorder*':ti,ab,kw OR 'syndrome*':ti,ab,kw OR 'imbalance*))':ti,ab,kw |
| 21 | 'diabet*':ti,ab,kw OR 't2dm':ti,ab,kw OR 'dm':ti,ab,kw OR 'niddm':ti,ab,kw OR 'metabolic syndrome*':ti,ab,kw |
| 22 | ('(glyc?emic' NEAR/3 'control*)'):ti,ab,kw |
| 23 | (('(insulin' NEAR/2 '(sensitiv*'):ti,ab,kw) OR 'resistan*))':ti,ab,kw |
| 24 | 'mental health':ti,ab,kw OR 'mental* ill*':ti,ab,kw OR 'mood disorder*':ti,ab,kw OR 'affective disorder*':ti,ab,kw OR 'regulation disorder*':ti,ab,kw OR 'depressive':ti,ab,kw OR 'depression':ti,ab,kw OR 'dysthymi*':ti,ab,kw OR 'anxiety':ti,ab,kw OR 'psychiatric':ti,ab,kw OR 'pain*':ti,ab,kw |
| 25 | '((tooth':ti,ab,kw OR 'teeth':ti,ab,kw OR 'oral':ti,ab,kw OR (('dental)' NEAR/2 '(disorder*'):ti,ab,kw) OR 'disease*':ti,ab,kw OR 'abnormal*))':ti,ab,kw |
| 26 | '((eye*':ti,ab,kw OR 'optic*':ti,ab,kw OR 'cornea*':ti,ab,kw OR 'orbital':ti,ab,kw OR 'retina*':ti,ab,kw OR 'visual*':ti,ab,kw OR (('vision*)'NEAR/3 '(disease*'):ti,ab,kw) OR 'disorder*':ti,ab,kw OR 'impair*':ti,ab,kw OR 'loss))':ti,ab,kw |
| 27 | 'glaucoma':ti,ab,kw OR 'retinopath*':ti,ab,kw OR 'cataract*':ti,ab,kw |
| 28 | '((liver':ti,ab,kw OR (('hepati*)' NEAR/3 '(disease*'):ti,ab,kw) OR 'disorder*':ti,ab,kw OR 'syndrome*':ti,ab,kw OR 'alcohol*':ti,ab,kw OR 'fail*':ti,ab,kw OR 'fatty))':ti,ab,kw |
| 29 | 'hepatitis':ti,ab,kw OR 'cirrho*':ti,ab,kw |
| 30 | '((healthy':ti,ab,kw OR 'unhealthy':ti,ab,kw OR 'sedentary':ti,ab,kw OR 'inactiv*':ti,ab,kw OR 'activ*':ti,ab,kw OR (('poor)' NEAR/3 '(life style'):ti,ab,kw) OR 'lifestyle':ti,ab,kw OR 'diet*':ti,ab,kw OR 'nutrition*':ti,ab,kw OR 'eating':ti,ab,kw OR 'behavior))':ti,ab,kw |
| 31 | 'physical activit*':ti,ab,kw OR 'exerc*':ti,ab,kw OR 'alcohol*':ti,ab,kw OR 'drinking':ti,ab,kw |
| 32 | '((drug*':ti,ab,kw OR 'substance*':ti,ab,kw OR (('prescription*)' NEAR/3 '(abus*'):ti,ab,kw) OR 'addiction*':ti,ab,kw OR 'dependence))':ti,ab,kw |
| 33 | ('(petrol*' NEAR/2 'sniffing)'):ti,ab,kw |
| 34 | '((health*':ti,ab,kw OR 'unhealthy':ti,ab,kw OR (('risk*)' NEAR/5 'behavio*)'):ti,ab,kw) |
| 35 | 'tobacco':ti,ab,kw OR 'smoking':ti,ab,kw OR 'smoker*':ti,ab,kw OR 'cigarette*':ti,ab,kw OR 'overweight':ti,ab,kw OR 'unhealthy weight':ti,ab,kw OR 'obese':ti,ab,kw OR 'obesity':ti,ab,kw OR 'bmi':ti,ab,kw OR 'body mass index':ti,ab,kw |
| 36 | 'comorbidity'/exp |
| 37 | 'cardiovascular disease'/exp |
| 38 | 'respiratory tract disease'/exp |
| 39 | 'endocrine disease'/exp |
| 40 | 'kidney disease'/exp |
| 41 | 'neoplasm'/exp |
| 42 | 'musculoskeletal disease'/exp |
| 43 | 'metabolic disorder'/exp |
| 44 | 'mental health'/exp |
| 45 | 'mental disease'/de |
| 46 | 'mood disorder'/exp OR 'depression'/exp OR 'anxiety disorder'/exp |
| 47 | 'pain'/exp |
| 48 | 'tooth disease'/exp |
| 49 | 'eye disease'/exp |
| 50 | 'liver disease'/exp |
| 51 | 'lifestyle'/exp |
| 52 | 'drinking behavior'/exp |
| 53 | 'drug dependence'/exp |
| 54 | 'health behavior'/exp |
| 55 | 'smoking'/exp |
| 56 | 'smoking cessation'/de OR 'smoking reduction'/de |
| 57 | 'body weight'/exp |
| 58 | 'risk factor'/de |
| 59 | #8 OR #9 OR #10 OR #11 OR #12 OR #13 OR #14 OR #15 OR #16 OR #17 OR #18 OR #19 OR #20 OR #21 OR #22 OR #23 OR #24 OR #25 OR #26 OR #27 OR #28 OR #29 OR #30 OR #31 OR #32 OR #33 OR #34 OR #35 OR #36 OR #37 OR #38 OR #39 OR #40 OR #41 OR #42 OR #43OR #44 OR #45 OR #46 OR #47 OR #48 OR #49 OR #50 OR #51 OR #52 OR #53 OR #54 OR #55 OR #56 OR #57 OR #58 |
| 60 | 'health education'/exp |
| 61 | 'primary health care'/exp |
| 62 | 'general practic*':ti,ab,kw OR 'primary health':ti,ab,kw OR 'clinic*':ti,ab,kw |
| 63 | 'health service'/de |
| 64 | 'indigenous health care'/de |
| 65 | 'community care'/exp |
| 66 | (('communit*' NEAR/3 '(led'):ti,ab,kw) OR 'based':ti,ab,kw OR 'directed))':ti,ab,kw |
| 67 | (('(health' NEAR/3 '(planning'):ti,ab,kw) OR 'service*':ti,ab,kw OR 'education':ti,ab,kw OR 'literacy*))':ti,ab,kw |
| 68 | '((aborigin*':ti,ab,kw OR (('indigenous*)' NEAR/3 '(co-op*'):ti,ab,kw) OR 'coop*':ti,ab,kw OR 'health':ti,ab,kw OR 'service*':ti,ab,kw OR 'organisation*))':ti,ab,kw |
| 69 | 'acchos':ti,ab,kw OR 'ams':ti,ab,kw OR 'ahs':ti,ab,kw OR 'acchs':ti,ab,kw OR 'intervention*':ti,ab,kw OR 'project*':ti,ab,kw OR 'program*':ti,ab,kw OR 'strateg*':ti,ab,kw OR 'model*':ti,ab,kw OR 'initiative*':ti,ab,kw OR 'promotion*':ti,ab,kw |
| 70 | #60 OR #61 OR #62 OR #63 OR #64 OR #65 OR #66 OR #67 OR #68 OR #69 |
| 71 | #4 AND #7 AND #59 AND #70 |
| 72 | #4 AND #7 AND #59 AND #70 AND [english]/lim AND [embase]/lim AND [2006-2018]/py |
| 73 | 'child*':ti OR 'infant*':ti OR 'teen*':ti OR 'youth*':ti OR 'adolescen*':ti OR 'p?ediatric*':ti |
| 74 | #72 NOT #73 |

# Database(s):SocINDEX

# Search Strategy:

| **#** | **Query** |
| --- | --- |
| S1 | TI Aborigin* OR AB Aborigin* OR KW Aborigin* |
| S2 | TI ( "Torres Strait Islander" OR TSI OR Torres OR Strait ) OR AB ( "Torres Strait Islander" OR TSI OR Torres OR Strait ) OR KW ( "Torres Strait Islander" OR TSI OR Torres OR Strait ) |
| S3 | TI "First Nation*" OR AB "First Nation*" OR KW "First Nation*" |
| S4 | TI "First People*" OR AB "First People*" OR KW "First People*" |
| S5 | TI Indigen* OR AB Indigen* OR KW Indigen* |
| S6 | (S1 OR S2 OR S3 OR S4 OR S5) |
| S7 | TI ( Australia* OR victoria* OR tasmania* OR queensland* OR "new south wales" OR "northern territory" ) OR AB ( Australia* OR victoria* OR tasmania* OR queensland* OR "new south wales" OR "northern territory" ) OR KW ( Australia* OR victoria* OR tasmania* OR queensland* OR "new south wales" OR "northern territory" ) |
| S8 | TI ( "chronic disease*" OR "chronic condition*" OR "chronic illness*" OR "non-communicable disease*" OR "noncommunicable disease*" OR "non communicable disease*" ) OR AB ( "chronic disease*" OR "chronic condition*" OR "chronic illness*" OR "non-communicable disease*" OR "noncommunicable disease*" OR "non communicable disease*" ) OR KW ( "chronic disease*" OR "chronic condition*" OR "chronic illness*" OR "non-communicable disease*" OR "noncommunicable disease*" OR "non communicable disease*" ) |
| S9 | TI ( comorbid* OR multimorbid* ) OR AB ( comorbid* OR multimorbid* ) OR KW ( comorbid* OR multimorbid* ) |
| S10 | TI ( (coronary OR heart OR cardiac* OR cardiovascula*) N2 (syndrome* OR disease* OR event* OR occlusion* OR stenos* OR thrombo* OR attack*) ) OR AB ( (coronary OR heart OR cardiac* OR cardiovascula*) N2 (syndrome* OR disease* OR event* OR occlusion* OR stenos* OR thrombo* OR attack*) ) OR KW ( (coronary OR heart OR cardiac* OR cardiovascula*) N2 (syndrome* OR disease* OR event* OR occlusion* OR stenos* OR thrombo* OR attack*) ) |
| S11 | TI ( CHD OR CVD OR angina ) OR AB ( CHD OR CVD OR angina ) OR KW ( CHD OR CVD OR angina ) |
| S12 | TI ( (lung* OR pulmonary OR bronchi* OR airway OR respiratory) N3 (disease* OR disorder*) ) OR AB ( (lung* OR pulmonary OR bronchi* OR airway OR respiratory) N3 (disease* OR disorder*) ) OR KW ( (lung* OR pulmonary OR bronchi* OR airway OR respiratory) N3 (disease* OR disorder*) ) |
| S13 | TI ( COPD* OR asthma* OR bronchiectasis OR emphysema ) OR AB ( COPD* OR asthma* OR bronchiectasis OR emphysema ) OR KW ( COPD* OR asthma* OR bronchiectasis OR emphysema ) |
| S14 | TI ( (endocrine OR hormon* OR thyroid OR adrenal OR pituitary OR parathyroid OR pancrea*) N3 (disease* OR disorder* OR hyposecret* OR hypersecret*) ) OR AB ( (endocrine OR hormon* OR thyroid OR adrenal OR pituitary OR parathyroid OR pancrea*) N3 (disease* OR disorder* OR hyposecret* OR hypersecret*) ) OR KW ( (endocrine OR hormon* OR thyroid OR adrenal OR pituitary OR parathyroid OR pancrea*) N3 (disease* OR disorder* OR hyposecret* OR hypersecret*) ) |
| S15 | TI ( hypothyroidism OR hyperthyroidism OR hyperparathyroidism OR hypocyclemia OR Hypopituitarism OR cushing* OR addison* ) OR AB ( hypothyroidism OR hyperthyroidism OR hyperparathyroidism OR hypocyclemia OR Hypopituitarism OR cushing* OR addison* ) OR KW ( hypothyroidism OR hyperthyroidism OR hyperparathyroidism OR hypocyclemia OR Hypopituitarism OR cushing* OR addison* ) |
| S16 | TI ( (kidney OR renal OR urologic*) N3 (disease* OR disorder* OR fail* OR insufficien*) ) OR AB ( (kidney OR renal OR urologic*) N3 (disease* OR disorder* OR fail* OR insufficien*) ) OR KW ( (kidney OR renal OR urologic*) N3 (disease* OR disorder* OR fail* OR insufficien*) ) |
| S17 | TI ( ESRF OR ESKF OR ESRD OR ESKD OR CKF OR CKD OR CRF OR CRD ) OR AB ( ESRF OR ESKF OR ESRD OR ESKD OR CKF OR CKD OR CRF OR CRD ) OR KW ( ESRF OR ESKF OR ESRD OR ESKD OR CKF OR CKD OR CRF OR CRD ) |
| S18 | TI ( predialysis OR pre-dialysis ) OR AB ( predialysis OR pre-dialysis ) OR KW ( predialysis OR pre-dialysis ) |
| S19 | TI ( nephroma* OR nephrit* ) OR AB ( nephroma* OR nephrit* ) OR KW ( nephroma* OR nephrit* ) |
| S20 | TI ( cancer* OR neoplas* OR tumo* OR carcinoma* OR hodgkin* OR nonhodgkin* OR adenocarcinoma* OR leuk?emia* OR metasta* OR malignan* OR lymphoma* OR sarcoma* OR melanoma* OR myeloma* OR oncolog* ) OR AB ( cancer* OR neoplas* OR tumo* OR carcinoma* OR hodgkin* OR nonhodgkin* OR adenocarcinoma* OR leuk?emia* OR metasta* OR malignan* OR lymphoma* OR sarcoma* OR melanoma* OR myeloma* OR oncolog* ) OR KW ( cancer* OR neoplas* OR tumo* OR carcinoma* OR hodgkin* OR nonhodgkin* OR adenocarcinoma* OR leuk?emia* OR metasta* OR malignan* OR lymphoma* OR sarcoma* OR melanoma* OR myeloma* OR oncolog* ) |
| S21 | TI ( (musculoskeletal* OR bone OR tendon* OR joint* OR cartilage OR muscular OR rheumati*) N3 (disease* OR disorder* OR impair*) ) OR AB ( (musculoskeletal* OR bone OR tendon* OR joint* OR cartilage OR muscular OR rheumati*) N3 (disease* OR disorder* OR impair*) ) OR KW ( (musculoskeletal* OR bone OR tendon* OR joint* OR cartilage OR muscular OR rheumati*) N3 (disease* OR disorder* OR impair*) ) |
| S22 | TI ( osteoarthr* OR Fibromyalgia OR Tendonitis OR "tunnel syndrom*" ) OR AB ( osteoarthr* OR Fibromyalgia OR Tendonitis OR "tunnel syndrom*" ) OR KW ( osteoarthr* OR Fibromyalgia OR Tendonitis OR "tunnel syndrom*" ) |
| S23 | TI ( (hip OR knee OR back OR spinal OR spine OR neck OR pelvis OR pelvic) N3 (problem* OR degenerat* OR disorder* OR disease*) ) OR AB ( (hip OR knee OR back OR spinal OR spine OR neck OR pelvis OR pelvic) N3 (problem* OR degenerat* OR disorder* OR disease*) ) OR KW ( (hip OR knee OR back OR spinal OR spine OR neck OR pelvis OR pelvic) N3 (problem* OR degenerat* OR disorder* OR disease*) ) |
| S24 | TI ( disc* N2 (herniat* OR burst OR ruptur* OR bulg*) ) OR AB ( disc* N2 (herniat* OR burst OR ruptur* OR bulg*) ) OR KW ( disc* N2 (herniat* OR burst OR ruptur* OR bulg*) ) |
| S25 | TI ( (nutrition* OR metabolic OR glucose) N3 (disease* OR disorder* OR syndrome* OR imbalance*) ) OR AB ( (nutrition* OR metabolic OR glucose) N3 (disease* OR disorder* OR syndrome* OR imbalance*) ) OR KW ( (nutrition* OR metabolic OR glucose) N3 (disease* OR disorder* OR syndrome* OR imbalance*) ) |
| S26 | TI ( diabet* OR T2DM OR DM OR NIDDM OR "metabolic syndrome*" ) OR AB ( diabet* OR T2DM OR DM OR NIDDM OR "metabolic syndrome*" ) OR KW ( diabet* OR T2DM OR DM OR NIDDM OR "metabolic syndrome*" ) |
| S27 | TI glyc*emic N3 control* OR AB glyc*emic N3 control* OR KW glyc*emic N3 control* |
| S28 | TI ( insulin N2 (sensitiv* OR resistan*) ) OR AB ( insulin N2 (sensitiv* OR resistan*) ) OR KW ( insulin N2 (sensitiv* OR resistan*) ) |
| S29 | TI ( ( "mental health" OR "mental* ill*" OR "mood disorder*" OR "affective disorder*" OR "regulation disorder*" OR depressive OR depression OR dysthymi* OR anxiety OR psychiatric ) OR AB ( ( "mental health" OR "mental* ill*" OR "mood disorder*" OR "affective disorder*" OR "regulation disorder*" OR depressive OR depression OR dysthymi* OR anxiety OR psychiatric ) OR KW ( ( "mental health" OR "mental* ill*" OR "mood disorder*" OR "affective disorder*" OR "regulation disorder*" OR depressive OR depression OR dysthymi* OR anxiety OR psychiatric ) |
| S30 | TI pain* OR AB pain* OR KW pain* |
| S31 | TI ( (tooth OR teeth OR oral OR dental) N2 (disorder* OR disease* OR abnormal*) ) OR AB ( (tooth OR teeth OR oral OR dental) N2 (disorder* OR disease* OR abnormal*) ) OR KW ( (tooth OR teeth OR oral OR dental) N2 (disorder* OR disease* OR abnormal*) ) |
| S32 | TI ( ( (eye* OR optic* OR cornea* OR orbital OR retina* OR visual* OR vision*) N3 (disease* OR disorder* OR impair* OR loss) ) OR AB ( ( (eye* OR optic* OR cornea* OR orbital OR retina* OR visual* OR vision*) N3 (disease* OR disorder* OR impair* OR loss) ) OR KW ( ( (eye* OR optic* OR cornea* OR orbital OR retina* OR visual* OR vision*) N3 (disease* OR disorder* OR impair* OR loss) ) |
| S33 | TI ( glaucoma OR retinopath* OR cataract* ) OR AB ( glaucoma OR retinopath* OR cataract* ) OR KW ( glaucoma OR retinopath* OR cataract* ) |
| S34 | TI ( ( (liver OR hepati*) N3 (disease* OR disorder* OR syndrome* OR alcohol* OR fail* OR fatty) ) OR AB ( ( (liver OR hepati*) N3 (disease* OR disorder* OR syndrome* OR alcohol* OR fail* OR fatty) ) OR KW ( ( (liver OR hepati*) N3 (disease* OR disorder* OR syndrome* OR alcohol* OR fail* OR fatty) ) |
| S35 | TI ( hepatitis OR cirrho* ) OR AB ( hepatitis OR cirrho* ) OR KW ( hepatitis OR cirrho* ) |
| S36 | TI ( (healthy OR unhealthy OR sedentary OR inactiv* OR activ* OR poor) N3 (life style OR lifestyle OR diet* OR nutrition* OR eating OR behav*) ) OR AB ( (healthy OR unhealthy OR sedentary OR inactiv* OR activ* OR poor) N3 (life style OR lifestyle OR diet* OR nutrition* OR eating OR behav*) ) OR KW ( (healthy OR unhealthy OR sedentary OR inactiv* OR activ* OR poor) N3 (life style OR lifestyle OR diet* OR nutrition* OR eating OR behav*) ) |
| S37 | TI ( "physical activit*" OR exerc* ) OR AB ( "physical activit*" OR exerc* ) OR KW ( "physical activit*" OR exerc* ) |
| S38 | TI ( alcohol* OR drinking ) OR AB ( alcohol* OR drinking ) OR KW ( alcohol* OR drinking ) |
| S39 | TI ( (drug* OR substance* OR prescription*) N3 (abus* OR addiction* OR dependence) ) OR AB ( (drug* OR substance* OR prescription*) N3 (abus* OR addiction* OR dependence) ) OR KW ( (drug* OR substance* OR prescription*) N3 (abus* OR addiction* OR dependence) ) |
| S40 | TI petrol* N2 sniffing OR AB petrol* N2 sniffing OR KW petrol* N2 sniffing |
| S41 | TI ( (health* OR unhealthy OR risk*) N5 behavio* ) OR AB ( (health* OR unhealthy OR risk*) N5 behavio* ) OR KW ( (health* OR unhealthy OR risk*) N5 behavio* ) |
| S42 | TI ( tobacco OR smoking OR smoker* OR cigarette* ) OR AB ( tobacco OR smoking OR smoker* OR cigarette* ) OR KW ( tobacco OR smoking OR smoker* OR cigarette* ) |
| S43 | TI ( overweight OR "unhealthy weight" OR obese OR obesity OR BMI OR "body mass index" ) OR AB ( overweight OR "unhealthy weight" OR obese OR obesity OR BMI OR "body mass index" ) OR KW ( overweight OR "unhealthy weight" OR obese OR obesity OR BMI OR "body mass index" ) |
| S44 | (S8 OR S9 OR S10 OR S11 OR S12 OR S13 OR S14 OR S15 OR S16 OR S17 OR S18 OR S19 OR S20 OR S21 OR S22 OR S23 OR S24 OR S25 OR S26 OR S27 OR S28 OR S29 OR S30 OR S31 OR S32 OR S33 OR S34 OR S35 OR S36 OR S37 OR S38 OR S39 OR S40 OR S41 OR S42 OR S43) |
| S45 | TI ( "general practic*" OR "primary health" OR clinic* ) OR AB ( "general practic*" OR "primary health" OR clinic* ) OR KW ( "general practic*" OR "primary health" OR clinic* ) |
| S46 | TI ( communit* N3 (led OR based OR directed) ) OR AB ( communit* N3 (led OR based OR directed) ) OR KW ( communit* N3 (led OR based OR directed) ) |
| S47 | TI ( health N3 (planning OR service* OR education OR literacy) ) OR AB ( health N3 (planning OR service* OR education OR literacy) ) OR KW ( health N3 (planning OR service* OR education OR literacy) ) |
| S48 | TI ( (aborigin* OR indigenous*) N3 (co-op* OR coop* OR health OR service* OR organisation*) ) OR AB ( (aborigin* OR indigenous*) N3 (co-op* OR coop* OR health OR service* OR organisation*) ) OR KW ( (aborigin* OR indigenous*) N3 (co-op* OR coop* OR health OR service* OR organisation*) ) |
| S49 | TI ( ACCHOs OR AMS OR AHS OR ACCHS ) OR AB ( ACCHOs OR AMS OR AHS OR ACCHS ) OR KW ( ACCHOs OR AMS OR AHS OR ACCHS ) |
| S50 | TI ( intervention* OR project* OR program* OR strateg* OR model* OR initiative* OR promotion* ) OR AB ( intervention* OR project* OR program* OR strateg* OR model* OR initiative* OR promotion* ) OR KW ( intervention* OR project* OR program* OR strateg* OR model* OR initiative* OR promotion* ) |
| S51 | (S45 OR S46 OR S47 OR S48 OR S49 OR S50) |
| S52 | S6 AND S7 AND S44 AND S51 |
| S53 | TI child* OR infant* OR teen* OR youth* OR adolescen* OR pediatric* OR paediatric* |
| S54 | S52 NOT S53 |

# Database(s):Scopus

# Search Strategy:

((TITLE-ABS-KEY(Aborigin* OR "Torres Strait Islander" OR TSI OR Torres OR Strait OR "first nation*" OR "first people*" OR indigen*) AND TITLE-ABS-KEY(Australia* OR victoria* OR tasmania* OR queensland* OR "new south wales" OR "northern territory") AND TITLE-ABS-KEY("chronic disease*" OR "chronic condition*" OR "chronic illness*" OR "non-communicable disease*" OR "noncommunicable disease*" OR "non communicable disease*" OR comorbid* OR multimorbid* OR ((coronary OR heart OR cardiac* OR cardiovascula*) W/2 (syndrome* OR disease* OR event* OR occlusion* OR stenos* OR thrombo* OR attack*)) OR CHD OR CVD OR angina OR ((lung* OR pulmonary OR bronchi* OR airway OR respiratory) W/3 (disease* OR disorder*)) OR COPD* OR asthma* OR bronchiectasis OR emphysema OR ((endocrine OR hormon* OR thyroid OR adrenal OR pituitary OR parathyroid OR pancrea*) W/3 (disease* OR disorder* OR hyposecret* OR hypersecret*)) OR hypothyroidism OR hyperthyroidism OR hyperparathyroidism OR hypocyclemia OR Hypopituitarism OR cushing* OR addison* OR ((kidney OR renal OR urologic*) W/3 (disease* OR disorder* OR fail* OR insufficien*)) OR ESRF OR ESKF OR ESRD OR ESKD OR CKF OR CKD OR CRF OR CRD OR predialysis OR pre-dialysis OR nephroma* OR nephrit* OR cancer* OR neoplas* OR tumo* OR carcinoma* OR hodgkin* OR nonhodgkin* OR adenocarcinoma* OR leuk?emia* OR metasta* OR malignan* OR lymphoma* OR sarcoma* OR melanoma* OR myeloma* OR oncolog* OR ((musculoskeletal* OR bone OR tendon* OR joint* OR cartilage OR muscular OR rheumati*) W/3 (disease* OR disorder* OR impair*)) OR osteoarthr* OR Fibromyalgia OR Tendonitis OR "tunnel syndrom*" OR ((hip OR knee OR back OR spinal OR spine OR neck OR pelvis OR pelvic) W/3 (problem* OR degenerat* OR disorder* OR disease*)) OR (disc* W/2 (herniat* OR burst OR ruptur* OR bulg*)) OR ((nutrition* OR metabolic OR glucose) W/3 (disease* OR disorder* OR syndrome* OR imbalance*)) OR diabet* OR T2DM OR DM OR NIDDM OR "metabolic syndrome*" OR (glyc?emic W/3 control*) OR (insulin W/2 (sensitiv* OR resistan*)) OR "mental health" OR "mental* ill*" OR "mood disorder*" OR "affective disorder*" OR "regulation disorder*" OR depressive OR depression OR dysthymi* OR anxiety OR psychiatric OR pain* OR ((tooth OR teeth OR oral OR dental) W/2 (disorder* OR disease* OR abnormal*)) OR ((eye* OR optic* OR cornea* OR orbital OR retina* OR visual* OR vision*) W/3 (disease* OR disorder* OR impair* OR loss)) OR glaucoma OR retinopath* OR cataract* OR ((liver OR hepati*) W/3 (disease* OR disorder* OR syndrome* OR alcohol* OR fail* OR fatty)) OR hepatitis OR cirrho* OR ((healthy OR unhealthy OR sedentary OR inactiv* OR activ* OR poor) W/3 ("life style" OR lifestyle OR diet* OR nutrition* OR eating OR behav*)) OR "physical activit*" OR exerc* OR alcohol* OR drinking OR ((drug* OR substance* OR prescription*) W/3 (abus* OR addiction* OR dependence)) OR (petrol* W/2 sniffing) OR ((health* OR unhealthy OR risk*) W/5 behavio*) OR tobacco OR smoking OR smoker* OR cigarette* OR overweight OR "unhealthy weight" OR obese OR obesity OR BMI OR "body mass index") AND TITLE-ABS-KEY("general practic*" OR "primary health" OR clinic* OR (communit* W/3 (led OR based OR directed)) OR (health W/3 (planning OR service* OR education OR literacy*)) OR ((aborigin* OR indigenous*) W/3 (co-op* OR coop* OR health OR service* OR organisation*)) OR ACCHOs OR AMS OR AHS OR ACCHS OR intervention* OR project* OR program* OR strateg* OR model* OR initiative* OR promotion*)) AND NOT TITLE(child* OR infant* OR teen* OR youth* OR adolescen* OR paediatric* OR pediatric*))

Database(s); **Web of Science and Cochrane**

Search Strategy:

((Aborigin* OR "Torres Strait Islander" OR TSI OR Torres OR Strait OR "first nation*" OR "first people*" OR indigen*) AND (Australia* OR victoria* OR tasmania* OR queensland* OR "new south wales" OR "northern territory") AND ("chronic disease*" OR "chronic condition*" OR "chronic illness*" OR "non-communicable disease*" OR "noncommunicable disease*" OR "non communicable disease*" OR comorbid* OR multimorbid* OR ((coronary OR heart OR cardiac* OR cardiovascula*) NEAR/2 (syndrome* OR disease* OR event* OR occlusion* OR stenos* OR thrombo* OR attack*)) OR CHD OR CVD OR angina OR ((lung* OR pulmonary OR bronchi* OR airway OR respiratory) NEAR/3 (disease* OR disorder*)) OR COPD* OR asthma* OR bronchiectasis OR emphysema OR ((endocrine OR hormon* OR thyroid OR adrenal OR pituitary OR parathyroid OR pancrea*) NEAR/3 (disease* OR disorder* OR hyposecret* OR hypersecret*)) OR hypothyroidism OR hyperthyroidism OR hyperparathyroidism OR hypocyclemia OR Hypopituitarism OR cushing* OR addison* OR ((kidney OR renal OR urologic*) NEAR/3 (disease* OR disorder* OR fail* OR insufficien*)) OR ESRF OR ESKF OR ESRD OR ESKD OR CKF OR CKD OR CRF OR CRD OR predialysis OR pre-dialysis OR nephroma* OR nephrit* OR cancer* OR neoplas* OR tumo* OR carcinoma* OR hodgkin* OR nonhodgkin* OR adenocarcinoma* OR leuk?emia* OR metasta* OR malignan* OR lymphoma* OR sarcoma* OR melanoma* OR myeloma* OR oncolog* OR ((musculoskeletal* OR bone OR tendon* OR joint* OR cartilage OR muscular OR rheumati*) NEAR/3 (disease* OR disorder* OR impair*)) OR osteoarthr* OR Fibromyalgia OR Tendonitis OR "tunnel syndrom*" OR ((hip OR knee OR back OR spinal OR spine OR neck OR pelvis OR pelvic) NEAR/3 (problem* OR degenerat* OR disorder* OR disease*)) OR (disc* NEAR/2 (herniat* OR burst OR ruptur* OR bulg*)) OR ((nutrition* OR metabolic OR glucose) NEAR/3 (disease* OR disorder* OR syndrome* OR imbalance*)) OR diabet* OR T2DM OR DM OR NIDDM OR "metabolic syndrome*" OR (glyc?emic NEAR/3 control*) OR (insulin NEAR/2 (sensitiv* OR resistan*)) OR "mental health" OR "mental* ill*" OR "mood disorder*" OR "affective disorder*" OR "regulation disorder*" OR depressive OR depression OR dysthymi* OR anxiety OR psychiatric OR pain* OR ((tooth OR teeth OR oral OR dental) NEAR/2 (disorder* OR disease* OR abnormal*)) OR ((eye* OR optic* OR cornea* OR orbital OR retina* OR visual* OR vision*) NEAR/3 (disease* OR disorder* OR impair* OR loss)) OR glaucoma OR retinopath* OR cataract* OR ((liver OR hepati*) NEAR/3 (disease* OR disorder* OR syndrome* OR alcohol* OR fail* OR fatty)) OR hepatitis OR cirrho* OR ((healthy OR unhealthy OR sedentary OR inactiv* OR activ* OR poor) NEAR/3 ("life style" OR lifestyle OR diet* OR nutrition* OR eating OR behav*)) OR "physical activit*" OR exerc* OR alcohol* OR drinking OR ((drug* OR substance* OR prescription*) NEAR/3 (abus* OR addiction* OR dependence)) OR (petrol* NEAR/2 sniffing) OR ((health* OR unhealthy OR risk*) NEAR/5 behavio*) OR tobacco OR smoking OR smoker* OR cigarette* OR overweight OR "unhealthy weight" OR obese OR obesity OR BMI OR "body mass index") AND ("general practic*" OR "primary health" OR clinic* OR (communit* NEAR/3 (led OR based OR directed)) OR (health NEAR/3 (planning OR service* OR education OR literacy*)) OR ((aborigin* OR indigenous*) NEAR/3 (co-op* OR coop* OR health OR service* OR organisation*)) OR ACCHOs OR AMS OR AHS OR ACCHS OR intervention* OR project* OR program* OR strateg* OR model* OR initiative* OR promotion*)) NOT child* OR infant* OR teen* OR youth* OR adolescen* OR paediatric* OR pediatric*)

Database(s); **INFORMIT**

Search Strategy:

(((TI=(Aborigin* OR "Torres Strait Islander" OR TSI OR Torres OR Strait OR "first nation*" OR "first people*" OR indigen*) AND (Australia* OR victoria* OR tasmania* OR queensland* OR "new south wales" OR "northern territory") AND ("chronic disease*" OR "chronic condition*" OR "chronic illness*" OR "non-communicable disease*" OR "noncommunicable disease*" OR "non communicable disease*" OR comorbid* OR multimorbid* OR ((coronary OR heart OR cardiac* OR cardiovascula*) %2 syndrome*) OR ((coronary OR heart OR cardiac* OR cardiovascula*) %2 disease*) OR ((coronary OR heart OR cardiac* OR cardiovascula*) %2 event*) OR ((coronary OR heart OR cardiac* OR cardiovascula*) %2 occlusion*) OR ((coronary OR heart OR cardiac* OR cardiovascula*) %2 stenos*) OR ((coronary OR heart OR cardiac* OR cardiovascula*) %2 thrombo*) OR ((coronary OR heart OR cardiac* OR cardiovascula*) %2 attack*) OR CHD OR CVD OR angina OR ((lung* OR pulmonary OR bronchi* OR airway OR respiratory) %3 disease*) OR ((lung* OR pulmonary OR bronchi* OR airway OR respiratory) %3 disorder*) COPD* OR asthma* OR bronchiectasis OR emphysema OR ((endocrine OR hormon* OR thyroid OR adrenal OR pituitary OR parathyroid OR pancrea*) %3 disease*) OR ((endocrine OR hormon* OR thyroid OR adrenal OR pituitary OR parathyroid OR pancrea*) %3 disorder*) OR ((endocrine OR hormon* OR thyroid OR adrenal OR pituitary OR parathyroid OR pancrea*) %3 hyposecret*) OR ((endocrine OR hormon* OR thyroid OR adrenal OR pituitary OR parathyroid OR pancrea*) %3 hypersecret*) OR hypothyroidism OR hyperthyroidism OR hyperparathyroidism OR hypocyclemia OR Hypopituitarism OR cushing* OR addison* OR ((kidney OR renal OR urologic*) %3 disease*) OR ((kidney OR renal OR urologic*) %3 disorder*) OR ((kidney OR renal OR urologic*) %3 fail*) OR ((kidney OR renal OR urologic*) %3 insufficien*) OR ESRF OR ESKF OR ESRD OR ESKD OR CKF OR CKD OR CRF OR CRD OR predialysis OR pre-dialysis OR nephroma* OR nephrit* OR cancer* OR neoplas* OR tumo* OR carcinoma* OR hodgkin* OR nonhodgkin* OR adenocarcinoma* OR leukemia* OR leukaemia OR metasta* OR malignan* OR lymphoma* OR sarcoma* OR melanoma* OR myeloma* OR oncolog* OR ((musculoskeletal* OR bone OR tendon* OR joint* OR cartilage OR muscular OR rheumati*) %3 disease*) OR ((musculoskeletal* OR bone OR tendon* OR joint* OR cartilage OR muscular OR rheumati*) %3 disorder*) OR ((musculoskeletal* OR bone OR tendon* OR joint* OR cartilage OR muscular OR rheumati*) %3 impair*) OR osteoarthr* OR Fibromyalgia OR Tendonitis OR "tunnel syndrom*" OR ((hip OR knee OR back OR spinal OR spine OR neck OR pelvis OR pelvic) %3 problem*) OR ((hip OR knee OR back OR spinal OR spine OR neck OR pelvis OR pelvic) %3 degenerat*) OR ((hip OR knee OR back OR spinal OR spine OR neck OR pelvis OR pelvic) %3 disorder*) OR ((hip OR knee OR back OR spinal OR spine OR neck OR pelvis OR pelvic) %3 disease*) OR ((herniat* OR burst OR ruptur* OR bulg*) %2 disc*) OR ((nutrition* OR metabolic OR glucose) %3 disease*) OR ((nutrition* OR metabolic OR glucose) %3 disorder*) OR ((nutrition* OR metabolic OR glucose) %3 syndrome*) OR ((nutrition* OR metabolic OR glucose) %3 imbalance*) OR diabet* OR DM OR NIDDM OR "metabolic syndrome*" OR ((glycemic OR glycaemic) %3 control*) OR ((sensitiv* OR resistan*) %2 insulin) OR "mental health" OR "mental* ill*" OR "mood disorder*" OR "affective disorder*" OR "regulation disorder*" OR depressive OR depression OR dysthymi* OR anxiety OR psychiatric OR pain* OR ((tooth OR teeth OR oral OR dental) %2 disorder*) OR ((tooth OR teeth OR oral OR dental) %2 disease*) OR ((tooth OR teeth OR oral OR dental) %2 abnormal*) OR ((eye* OR optic* OR cornea* OR orbital OR retina* OR visual* OR vision*) %3 disease*) OR ((eye* OR optic* OR cornea* OR orbital OR retina* OR visual* OR vision*) %3 disorder*) OR ((eye* OR optic* OR cornea* OR orbital OR retina* OR visual* OR vision*) % impair*) OR ((eye* OR optic* OR cornea* OR orbital OR retina* OR visual* OR vision*) %3 loss) OR glaucoma OR retinopath* OR cataract* OR ((disease* OR disorder* OR syndrome* OR alcohol* OR fail* OR fatty) %2 liver) OR ((disease* OR disorder* OR syndrome* OR alcohol* OR fail* OR fatty) %2 hepati*) OR hepatitis OR cirrho* OR ((healthy OR unhealthy OR sedentary OR inactiv* OR activ* OR poor) %3 "life style") OR ((healthy OR unhealthy OR sedentary OR inactiv* OR activ* OR poor) %3 lifestyle) OR ((healthy OR unhealthy OR sedentary OR inactiv* OR activ* OR poor) %3 diet*) OR ((healthy OR unhealthy OR sedentary OR inactiv* OR activ* OR poor) %3 nutrition*) OR ((healthy OR unhealthy OR sedentary OR inactiv* OR activ* OR poor) %3 eating) OR ((healthy OR unhealthy OR sedentary OR inactiv* OR activ* OR poor) %3 behav*) OR "physical activit*" OR exerc* OR alcohol* OR drinking OR ((drug* OR substance* OR prescription*) %3 abus*) OR ((drug* OR substance* OR prescription*) %3 addiction*) OR ((drug* OR substance* OR prescription*) %3 dependence) OR (petrol* %2 sniffing) OR ((health* OR unhealthy OR risk*) %5 behavio*) OR tobacco OR smoking OR smoker* OR cigarette* OR overweight OR "unhealthy weight" OR obese OR obesity OR BMI OR "body mass index") AND ("general practic*" OR "primary health" OR clinic* OR ((led OR based OR directed) %3 communit*) OR ((planning OR service* OR education OR literacy*) %3 health) OR ((aborigin* OR indigenous*) %3 co-op*) OR ((aborigin* OR indigenous*) %3 coop*) OR ((aborigin* OR indigenous*) %3 health) OR ((aborigin* OR indigenous*) %3 service*) OR ((aborigin* OR indigenous*) %3 organisation*) OR ACCHOs OR AMS OR AHS OR ACCHS OR intervention* OR project* OR program* OR strateg* OR model* OR initiative* OR promotion*)) OR ((AB:Aborigin* OR AB:"Torres Strait Islander" OR AB:TSI OR AB:Torres OR AB:Strait OR AB:"first nation*" OR AB:"first people*" OR AB:indigen*) AND (AB:Australia* OR AB:victoria* OR AB:tasmania* OR AB:queensland* OR AB:"new south wales" OR AB:"northern territory") AND (AB:"chronic disease*" OR AB:"chronic condition*" OR AB:"chronic illness*" OR AB:"non-communicable disease*" OR AB:"noncommunicable disease*" OR AB:"non communicable disease*" OR AB:comorbid* OR AB:multimorbid* OR ((AB:coronary OR AB:heart OR AB:cardiac* OR AB:cardiovascula*)%2 AB:syndrome*) OR ((AB:coronary OR AB:heart OR AB:cardiac* OR AB:cardiovascula*)%2 AB:disease*) OR ((AB:coronary OR AB:heart OR AB:cardiac* OR AB:cardiovascula*)%2 AB:event*) OR ((AB:coronary OR AB:heart OR AB:cardiac* OR AB:cardiovascula*)%2 AB:occlusion*) OR ((AB:coronary OR AB:heart OR AB:cardiac* OR AB:cardiovascula*)%2 AB:stenos*) OR ((AB:coronary OR AB:heart OR AB:cardiac* OR AB:cardiovascula*)%2 AB:thrombo*) OR ((AB:coronary OR AB:heart OR AB:cardiac* OR AB:cardiovascula*)%2 AB:attack*) OR AB:CHD OR AB:CVD OR AB:angina OR ((AB:lung* OR AB:pulmonary OR AB:bronchi* OR AB:airway OR AB:respiratory)%3 AB:disease*) OR ((AB:lung* OR AB:pulmonary OR AB:bronchi* OR AB:airway OR AB:respiratory)%3 AB:disorder*)AB:COPD* OR AB:asthma* OR AB:bronchiectasis OR AB:emphysema OR ((AB:endocrine OR AB:hormon* OR AB:thyroid OR AB:adrenal OR AB:pituitary OR AB:parathyroid OR AB:pancrea*)%3 AB:disease*) OR ((AB:endocrine OR AB:hormon* OR AB:thyroid OR AB:adrenal OR AB:pituitary OR AB:parathyroid OR AB:pancrea*)%3 AB:disorder*) OR ((AB:endocrine OR AB:hormon* OR AB:thyroid OR AB:adrenal OR AB:pituitary OR AB:parathyroid OR AB:pancrea*)%3 AB:hyposecret*) OR ((AB:endocrine OR AB:hormon* OR AB:thyroid OR AB:adrenal OR AB:pituitary OR AB:parathyroid OR AB:pancrea*)%3 AB:hypersecret*) OR AB:hypothyroidism OR AB:hyperthyroidism OR AB:hyperparathyroidism OR AB:hypocyclemia OR AB:Hypopituitarism OR AB:cushing* OR AB:addison* OR ((AB:kidney OR AB:renal OR AB:urologic*)%3 AB:disease*) OR ((AB:kidney OR AB:renal OR AB:urologic*)%3 AB:disorder*) OR ((AB:kidney OR AB:renal OR AB:urologic*)%3 AB:fail*) OR ((AB:kidney OR AB:renal OR AB:urologic*)%3 AB:insufficien*) OR AB:ESRF OR AB:ESKF OR AB:ESRD OR AB:ESKD OR AB:CKF OR AB:CKD OR AB:CRF OR AB:CRD OR AB:predialysis OR AB:pre-dialysis OR AB:nephroma* OR AB:nephrit* OR AB:cancer* OR AB:neoplas* OR AB:tumo* OR AB:carcinoma* OR AB:hodgkin* OR AB:nonhodgkin* OR AB:adenocarcinoma* OR AB:leukemia* OR AB:leukaemia OR AB:metasta* OR AB:malignan* OR AB:lymphoma* OR AB:sarcoma* OR AB:melanoma* OR AB:myeloma* OR AB:oncolog* OR ((AB:musculoskeletal* OR AB:bone OR AB:tendon* OR AB:joint* OR AB:cartilage OR AB:muscular OR AB:rheumati*)%3 AB:disease*) OR ((AB:musculoskeletal* OR AB:bone OR AB:tendon* OR AB:joint* OR AB:cartilage OR AB:muscular OR AB:rheumati*)%3 AB:disorder*) OR ((AB:musculoskeletal* OR AB:bone OR AB:tendon* OR AB:joint* OR AB:cartilage OR AB:muscular OR AB:rheumati*)%3 AB:impair*) OR AB:osteoarthr* OR AB:Fibromyalgia OR AB:Tendonitis OR AB:"tunnel syndrom*" OR ((AB:hip OR AB:knee OR AB:back OR AB:spinal OR AB:spine OR AB:neck OR AB:pelvis OR AB:pelvic)%3 AB:problem*) OR ((AB:hip OR AB:knee OR AB:back OR AB:spinal OR AB:spine OR AB:neck OR AB:pelvis OR AB:pelvic)%3 AB:degenerat*) OR ((AB:hip OR AB:knee OR AB:back OR AB:spinal OR AB:spine OR AB:neck OR AB:pelvis OR AB:pelvic)%3 AB:disorder*) OR ((AB:hip OR AB:knee OR AB:back OR AB:spinal OR AB:spine OR AB:neck OR AB:pelvis OR AB:pelvic)%3 AB:disease*) OR ((AB:herniat* OR AB:burst OR AB:ruptur* OR AB:bulg*)%2 AB:disc*) OR ((AB:nutrition* OR AB:metabolic OR AB:glucose)%3 AB:disease*) OR ((AB:nutrition* OR AB:metabolic OR AB:glucose)%3 AB:disorder*) OR ((AB:nutrition* OR AB:metabolic OR AB:glucose)%3 AB:syndrome*) OR ((AB:nutrition* OR AB:metabolic OR AB:glucose)%3 AB:imbalance*) OR AB:diabet* OR AB:DM OR AB:NIDDM OR AB:"metabolic syndrome*" OR ((AB:glycemic OR AB:glycaemic)%3 AB:control*) OR ((AB:sensitiv* OR AB:resistan*)%2 AB:insulin) OR AB:"mental health" OR AB:"mental* ill*" OR AB:"mood disorder*" OR AB:"affective disorder*" OR AB:"regulation disorder*" OR AB:depressive OR AB:depression OR AB:dysthymi* OR AB:anxiety OR AB:psychiatric OR AB:pain* OR ((AB:tooth OR AB:teeth OR AB:oral OR AB:dental)%2 AB:disorder*) OR ((AB:tooth OR AB:teeth OR AB:oral OR AB:dental)%2 AB:disease*) OR ((AB:tooth OR AB:teeth OR AB:oral OR AB:dental)%2 AB:abnormal*) OR ((AB:eye* OR AB:optic* OR AB:cornea* OR AB:orbital OR AB:retina* OR AB:visual* OR AB:vision*)%3 AB:disease*) OR ((AB:eye* OR AB:optic* OR AB:cornea* OR AB:orbital OR AB:retina* OR AB:visual* OR AB:vision*)%3 AB:disorder*) OR ((AB:eye* OR AB:optic* OR AB:cornea* OR AB:orbital OR AB:retina* OR AB:visual* OR AB:vision*)% AB:impair*) OR ((AB:eye* OR AB:optic* OR AB:cornea* OR AB:orbital OR AB:retina* OR AB:visual* OR AB:vision*)%3 AB:loss) OR AB:glaucoma OR AB:retinopath* OR AB:cataract* OR ((AB:disease* OR AB:disorder* OR AB:syndrome* OR AB:alcohol* OR AB:fail* OR AB:fatty)%2 AB:liver) OR ((AB:disease* OR AB:disorder* OR AB:syndrome* OR AB:alcohol* OR AB:fail* OR AB:fatty)%2 AB:hepati*) OR AB:hepatitis OR AB:cirrho* OR ((AB:healthy OR AB:unhealthy OR AB:sedentary OR AB:inactiv* OR AB:activ* OR AB:poor)%3 AB:"life style") OR ((AB:healthy OR AB:unhealthy OR AB:sedentary OR AB:inactiv* OR AB:activ* OR AB:poor)%3 AB:lifestyle) OR ((AB:healthy OR AB:unhealthy OR AB:sedentary OR AB:inactiv* OR AB:activ* OR AB:poor)%3 AB:diet*) OR ((AB:healthy OR AB:unhealthy OR AB:sedentary OR AB:inactiv* OR AB:activ* OR AB:poor)%3 AB:nutrition*) OR ((AB:healthy OR AB:unhealthy OR AB:sedentary OR AB:inactiv* OR AB:activ* OR AB:poor)%3 AB:eating) OR ((AB:healthy OR AB:unhealthy OR AB:sedentary OR AB:inactiv* OR AB:activ* OR AB:poor)%3 AB:behav*) OR AB:"physical activit*" OR AB:exerc* OR AB:alcohol* OR AB:drinking OR ((AB:drug* OR AB:substance* OR AB:prescription*)%3 AB:abus*) OR ((AB:drug* OR AB:substance* OR AB:prescription*)%3 AB:addiction*) OR ((AB:drug* OR AB:substance* OR AB:prescription*)%3 AB:dependence) OR (AB:petrol* %2 AB:sniffing) OR ((AB:health* OR AB:unhealthy OR AB:risk*)%5 AB:behavio*) OR AB:tobacco OR AB:smoking OR AB:smoker* OR AB:cigarette* OR AB:overweight OR AB:"unhealthy weight" OR AB:obese OR AB:obesity OR AB:BMI OR AB:"body mass index") AND (AB:"general practic*" OR AB:"primary health" OR AB:clinic* OR ((AB:led OR AB:based OR AB:directed)%3 AB:communit*) OR ((AB:planning OR AB:service* OR AB:education OR AB:literacy*)%3 AB:health) OR ((AB:aborigin* OR AB:indigenous*)%3 AB:co-op*) OR ((AB:aborigin* OR AB:indigenous*)%3 AB:coop*) OR ((AB:aborigin* OR AB:indigenous*)%3 AB:health) OR ((AB:aborigin* OR AB:indigenous*)%3 AB:service*) OR ((AB:aborigin* OR AB:indigenous*)%3 AB:organisation*) OR AB:ACCHOs OR AB:AMS OR AB:AHS OR AB:ACCHS OR AB:intervention* OR AB:project* OR AB:program* OR AB:strateg* OR AB:model* OR AB:initiative* OR AB:promotion*))) NOT (TI:child* OR TI:infant* OR TI:teen* OR TI:youth* OR TI:adolescen* OR TI:paediatric* OR TI:pediatric*))

Database(s); **PMC**

Search Strategy:

(Aborigin*[tiab] OR "Torres Strait Islander"[tiab] OR TSI[tiab] OR Torres[tiab] OR Strait[tiab] OR "first nation*"[tiab] OR "first people*"[tiab] OR indigen*[tiab]) AND (Australia*[tiab] OR victoria*[tiab] OR tasmania*[tiab] OR queensland*[tiab] OR "new south wales"[tiab] OR "northern territory"[tiab]) AND ("chronic disease*"[tiab] OR "chronic condition*"[tiab] OR "chronic illness*"[tiab] OR "non-communicable disease*"[tiab] OR "noncommunicable disease*"[tiab] OR "non communicable disease*"[tiab] OR comorbid*[tiab] OR multimorbid*[tiab] OR ((coronary[tiab] OR heart[tiab] OR cardiac*[tiab] OR cardiovascula*[tiab]) AND (syndrome*[tiab] OR disease*[tiab] OR event*[tiab] OR occlusion*[tiab] OR stenos*[tiab] OR thrombo*[tiab] OR attack*[tiab]))[tiab] OR CHD[tiab] OR CVD[tiab] OR angina[tiab] OR ((lung*[tiab] OR pulmonary[tiab] OR bronchi*[tiab] OR airway[tiab] OR respiratory[tiab]) AND (disease*[tiab] OR disorder*[tiab])) OR COPD*[tiab] OR asthma*[tiab] OR bronchiectasis[tiab] OR emphysema[tiab] OR ((endocrine[tiab] OR hormon*[tiab] OR thyroid[tiab] OR adrenal[tiab] OR pituitary[tiab] OR parathyroid[tiab] OR pancrea*[tiab]) AND (disease*[tiab] OR disorder*[tiab] OR hyposecret*[tiab] OR hypersecret*[tiab])) OR hypothyroidism[tiab] OR hyperthyroidism[tiab] OR hyperparathyroidism[tiab] OR hypocyclemia[tiab] OR Hypopituitarism[tiab] OR cushing*[tiab] OR addison*[tiab] OR ((kidney[tiab] OR renal[tiab] OR urologic*[tiab]) AND (disease*[tiab] OR disorder*[tiab] OR fail*[tiab] OR insufficien*[tiab])) OR ESRF[tiab] OR ESKF[tiab] OR ESRD[tiab] OR ESKD[tiab] OR CKF[tiab] OR CKD[tiab] OR CRF[tiab] OR CRD[tiab] OR predialysis[tiab] OR pre-dialysis[tiab] OR nephroma*[tiab] OR nephrit*[tiab] OR cancer*[tiab] OR neoplas*[tiab] OR tumo*[tiab] OR carcinoma*[tiab] OR hodgkin*[tiab] OR nonhodgkin*[tiab] OR adenocarcinoma*[tiab] OR leuk?emia*[tiab] OR metasta*[tiab] OR malignan*[tiab] OR lymphoma*[tiab] OR sarcoma*[tiab] OR melanoma*[tiab] OR myeloma*[tiab] OR oncolog*[tiab] OR ((musculoskeletal*[tiab] OR bone[tiab] OR tendon*[tiab] OR joint*[tiab] OR cartilage[tiab] OR muscular[tiab] OR rheumati*[tiab]) AND (disease*[tiab] OR disorder*[tiab] OR impair*))[tiab] OR osteoarthr*[tiab] OR Fibromyalgia[tiab] OR Tendonitis[tiab] OR "tunnel syndrom*"[tiab] OR ((hip[tiab] OR knee[tiab] OR back[tiab] OR spinal[tiab] OR spine[tiab] OR neck[tiab] OR pelvis[tiab] OR pelvic[tiab]) AND (problem*[tiab] OR degenerat*[tiab] OR disorder*[tiab] OR disease*[tiab])) OR (disc* AND (herniat*[tiab] OR burst[tiab] OR ruptur*[tiab] OR bulg*[tiab])) OR ((nutrition*[tiab] OR metabolic[tiab] OR glucose[tiab]) AND (disease*[tiab] OR disorder*[tiab] OR syndrome*[tiab] OR imbalance*[tiab])) OR diabet*[tiab] OR T2DM[tiab] OR DM[tiab] OR NIDDM[tiab] OR "metabolic syndrome*"[tiab] OR (glyc?emic[tiab] AND control*[tiab]) OR (insulin[tiab] AND (sensitiv*[tiab] OR resistan*[tiab])) OR "mental health"[tiab] OR "mental* ill*"[tiab] OR "mood disorder*"[tiab] OR "affective disorder*"[tiab] OR "regulation disorder*"[tiab] OR depressive[tiab] OR depression[tiab] OR dysthymi*[tiab] OR anxiety[tiab] OR psychiatric[tiab] OR pain*[tiab] OR ((tooth[tiab] OR teeth[tiab] OR oral[tiab] OR dental[tiab]) AND (disorder*[tiab] OR disease*[tiab] OR abnormal*[tiab]))[tiab] OR ((eye*[tiab] OR optic*[tiab] OR cornea*[tiab] OR orbital[tiab] OR retina*[tiab] OR visual*[tiab] OR vision*[tiab]) AND (disease*[tiab] OR disorder*[tiab] OR impair*[tiab] OR loss[tiab])) OR glaucoma[tiab] OR retinopath*[tiab] OR cataract*[tiab] OR ((liver[tiab] OR hepati*[tiab]) AND (disease*[tiab] OR disorder*[tiab] OR syndrome*[tiab] OR alcohol*[tiab] OR fail*[tiab] OR fatty[tiab])) OR hepatitis[tiab] OR cirrho*[tiab] OR ((healthy[tiab] OR unhealthy[tiab] OR sedentary[tiab] OR inactiv*[tiab] OR activ*[tiab] OR poor[tiab]) AND ("life style"[tiab] OR lifestyle[tiab] OR diet*[tiab] OR nutrition*[tiab] OR eating[tiab] OR behav*[tiab])) OR "physical activit*"[tiab] OR exerc*[tiab] OR alcohol*[tiab] OR drinking[tiab] OR ((drug*[tiab] OR substance*[tiab] OR prescription*[tiab]) AND (abus*[tiab] OR addiction*[tiab] OR dependence[tiab])) OR (petrol*[tiab] AND sniffing[tiab]) OR ((health*[tiab] OR unhealthy[tiab] OR risk*[tiab]) AND behavio*[tiab]) OR tobacco[tiab] OR smoking[tiab] OR smoker*[tiab] OR cigarette*[tiab] OR overweight[tiab] OR "unhealthy weight"[tiab] OR obese[tiab] OR obesity[tiab] OR BMI[tiab] OR "body mass index"[tiab]) AND ("general practic*"[tiab] OR "primary health"[tiab] OR clinic*[tiab] OR (communit*[tiab] AND (led[tiab] OR based[tiab] OR directed[tiab])) OR (health[tiab] AND (planning[tiab] OR service*[tiab] OR education[tiab] OR literacy*[tiab])) OR ((aborigin*[tiab] OR indigenous*[tiab]) AND (co-op*[tiab] OR coop*[tiab] OR health[tiab] OR service*[tiab] OR organisation*[tiab])) OR ACCHOs[tiab] OR AMS[tiab] OR AHS[tiab] OR ACCHS[tiab] OR intervention*[tiab] OR project*[tiab] OR program*[tiab] OR strateg*[tiab] OR model*[tiab] OR initiative*[tiab] OR promotion*[tiab])
