# Supplementary material for: Geographical analysis of evaluated chronic disease programs for Aboriginal and Torres Strait Islander people in the Australian primary health care setting: a systematic scoping review
Source: BMC Public Health. 2019 Aug 14;19:1115. doi: 10.1186/s12889-019-7463-0 (PMC6694647; doi:10.1186/s12889-019-7463-0)
Supplement: Supplementary file 3 — Excluded Studies. This file contains a table of excluded studies and reasons for exclusion. (DOCX 68 kb) [file 12889_2019_7463_MOESM3_ESM.docx]

**Additional File 3. Excluded studies**

| **Reference** | **Reason for exclusion** |
| --- | --- |
| Bridge P. Ord Valley Aboriginal Health Service's fetal alcohol spectrum disorders program: Big steps, solid outcome. Australian Indigenous HealthBulletin. 2011;11(4):1-8. | Endpoint not a chronic disease or included chronic disease |
| Burrows A, Allen B, Gorton S. Evaluation of the Bumps to Babes and Beyond program. Queen Elizabeth Centre. 2014 http://www.qec.org.au/sites/default/files/news_pdf/Evaluation%20of%20the%20Bumps%20to%20Babes%20and%20Beyond%20Program.pdf. Accessed 10 Dec 2018 | Endpoint not a chronic disease or included chronic disease |
| Stewart T, McDonald R, Currie B. Acute rheumatic fever: adherence to secondary prophylaxis and follow up of Indigenous patients in the Katherine region of the Northern Territory. Aust J Rural Health. 2007;15(4):234-40. | Endpoint not a chronic disease or included chronic disease |
| Wearne B, Chesters J, Whyte S. Funding sources and consequences: the subverting of an Indigenous community outreach program. Rural Remote Health. 2006;6(3):542. | Endpoint not a chronic disease or included chronic disease |
| Australian Government Department of Health. Examination of Australian Government Indigenous ear and hearing initiatives. Australian Government Department of Health. 2018  http://www.health.gov.au/internet/main/publishing.nsf/Content/examination-of-australian-government-indigenous-ear-and-hearing-health-initiatives. Accessed 10 Dec 2018 | Evaluation of a government policy |
| Bailie J, Schierhout G, Kelaher M, Laycock A, Percival N et al. Follow-up of Indigenous-specific health assessments - a socioecological analysis. Med J Aust. 2014:200(11);653-657. | Evaluation of a government policy |
| Bailie J, Schierhout G, Laycock A, Kelaher M, Percival N et al. Determinants of access to chronic illness care: a mixed-methods evaluation of a national multifaceted chronic disease package for Indigenous Australians. BMJ Open. 2015;5(11)11. | Evaluation of a government policy |
| Bassilios B, Nicholas A, Reifels L, King K, Fletcher J et al. Achievements of the Australian Access to Allied Psychological Services (ATAPS) program: summarising (almost) a decade of key evaluation data. Int J Ment Health Syst. 2016;10(61):1-13. | Evaluation of a government policy |
| Calma T. Tackling Indigenous smoking in rural and remote Australia: progress and possibilities. In: Proceedings of the 13^th^ National Rural Health Conference; 2015 May 24-27, Northern Territory. National Rural Health Conference. http://www.ruralhealth.org.au/13nrhc/images/abs_Calma%2C%20Tom.pdf. Accessed 11 Dec 2018 | Evaluation of a government policy |
| D'Abbs P, Schmidt B, Senior K. Implementing a chronic disease strategy in two remote Indigenous Australian settings: a multi-method pilot evaluation. Aust J Rural Health. 2008;16(2):67-74 | Evaluation of a government policy |
| Australian Government Department of Health. Indigenous Australians' Health Programme (chronic disease activities). 2015 http://www.health.gov.au/internet/main/publishing.nsf/Content/IAHP-chronic-disease. Accessed 10 Dec 2018 | Evaluation of a government policy |
| Cultural & Indigenous Research Centre Australia. Tackling Indigenous Smoking Program evaluation: preliminary evaluation report. Australian Government Department of Health. 2017. https://healthinfonet.ecu.edu.au/uploads/resources/33380_33380.pdf. Accessed 10 Dec 2018 | Evaluation of a government policy |
| Australian Government Department of Health. Tackling Indigenous Smoking and Health Lifestyle Programme Review. Australian Government Department of Health. 2015. http://www.health.gov.au/internet/main/publishing.nsf/Content/indigenous-tis-hlp-review. Accessed 11 Dec 2018 | Evaluation of a government policy |
| Upton P, Davey R, Evans M, Mikhailovich K, Simpson L et al. Tackling Indigenous Smoking and Healthy Lifestyle Programme review: stakeholder consultation. University of Canberra. 2014. http://www.health.gov.au/internet/main/publishing.nsf/Content/904B8752C99678A1CA257EA00026976F/$File/TIS-and-Healthy-Lifestyle-Programme-Review_Stakeholder-Consultation_report2.pdf. Accessed 12 Dec 2018. | Evaluation of a government policy |
| Upton P, Davey R, Evans M, Mikhailovich K, Simpson L et al. Tackling Indigenous Smoking and Healthy Lifestyle Programme review: executive summary. University of Canberra. 2014. <http://www.health.gov.au/internet/main/publishing.nsf/Content/904B8752C99678A1CA257EA00026976F/$File/TIS-and-Healthy-lifestyle-Programme_Executive%20Summary.pdf>. Accessed 12 Dec 2018. | Evaluation of a government policy |
| Upton P, Davey R, Evans M, Mikhailovich K, Simpson L et al. Tackling Indigenous Smoking and Healthy Lifestyle Programme review: a multi-criteria decision analysis. University of Canberra. 2014. http://www.health.gov.au/internet/main/publishing.nsf/Content/904B8752C99678A1CA257EA00026976F/$File/TIS-and-Healthy-Lifestyle-Programme-Review_A-Multi-criteria-Decision-Analysis.pdf. Accessed 12 Dec 2018. | Evaluation of a government policy |
| Wilczynski A, Reed-Gilbert K, Milward K, Tayler B, Fear J et al. Evaluation of the Bringing Them Home and Indigenous Mental Health Programs: final report. Commonwealth of Australia. 2007. http://webarchive.nla.gov.au/gov/20140212001425/http://www.health.gov.au/internet/main/publishing.nsf/Content/health-oatsih-pubs-bth-eval. Accessed 13 Dec 2018 | Evaluation of a government policy |
| Dobson L, Tan I, Wu X, Muir J, Turner A. Lions outback vision van-3 month preliminary audit of service to rural and remote Western Australia. Clin. & Experiment. Opthalmol. 2016;44:98. | Evaluation of a specialist outreach clinic |
| Moynihan V, O’Halloran R, Frost E, Turner, A. Diabetic retinal screening in western australia: An audit of the lions outback vision screening program. Clin. & Experiment. Opthalmol. 2014;42:90 | Evaluation of a specialist outreach clinic |
| Moynihan V, Turner A. The case for regional diabetic eye health coordinators: A kimberley case study. Clin & Experiment. Opthalmol. 2015;43:90. | Evaluation of a specialist outreach clinic |
| Moynihan V, Turner A. Coordination of diabetic retinopathy screening in the Kimberley region of Western Australia. Aust J Rural Health. 2017;25(2):110-115. | Evaluation of a specialist outreach clinic |
| O'Halloran RA, Turner AW. Evaluating the impact of optical coherence tomography in diabetic retinopathy screening for an aboriginal population. Clin. & Experiment. Opthalmol. 2016;44:38. | Evaluation of a specialist outreach clinic |
| O'Halloran R, Turner A. Evaluating the impact of optical coherence tomography in diabetic retinopathy screening for an aboriginal population. Clin. & Experiment. Opthalmol. 2018;46(2):116-121. | Evaluation of a specialist outreach clinic |
| Shephard MD, Allen G, Paizis K, Barbara J, Batterham M et al. Results of an Aboriginal community-based renal disease management program incorporating point of care testing for urine albumin: creatinine ratio. Rural Remote Health. 2006;6(4):591. | Evaluation of a specialist outreach clinic |
| Aliakbari J, Latimore R, Polson C, Ross-Kelly M, Hannan-Jones M. A partnership approach to delivering health education in remote Indigenous communities. In: Proceedings of the 12^th^ National Rural Health Conference; 2013 April 7-10, Adelaide. National Rural Health Conference. http://www.ruralhealth.org.au/12nrhc/wp-content/uploads/2013/06/Latimore-Rachel_Polson-Cara_ppr.pdf. Accessed 11 Dec 2018. | Involves children under 18 years or young adults sample not stratified |
| Day A, Nakata M, Miller K. Programs to improve the social and emotional wellbeing of Aboriginal and Torres Strait Islander communities. Australian Social Work. 2016;69(3):373-380. | Involves children under 18 years or young adults sample not stratified |
| Eley R, Norman M. Music therapy to manage asthma symptoms in young Indigenous people in urban setting. Aborig Isl Health Worker J. 2010;34(3):20-22. | Involves children under 18 years or young adults sample not stratified |
| Eley R. The potential effects of the didgeridoo as an Indigenous intervention for Australian Aborigines: a post analysis. Music Med. 2013;5(2):84-92. | Involves children under 18 years or young adults sample not stratified |
| Eley R, Gorman D. Didgeridoo Playing and Singing to Support Asthma Management in Aboriginal Australians. J Rural Health. 2010;26(1):100-104. | Involves children under 18 years or young adults sample not stratified |
| Eley G, Gorman D, Gately J. Didgeridoos, Songs and Boomerangs for Asthma Management. Health Promot J Austr. 2010;21(1):39-44. | Involves children under 18 years or young adults sample not stratified |
| Lowitja Institute. Evaluation of anaemia management in three remote Aboriginal communities in Katherine East Region, Northern Territory. Lowitja Institute. 2016. https://www.lowitja.org.au/remote-anaemia-management-evaluation. Accessed 12 Dec 2018. | Involves children under 18 years or young adults sample not stratified |
| Hayward C, Beyondblue, Edith Cowan University, Kurongkurl Katitjin Centre for Indigenous Australian Education and Research. Summary report on the evaluation of Indigenous Hip Hop Projects by beyondblue. Hawthorn West: Beyondblue; 2009. | Involves children under 18 years or young adults sample not stratified |
| Kurongkurl Katitjin, Centre for Indigenous Australian Education and Research, Beyondblue, Edith Cowan University. Evaluation of Indigenous Hip Hop Projects. Beyondblue. 2009. <https://www.beyondblue.org.au/docs/default-source/research-project-files/bw0171.pdf?sfvrsn=1f48b2e9_2>. Accessed 14 Dec 2018. | Involves children under 18 years or young adults sample not stratified |
| Aitken L, Anderson I, Atkinson V, Best J, Briggs P. Erratum: A collaborative cardiovascular health program for Aboriginal and Torres Strait Islander people in the Goulburn-Murray region: Development and risk factor screening at indigenous community organisations. Aust J Prim Health. 2007;13(2):129. | Limited Indigenous participation or no stratification of results |
| Büsst CJ, O'Donnell M, Timoshanko A. The life! Program: Lessons from 8 years of real-world state-wide prevention. Endocr Pract. 2016;22:8-9. | Limited Indigenous participation or no stratification of results |
| Cardona-Morrell M. Evaluation of a Community-wide Diabetes Prevention Program. University of Sydney. 2012. https://ses.library.usyd.edu.au/handle/2123/8349. Accessed 15 Dec 2018. | Limited Indigenous participation or no stratification of results |
| Gorham G, Chatfield M, Lawton P, Cass A. Analysis of longitudinal clinical data to evaluate changes in the screening for and management of CKD in select NT primary health services. Nephrology. 2017;22:65 | Limited Indigenous participation or no stratification of results |
| Grebely J, Alavi M, Micallef M, Dunlop A, Balcomb A et al. Treatment for hepatitis C virus infection among people who inject drugs attending opioid substitution treatment and community health clinics: the ETHOS Study. Addiction. 2016;111(2):311-9. | Limited Indigenous participation or no stratification of results |
| Jorm LR, Walter SR, Lujic S, Byles JE, Kendig HL. Home and community care services: a major opportunity for preventative health care. BMC Geriatrics. 2010;10:26. | Limited Indigenous participation or no stratification of results |
| Laws RA, Vita P, Venugopal K, Rissel C, Davies D et al. Factors influencing participant enrolment in a diabetes prevention program in general practice: lessons from the Sydney diabetes prevention program. BMC Public Health. 2012;12:822. | Limited Indigenous participation or no stratification of results |
| Liu H, Massi L, Laba T, Jan S. Understanding adherence to a cardiovascular polypill strategy- a process evaluation of a pragmatic clinical trial. Glob Heart. 2014;9(1):118. | Limited Indigenous participation or no stratification of results |
| Liu H, Massi L, Laba TL, Peiris D, Usherwood T et al. Patients' and providers' perspectives of a polypill strategy to improve cardiovascular prevention in Australian Primary Health Care. Circulation. 2015;8(3):301-308. | Limited Indigenous participation or no stratification of results |
| Lloyd AR, Clegg J, Lange J, Stevenson A, Post J et al. Safety and Effectiveness of a Nurse-Led Outreach Program for Assessment and Treatement of Chronic Hepatitis C in the Custodial Setting. Clin Infect Dis. 2013;56(8):1078-1084. | Limited Indigenous participation or no stratification of results |
| Ludlow M, Mathew T, Whalen T. Key to good health: Assessing the effectiveness of community screening for chronic kidney disease. Nephrology. 2013;18:33. | Limited Indigenous participation or no stratification of results |
| Scrace M, Margolis SA. The Royal Flying Doctor Service primary care skin cancer clinic: a pilot program for remote Australia. Rural Remote Health. 2009;9(1):1048. | Limited Indigenous participation or no stratification of results |
| Reifels L, Bassilios B, King K, Fletcher J, Blashki G et al. Innovations in primary mental healthcare. Aust Health Rev. 2013;37(3):312-317. | Limited Indigenous participation or no stratification of results |
| Shakeshaft A. Improving substance abuse treatment outcomes for Indigenous and non-Indigenous Australians in rural, community-based health settings. Drug and alcohol research connections. 2016. http://connections.edu.au/researchfocus/improving-substance-abuse-treatment-outcomes-indigenous-and-non-indigenous-australians. Accessed 16 Dec 2018. | Limited Indigenous participation or no stratification of results |
| Shakeshaft A, Doran C, Petrie D, Breen C, Harvard A et al. The effectiveness of community action in reducing risky alcohol consumption and harm: a cluster randomised controlled trial. PLoS Med. 2014;11(3):14. | Limited Indigenous participation or no stratification of results |
| Tall JA, Brew BK, Saurman E, Jones T. Implementing an anti-smoking program in rural-remote communities: challenges and strategies. Rural Remote Health. 2015;15(4):15. | Limited Indigenous participation or no stratification of results |
| Titov N, Dear B, Staples L, Bennett-Levy J, Klein B et al. The first 30 months of the MindSpot Clinic: Evaluation of a national e-mental health service against project objectives. Aust N Z J Psychiatry. 2017;51(12):1227-1239. | Limited Indigenous participation or no stratification of results |
| Sun J, Buys N. Using community singing as a culturally appropriate approach to promote wellbeing in people with depression. J Alt Med Res. 2013;5(2):111-117. | Manuscript unavailable |
| Warnock J. Report: Mount Isa's Indigenous Diabetic Foot Project. Services for Australian Rural and Remote Allied Health Inc. 2006. | Manuscript unavailable |
| Western Australian General Practice Network. WA Indigenous healthy lifestyle project. Western Australian General Practice Network. 2009 | Manuscript unavailable |
| Martini A, Javanparast S, Ward P, Baratiny G, Gill T et al. Colorectal cancer screening in rural and remote areas: analysis of the National Bowel Cancer Screening Program data for South Australia. Rural Remote Health. 2011:11(2);1648. | National screening or vaccination program |
| Australian Institute of Health and Welfare. Cervical Screening in Australia 2005-2006. Australian Institute of Health and Welfare. https://www.aihw.gov.au/getmedia/1ed28fc5-e086-4a71-a513-c01dfc65785e/csa05-06.pdf.aspx?inline=true. Accessed 17 Dec 2018. | National screening or vaccination program |
| Binns PL, Condon JR. Participation in cervical screening by Indigenous women in the Northern Territory: a longitudinal study. Med J Aus. 2006:185(9);490-494. | National screening or vaccination program |
| Christou A, Katzenellenbogen JM, Thompson SC. Australia's National Bowel Cancer Screening Program: does it work for Indigenous Australians? BMC Public Health. 2010;10(1):21. | National screening or vaccination program |
| Christou A, Thompson SC. Missed opportunities in educating Aboriginal Australians about bowel cancer screening: whose job is it anyway? Contemp Nurse. 2013;46(1):59-69. | National screening or vaccination program |
| Christou A, Thompson SC. Colorectal cancer screening knowledge, attitudes and behavioural intention among Indigenous Western Australians. BMC Public Health. 2012;12:16. | National screening or vaccination program |
| Deng L, Reekie J, Ward JS, Hayen A, Kaldor JM et al. Trends in the prevalence of hepatitis B infection among women giving birth in New South Wales. Med J Aus. 2017;206(7):301-305. | National screening or vaccination program |
| Dunn N et al. Has mammographic screening delivered the expected mortality benefits for Queensland women? Asia Pac J Clin Oncol. 2016;12:87. | National screening or vaccination program |
| Morrice MG, Hewson E, Loria C, Forsyth A, Versace V. More poo in the post: A community health approach to increasing bowel cancer screening and follow-up care in South West Victoria. J Gastroenterol Hepatol. 2016;31:58. | National screening or vaccination program |
| Pilkington L, Haigh M, Durey A, Katzenellenbogen JM, Thompson SC. Perspectives of Aboriginal women on participation in mammographic screening: a step towards improving services. BMC Public Health. 2017;17:13. | National screening or vaccination program |
| Roder D, Webster F, Hastings P, Sinclair SE, Sturrock C et al. Breast cancer screening and survival in aboriginal and torres strait islander women. Asia Pac J Clin Oncol.2011;7:106. | National screening or vaccination program |
| Sun JD, March S, Ireland MJ, Crawford-Williams F, Goodwin B et al. Socio-demographic factors drive regional differences in participation in the National Bowel Cancer Screening Program - An ecological analysis. Aust NZJ Public Health. 2018;42(1):92-97. | National screening or vaccination program |
| Ward PR, Javanparast S, Matt M, Martini A, Tsourtos G et al. Equity of colorectal cancer screening: cross-sectional analysis of National Bowel Cancer Screening Program data for South Australia. ANZ J Public Health. 2011;35(1):61-65. | National screening or vaccination program |
| Ward PR, Coffey C, Meyer S. Trust, choice and obligation: a qualitative study of enablers of colorectal cancer screening in South Australia. Sociol Health Illn. 2015;37(7):988-1006. | National screening or vaccination program |
| Ward PR, Javanparast S, Wilson C. Equity of colorectal cancer screening: Which groups have inequitable participation and what can we do about it? Aust J Prim Health. 2011;17(4)334-346. | National screening or vaccination program |
| Wardle E. The Queensland Bowel Cancer Screening Program: What do the participants think of the program? J Gastroenterol Hepatol. 2012;27:48. | National screening or vaccination program |
| Dorrington MS, Herceg A, Douglas K, Tongs J, Bookallil M. Increasing Pap smear rates at an urban Aboriginal Community Controlled Health Service through translational research and continuous quality improvement. Aust J Prim Health. 2015;21(4):417-422. | National screening or vaccination program |
| Ali H, McManus H, O’Connor C, Callander D, Kong M et al. Human papillomavirus vaccination and genital warts in young indigenous Australians: National sentinel surveillance data. Med J Aus. 2017:206(5);204-209. | National screening or vaccination program |
| Ali H, O’Connor C, Callander D, Saulo D, Graham S et al. The impact of HPV vaccine on genital warts in aboriginal Australians: an analysis of national data. Sex Transm Infect. 2015;91(2):78. | National screening or vaccination program |
| Smith MA, Liu B, McIntyre P, Menzies R, Dey A et al. Fall in genital warts diagnoses in the general and indigenous Australian population following implementation of a national human papillomavirus vaccination program: Analysis of routinely collected national hospital data. J. Infec. Dis. 2015;211(1):91-99. | National screening or vaccination program |
| Adams K, Browne J, Palermo C, Radford G. Experiences of urban Australian Indigenous peer mentors in a non-communicable disease prevention program. Alternative. 2016;12(4):425-436. | Not a primary health care delivered program |
| Ballreich J, Burnett A, Frick K, Ho A, Arkapaw L et al. Economic evaluation of an automated retinal image analysis in Australian Aboriginal and Torres Strait Islander populations for detection of diabetic retinopathy. Value Health. 2016;19(3):5. | Not a primary health care delivered program |
| Cargo M et al. Integrating an ecological approach into an Aboriginal community-based chronic disease prevention program: a longitudinal process evaluation. BMC Public Health. 2011;11:299. | Not a primary health care delivered program |
| Livingstone L, Sananikhone C. Building bridges: learning from the experts: building bridges to implement successful life promotion and suicide prevention expertise across Aboriginal communities. Centre for Rural and Remote Mental Health Queensland. 2010. https://aodknowledgecentre.ecu.edu.au/healthinfonet/getContent.php?linkid=501818&title=Pathways+to+resilience%3A+rural+and+remote+Indigenous+community+suicide+prevention+initiative%3A+final+report. Accessed 10 Dec 2018. | Not a primary health care delivered program |
| Chen D. Cook it, plate it, share it: good quick tukka. Healthinfonet. 2011 https://healthinfonet.ecu.edu.au/healthinfonet/getContent.php?linkid=39696&title=Cook+it%2C+plate+it%2C+share+it%3A+good+quick+tukka+%5Bevaluation+report%5D. Accessed 10 Dec 2018. | Not a primary health care delivered program |
| Deane FP, Capp K, Jones C, De Ramirez D, Lambert G et al. Two-year Follow-up of a Community Gatekeeper Suicide Prevention Program in an Aboriginal Community. Australian Journal of Rehabilitation Counselling. 2006;12(1):21-32. | Not a primary health care delivered program |
| Doyle J, Atkinson-Briggs S, Atkinson P, Firebrace B, Calleja J et al. A prospective evaluation of first people's health promotion program design in the Goulburn-Murray rivers region. BMC Health Services Research. 2016;16:13. | Not a primary health care delivered program |
| Esgin T, Johnston N, Rowley K, Saes De Villarreal E, Newton R. Effect of 12 weeks combined aerobic and resistance training on fitness, arterial stiffness and body composition in Indigenous Australian men and women. J Sci Med Sport. 2017;20:43. | Not a primary health care delivered program |
| Gordon R, Richards N. The chronic care for Aboriginal people program in NSW. NSW Public Health Bull. 2012;23(3):77-80. | Not a primary health care delivered program |
| Hartman D, Wallis G, Drahm M, Unwin R, Robinson D. The Napranum Social and Emotional Wellbeing Week. Australasian Psychiatry. 2009;17:88-91. | Not a primary health care delivered program |
| Jainullabudeen TA, Lively A, Singleton M, Shakeshaft A, Tsey K et al. The impact of a community-based risky drinking intervention (Beat da Binge) on Indigenous young people. BMC Public Health. 2015;15:7. | Not a primary health care delivered program |
| Jeffries-Stokes C, Stokes A, McDonald L. Pulkurlkpa-the western desert kidney health project strategies to build resilience and combat kidney disease and type 2 diabetes. Intern Med J. 2015;45:12. | Not a primary health care delivered program |
| Kanowski LG, Jorm AF, Hart LM. A mental health first aid training program for Australian Aboriginal and Torres Strait Islander peoples: description and initial evaluation. Int J Ment Health Syst. 2009;3:10 | Not a primary health care delivered program |
| Keller N, Johnson R, Gibson B, Jury L, Newchurch L et al. Promoting mental health and wellbeing in Aboriginal contexts: successful elements of suicide prevention work. Health Promot R Austr. 2007;18(3):247-54. | Not a primary health care delivered program |
| Kitchener BA, Jorm AF. Mental Health First Aid: an international programme for early intervention. Early Interv Psychiatry. 2008;2(1):55-61. | Not a primary health care delivered program |
| Malseed C. Deadly Choices Health Promotion Initiative Evaluation Report. Lowitja Institute. 2013 https://www.lowitja.org.au/sites/default/files/docs/Deadly-Choices-Evaluation-Report-v2.pdf. Accessed 9 Dec 2018 | Not a primary health care delivered program |
| Malseed C, Nelson A, Ware R, Lacey I, Lander K. Deadly Choices™ community health events: a health promotion inititative for urban Aboriginal and Torres Strait Islander People. Aust J Prim Health. 2014;20(4):379-383. | Not a primary health care delivered program |
| Nilson C, Kearing-Salmon K, Morrison P, Fetherston C. An ethnographic action research study to investigate the experiences of Bindjareb women participating in the cooking and nutrition component of an Aboriginal health promotion programme in regional Western Australia. Public Health Nutr. 2015;18(18):3394-405 | Not a primary health care delivered program |
| Rankin P, Morton D, Kent L, Mitchell B. A community-based lifestyle intervention targeting Type II Diabetes risk factors in an Australian Aboriginal population: a feasibility study. Australian Indigenous HealthBulletin. 2016;16(3):1-6. | Not a primary health care delivered program |
| Tighe J, McKay K. Alive and Kicking Goals!: Preliminary findings from a Kimberley suicide prevention program. Advances in Mental Health. 2012;10(3):240-245. | Not a primary health care delivered program |
| Trute M, Black T, Abeypala U, Chalmers L. You2-live well with gestational diabetes. Diabetes. 2013;5:70. | Not a primary health care delivered program |
| Zmudzki F, Valentine K, Katz I, Loebel A, Bates S et al. Evaluation of Intensive Home Based Support Services. University of New South Wales. https://www.sprc.unsw.edu.au/media/SPRCFile/1_SPRC_Report__Evaluation_of_Intensive_Home_Based_Support_Services_v2.pdf. Accessed 8 Dec 2018. | Not a primary health care delivered program |
| Ashman AM, Collins CE, Brown LJ, Rae KM, Rollo ME. Validation of a smartphone image-based dietary assessment method for pregnant women. Nutrients. 2017;9(73):1-17. | Not a primary health care delivered program |
| Australian Network for Promotion, Prevention and Early Intervention for Mental Health. Mental health promotion and illness prevention: Aboriginal and Torres Strait Islander people. Flinders University. 2008. http://pandora.nla.gov.au/pan/10581/20080706-1238/auseinet.flinders.edu.au/resources/auseinet/auseinetter_30.pdf. Accessed 10 Dec 2018 | Not a primary health care delivered program |
| House of Representatives, Parliament of the Commonwealth of Australia. Sport - more than just a game: contribution of sport to Indigenous wellbeing and mentoring / House of Representatives Standing Committee on Aboriginal and Torres Strait Islander Affairs. Parliament of Commonwealth of Australia. 2013. <https://www.aph.gov.au/Parliamentary_Business/Committees/House_of_Representatives_Committees?url=atsia/sport/report.htm>. Accessed 13 Dec 2018. | Not a primary health care delivered program |
| Cuesta-Briand B, Bessarab D, Shahid S, Thompson S. 'Connecting tracks': exploring the roles of an Aboriginal women's cancer support network. Health Soc Care Community. 2016;24(6):779-788. | Not a primary health care delivered program |
| Livingstone L, Sananikhone C. Pathways to resilience: rural and remote Indigenous community suicide prevention initiative: final report. Centre for Rural and Remote Health Queensland. 2010. https://aodknowledgecentre.ecu.edu.au/healthinfonet/getContent.php?linkid=501818&title=Pathways+to+resilience%3A+rural+and+remote+Indigenous+community+suicide+prevention+initiative%3A+final+report. Accessed 13 Dec 2018. | Not a primary health care delivered program |
| Clark RA, Fredericks B, Adams M, Atherton J, Howie-Esquivel J et al. Addressing health literacy and cultural teaching issues in Australian Indigenous and non-Indigenous heart failure patients using avatars: Technology development and pilot testing. J Card Fail. 2014;20(8):S115. | Not a primary health care delivered program |
| Cultural and Indigenous Research Centre Australia. Evaluation of the NSW Knockout Weight Loss Challenge pilot project: final report - executive summary. NSW ministry of health. 2012. http://www.preventivehealth.net.au/uploads/2/3/5/3/23537344/nsw_knockout_weight_loss_challenge_-_pilot_evaluation_-_exec_summary.pdf. Accessed 14 Dec 2018. | Not a primary health care delivered program |
| Campbell S, Bohanna I, McKeown-Young D, Esterman A, Cadet-James Y et al. Evaluation of a community-based tabacco control intervention in five remote north Queensland Indigenous communities. Int J Health Promot Educ. 2014;52(2):78-89. | Not a primary health care delivered program |
| Boudville A, Anjou M, Hugh T. Improving eye care for Indigenous Australians in primary health care settings. Aust J Rural Health. 2013:21(1);121-127. | Not a program evaluation |
| Chung F, Herceg A, Bookallil M. Diabetes clinic attendance improves diabetes management in an urban Aboriginal and Torres Strait Islander population. Aust Fam Physician. 2014;43(11):797-802. | Not a program evaluation |
| Cosh S, Maksimovic L, Ettridge K, Copley D, Bowden JA. Aboriginal and Torres Strait Islander utilisation of the Quitline service for smoking cessation in South Australia. Aust J Prim Health. 2013;19:113-118 | Not a program evaluation |
| Fittock M, Edwards K. Preventing recurrent acute rheumatic fever- systems go approach. Global Heart. 2016;11(2). | Not a program evaluation |
| Genat B, Browne J, Thorpe S, MacDonald C. Sectoral system capacity development in health promotion: Evaluation of an Aboriginal nutrition program. Health Promot J Austr: 2016;27(3):236-242. | Not a program evaluation |
| Larson A. Indigenous Healthy Lifestyle Program evaluation: final report. Department of Health. 2010. http://socialdimensions.com.au/wp-content/uploads/IHLP-report-FINAL.pdf. Accessed 15 Dec 2018. | Not a program evaluation |
| Larson A, Gordon G. Evidence-based success factors for community-based Aboriginal health projects. In Proceedings of the 11^th^ National Rural Health Conference; 2011 March 13-16, Perth. National Rural Health Conference. http://ruralhealth.org.au/11nrhc/papers/11th%20NRHC%20Larson_Ann_C2.pdf. Accessed 7 Dec 2018. | Not a program evaluation |
| Polus BI, Paterson C, Van Rotterdam J, Vindigni D. Embedding chiropractice in Indigenous Health Care Organisations: applying the normalisation process model. BMC Health Services Research. 2012;12:429. | Not a program evaluation |
| Janca A, Lyons Z. Rural and remote psychiatry symposium paper 1: Development and evaluation of an aboriginal mental health screening tool. Aust N Z J Psychiatry. 2012;46:14 | Not a program evaluation |
| Janca A, Lyons Z, Balaratnasingam S, Parfitt D, Davison S et al. Here and Now Aboriginal Assessment: Background, development and preliminary evaluation of a culturally appropriate screening tool. Australas Psychiatry. 2015;23(3):287-292. | Not a program evaluation |
| Janca, A. Development and evaluation of an aboriginal mental health screening instrument. Eur. Psychiatry. 2010;25(1):542. | Not a program evaluation |
| Esler DM, Johnston F, Thomas D. The acceptability of a depression screening tool in an urban Aboriginal community-controlled health service. Aust N Z J Public Health. 2007;31(3):259-263. | Not a program evaluation |
| Kowal E, Gunthorpe W, Bailie RS. Measuring emotional and social wellbeing in Aboriginal and Torres Strait Islander populations: an analysis of a Negative Life Events Scale. Int J Equity Health. 2007;6(18):1-12. | Not a program evaluation |
| Cartwright K, Gray D, Fewings E. Demonstrating impact: Lessons learned from the Queensland aboriginal and islander health council's aod-our-way program. Int J Environ Rese Public Health. 2018;15(3). | Not a program evaluation |
| Dreher B, Fredericks B, Mahoney R, Merzliakov N. Mildura, Victoria: Aboriginal Health Promotion Short Course. Aborig and Isl Health Work J. 2009;33(6):6-7. | Not a program evaluation |
| Nancarrow H, Wilcoxon H, Hanks E, Sinclair S, Zorbas D. Evaluation of well women workshops for delivering breast awareness and early detection messages to indigenous women through partnerships. Asia Pac J Clin Oncol. 2011;7:182. | Not a program evaluation |
| Adams K, Burns C, Liebzeit A, Ryschka J, Thorpe S et al. Use of participatory research and photo-voice to support urban Aboriginal healthy eating. Health Soc Care Community. 2012;20(5):497-505. | Not a program evaluation |
| Adams, M, Gleeson, K, Supramaniam, R, Earnshaw, T. Engaging Aboriginal and Torres Strait Islander Communities in Prostate Cancer Health Care Programs. Prostate Cancer Foundation of Australia. 2015. http://www.prostate.org.au/media/591619/ATSI-Monographs-in-Prostate-Cancer.pdf. Accessed 9 Dec 2018. | Not a program evaluation |
| Arnold LW, Hoy WE, Sharma SK, Wang ZQ. The association between HbA1C and Cardiovascular Disease Markers in a Remote Indigenous Australian Community with and without Diagnosed Diabetes. J Diabetes Res. 2016:1-8. | Not a program evaluation |
| Bonner A, Gillespie K, Campbell KL, Corones-Watkins K, Hayes B et al. Evaluating the prevalence and opportunity for technology use in chronic kidney disease patients: a cross-sectional study. BMC Nephrology. 2018;19(28):1-8. | Not a program evaluation |
| Boudville A, Anjou M, Hugh T. Let's fix the diabetes pathway: improving Indigenous eye care. Clin Exp Ophthalmol. 2013;41:77. | Not a program evaluation |
| Bovill M, Bar-Zeev Y, Gruppetta M, O’Mara P, Cowling B et al. Collective and negotiated design for a clinical trial addressing smoking cessation supports for Aboriginal and Torres Strait Islander mothers in NSW, SA and QLD - developing a pilot study. Aust J Prim Health. 2017;23:497-503. | Not a program evaluation |
| Calabria B, Clifford A, Shakeshaft A, Allan J, Bliss D et al. The acceptability to Aboriginal Australians of a family-based intervention to reduce alcohol-related harms. Drug Alcohol Rev. 2013;32:328-332. | Not a program evaluation |
| Campbell D. Aboriginal involvement in caring-for-country: an economic case study in primary preventative health. Australas Psychiatry. 2015;23(6):623-625. | Not a program evaluation |
| Conway J, Tsourtos G, Lawn S. The barriers and facilitators that Indigenous health workers experience in their workplace and communities in providing self-management support: a multiple case study. BMC Health Services research. 2017;17:319. | Not a program evaluation |
| Cox A, Dudgeon P, Holland C, Kelly K, Scrine C et al. Using participatory action research to prevent suicide in Aboriginal and Torres Strait Islander communities. Aust J PrimHealth. 2014;20(4):345-349. | Not a program evaluation |
| Doyle J, Saggers S, Fisher C. Alcohol interventions programs within Australian prisons for Aboriginal and Torres Strait Islander men. Drug Alcohol Rev. 2011;30:27-28. | Not a program evaluation |
| Dunt D, Anjou M, Bouville A, Hsueh A, Taylor H. Establishing the value of Indigenous eye health programs: health needs, economic priority and performance assessment approaches. Aust Health Rev. 2014;38(1):99-105. | Not a program evaluation |
| Glover M, Kira A, Johnston V, Walker N, Brown N et al. Australian and New Zealand Indigenous mothers' report respect for smoking bans in homes. Women Birth. 2015;28(1):1-7. | Not a program evaluation |
| Goodwin-Smith I, Hicks N, Hawke M, Alver G, Raftery P. Living beyond Aboriginal suicide: developing a culturally appropriate and accessible suicide postvention service for Aboriginal communities in South Australia. Advances in Mental Health. 2013;11(3):238-245. | Not a program evaluation |
| Harvey PW, Petkov J, Kowanko I, Helps Y, Battersby M. Chronic condition management and self-management in Aboriginal communities in South Australia: Outcomes of a longitudinal study. Aust Health Rev. 2013;37(2):246-250. | Not a program evaluation |
| Barnett L, Kendall E. Culturally appropriate methods for enhancing the participation of Aboriginal Australians in health-promoting programs. Health Promot J Aust. 2011;22(1):27-32. | Not a program evaluation |
| Maguire G, Molanus B, Tchan M, Currie B. One year of weekly azithromycin provides no benefit for indigenous australians living with COPD in remote Australia. Respirology. 2010;15:22. | Not a program evaluation |
| Reilly RE, Doyle D, Bretherton K, Rowley G, Harvey JL et al. Identifying psychosocial mediators of health amongst Indigenous Australians for the Heart Health Project. Ethn Health. 2008;13(4):351-373. | Not a program evaluation |
| Ward PR, Coffey C, Javanparast S, Wilson C, Meyer SB. Institutional (mis)trust in colorectal cancer screening: a qualitative study with Greek, Iranian, Anglo-Australian and Indigenous groups. Health Expect. 2015;18(6):2915-2927. | Not a program evaluation |
| Wilson A, Magarey A, Jones M, Kelly J. Strategies for best practice in community-based obesity prevention. Obes Res Clinl Pract. 2011;5:S43. | Not a program evaluation |
| Wynaden D. Building mental wealth: Improving mental health outcomes for Indigenous Australians. Int J Ment Health Nurs. 2009;18:27. | Not a program evaluation |
| Davies J, Bukulatjpi S, Sharma S, Caldwell L, Johnston V et al. Development of a culturally appropriate bilingual electronic app about Hepatitis B for Indigenous Australians: Towards shared understandings. JMIR Res Protoc. 2015;4(2):1-13. | Not a program evaluation |
| Dimer L, Van Santen C, Eades D, James P, Henry D. Reducing heart disease in our aboriginal communities. Heart Lung and Circ. 2011;20:7. | Not a program evaluation |
| Gui G, Lau Q. Quick Meals for Kooris: an evaluation. Aborig Isl Health Worker J. 2007;31(4):20-22. | Not a program evaluation |
| Lopes J, Lindeman M, Taylor K, Grant, L. Cross cultural education in suicide prevention: development of a training resource for use in Central Australian Indigenous communities. Advances in Mental Health. 2012;10(3):224-234. | Not a program evaluation |
| Meyer J, Johnson K, Bowyer J, Muir J, Turner A. Evaluating a health video on diabetic retinopathy. Health Promot J Austr. 2016;27(1):84-87. | Not a program evaluation |
| Closing the Gap Clearinghouse. Healthy lifestyle program for physical activity and nutrition. Australian Institute of Health and Welfare. 2011. https://ir.lib.uwo.ca/cgi/viewcontent.cgi?referer=https://www.google.com.au/&httpsredir=1&article=1203&context=aprci. Accessed 10 Dec 2018. | Not a program evaluation |
| Closing the Gap Clearinghouse. Fetal alcohol spectrum disorders: a review of interventions for prevention and management in Indigenous communities. Australian Institute of Health and Welfare. 2015. <https://www.aihw.gov.au/getmedia/778f54f3-5618-428f-a094-40c347ed3c7f/ctgc-rs36.pdf.aspx?inline=true>. Accessed 10 Dec 2018. | Not a program evaluation |
| Clague L, Langford K, Sundquist A, White V, O’Callaghan J et al. Aboriginal cancer journeys: storytelling to support aboriginal people affected by cancer. Asia Pac J Clin Oncol. 2010;6:210. | Not a program evaluation |
| DrugInfo Clearinghouse. Indigenous dual diagnosis project. DrugInfo Clearinghouse. 2009. https://healthinfonet.ecu.edu.au/key-resources/resources/15755/?title=Indigenous%20dual%20diagnosis%20project. Accessed 9 Dec 2018. | Not a program evaluation |
| Jacka C, Garvey G, Martin J, Sabesan S, Ghandi M et al. Closing the divide in indigenous cancer: Cancer brochures for indigenous patients and their families. Asia Pac J Clin Oncol. 2011;7:147-148. | Not a program evaluation |
| Schoen D, Balchin D, Thompson S. Health promotion resources for Aboriginal people: lessons learned from consultation and evaluation of diabetes foot care resources. Health Promot J Austr. 2010;21(1):64-69. | Not a program evaluation |
| Browne J, Thorpe S, D’Amico E, Mitchell C. Feltman: evaluating the acceptability of a diabetes education tool for Aboriginal health workers. Aust J Prim Health. 2014:20;319-322. | Not a program evaluation |
| Clifford, A. Screening and brief intervention in Aboriginal primary health care: towards evidence-based practice. University of New South Wales. 2008. | Not a program evaluation |
| Dingwall KM, Puszka S, Sweet M, Mills PP, Nagel T. Evaluation of a culturally adapted training course in Indigenous e-mental health. Australas Psychiatry. 2015;23(6):630-635. | Not a program evaluation |
| Gould GS, Bar-Zeev Y, Bovill M, Atkins L, Gruppetta M et al. Designing an implementation intervention with the Behaviour Change Wheel for health provider smoking cessation care for Australian Indigenous pregnant women. Implementation Science. 2017;12:114. | Not a program evaluation |
| Gould G. SISTAQUIT (Supporting Indigenous Smokers to Assist Quitting) The SISTAQUIT trial compares usual care to health provider training in culturally-appropriate smoking cessation care for pregnant Aboriginal and/or Torres Strait Islander women. ANZCTR trial registration number: ACTRN12618000972224. 2018. https://www.anzctr.org.au/Trial/Registration/TrialReview.aspx?id=374707. Accessed 16 Dec 2018. | Not implemented |
| Jamieson L, Skilton M, Maple-Brown L, Kapellas K, Askie L et al. Periodontal disease and chronic kidney disease among Aboriginal adults; an RCT. BMC Nephrology. 2015;16:8. | Not implemented |
| Johnson T. Obesity care in the primary health setting: Does a peer-supported, nurse-facilitated lifestyle modification program (“My Health for Life”) lead to better health outcomes for obese patients and improved patient satisfaction with care? ANZCTR trial registration number: Provisional ACTRN1261800062428op. 2018. <https://www.anzctr.org.au/Trial/Registration/TrialReview.aspx?id=374811>. Accessed 10 Dec 2018. | Not implemented |
| Alison J. Managing chronic lung disease in Aboriginal communities: the Breathe Easy Walk Easy-Lungs for Life (BE WELL) project. ANZCTR trial registration number: ACTRN12617001337369. 2017. <https://www.anzctr.org.au/Trial/Registration/TrialReview.aspx?id=373585&isReview=true>. Access 10 Dec 2018. | Not implemented |
| Brazionis L, Jenkins A, Keech A, Ryan C, Bursell S. An evaluation of the telehealth facilitation of diabetes and cardiovascular care in remote Australian Indigenous communities: - protocol for the telehealth eye and associated medical services network [TEAMSnet] project, a pre-post study design. BMC Health Services Research. 2017;17;3. | Not implemented |
| Bonevski B, Paul C, D’Este C, Sanson-Fisher R, West R. RCT of a client-centred, caseworker-delivered smoking cessation intervention for a socially disadvantaged population. BMC Public Health. 2011;11:70. | Not implemented |
| Colagiuri S, Vita P, Cardona-Morrell M, Singh MF, Farrell L et al. The Sydney Diabetes Prevention Program: A community-based translational study. BMC Public Health 2010;10:328. | Not implemented |
| Semenova NB. Modern strategies of preventative measures against suicide in native people: foreign literature review. Suicidology. 2017;8(2):3-20. | Not in English |
| Clapham K, O'Dea K, Chenhall RD. Interventions and sustainable programs. In: Carson B, Dunbar T, Chenhall R, Bailie R. Social determinants of Indigenous Health. Crows Nest: Allen and Uwin Academic; 2007. p. 271-295. | Opinion piece |
| Gould G, McEwan A, Eades S, Sanson-Fisher R, Panaretto K. An intensive smoking intervention for pregnant Aboriginal and Torres Strait Islander women: a randomised controlled trial. Med. J. Aust. 2013;1:23-24. | Opinion piece |
| Hilton DJ. How do good exercise facilities and programs benefit Indigenous Australians. Newsletter of the Public Health Association of Australia. 2008. https://www.phaa.net.au/advocacy-policy/archive. Accessed 18 Dec 2018. | Opinion piece |
| Jeffries-Stokes C, Stokes A, McDonald L, Stokes S, Daly Jeanna. A complex Aboriginal health project and the challenges for evaluation. Aust N Z J Public Health. 2011;35(3):204-206. | Opinion piece |
| Messenger A. Aboriginal quit project gets results. Public health Res Pract. 2015;25(3). | Opinion piece |
| Shephard MD, Gill JP. The National QAAMS Program - A Practical Example of PoCT Working in the Community. The Clin Biochem Rev. 2010;31(3):105-109. | Opinion piece |
| Togni S, Askew D, Rogers, L, Potter N, Egert S et al. Creating safety to explore: Strengthening innovation in an Australian Indigenous primary health care setting through developmental evaluation. In: Patton M, McKegg K, Wehipeihana N. Development evaluation exemplars: Principles in practice. New York: The Guilford Press; 2016. p. 234-251. | Opinion piece |
| Department of Health. Indigenous Australians' Health Programme. Australian Government. 2018. <http://www.health.gov.au/internet/main/publishing.nsf/Content/indigenous-austrailans-health-program-evaluation>. Accessed 11 Dec 2018. | Program not evaluated or primary health care program component not evaluated |
| Baum F, Freeman T, Jolley G, Lawless A, Bentley M. Health promotion in Australian multi-disciplinary primary health care services: case studies from South Australia and the Northern Territory. Health Promot Int. 2013;29(4):705-719. | Program not evaluated or primary health care program component not evaluated |
| Burak N, Carroll C. Kooris Cooking Healthy recipe cards. In: Proceedings of the National Nutrition Networks Conference; 2008, Alice Springs. Nutrition Networks Conference Management Committee. https://healthinfonet.ecu.edu.au/key-resources/publications/398/?title=Koori%27s%20Cooking%20Healthy%20recipe%20cards. Accessed 12 Dec 2018. | Program not evaluated or primary health care program component not evaluated |
| Conigrave K, Freeman B, Caroll T, Simpson L, Lee K et al. The Alcohol Awareness project: community education and brief intervention in an urban Aboriginal setting. Health Promot J Austr. 2012;23(3):219-25. | Program not evaluated or primary health care program component not evaluated |
| Gould GS. The Indigenous Counselling and Nicotine (ICAN) QUIT in Pregnancy Step-Wedge Pilot Study: feasibility of training health providers in evidence based smoking cessation care for Australian Indigenous pregnant smokers. ANZCTR trial registration number: ACTRN12616001603404. 2016. https://www.anzctr.org.au/Trial/Registration/TrialReview.aspx?id=371778. Accessed 12 Dec 2018 | Program not evaluated or primary health care program component not evaluated |
| Sunshames A. A pragmatic trial of an 8-week physical activity program for Australian Indigenous adults with/or at risk of chronic disease, to improve functional capacity. ANZCTR trial registration number: ACTRN12616000497404. 2016. https://www.anzctr.org.au/Trial/Registration/TrialReview.aspx?id=369905. Accessed 12 Dec 2018. | Program not evaluated or primary health care program component not evaluated |
| National Evaluation of Bringing Them Home and Indigenous Mental Health Programs. Aboriginal and Islander Health Worker Journal. 2006;30(4):25. | Program not evaluated or primary health care program component not evaluated |
| Australians for Native Title and Reconciliation. Success stories in Indigenous health: a showcase of successful Aboriginal and Torres Strait Islander health projects. Australians for Native Title and Reconciliation. 2007. https://antar.org.au/sites/default/files/successstories.pdf. Accessed 13 Dec 2018. | Program not evaluated or primary health care program component not evaluated |
| Black S, Ndaba A, Kerr C, Doyle B. Methadone, Counselling and Literacy: A health literacy partnership for Aboriginal clients. Lit Numer Stud. 2012:20(1);45-62. | Program not evaluated or primary health care program component not evaluated |
| Bovill M, Bar-Zeev Y, Bonevski B, Gruppetta M, Palazzi K et al. The growth and empowerment measure among aboriginal pregnant women recruited for I can quit pregnancy. Asia Pac J Clin Oncol. 2017;13:32. | Program not evaluated or primary health care program component not evaluated |
| Bowditch C. Deadly Mereny Noonak Ngyn Moort: good food for my people: mid-term evaluation; In: Proceedings of the 12^th^ National Rural Health Conference; 2013 April 7-10, Adelaide. National Rural Health Conference. http://www.ruralhealth.org.au/12nrhc/wp-content/uploads/2013/06/Bowditch-Claire_ppr.pdf. Accessed 11 Dec 2018. | Program not evaluated or primary health care program component not evaluated |
| Callaghan K. Let's yarn about lung cancer - An Indigenous Community Project in Regional Australia. J Thorac Oncol. 2015;10(9). | Program not evaluated or primary health care program component not evaluated |
| Dingwall K. Wellbeing Intervention for Chronic Kidney Disease (WICKD): A Trial of the Aboriginal and Islander Mental Health Initiative (AIMhi) Stay Strong App. ANZCTR trial registration number: ACTRN12617000249358. 2017. https://www.anzctr.org.au/Trial/Registration/TrialReview.aspx?id=371512. Accessed 17 Dec 2018. | Program not evaluated or primary health care program component not evaluated |
| Kildea S. Assessment of the acceptability, feasibility and impact on smoking cessation, of an intensive smoking cessation intervention, including financial incentives, among pregnant Indigenous women reporting daily smoking and receiving maternity care through the Birthing in Our Community program. ANZCTR trial registration number: ACTRN12615001278527. 2015. <https://www.anzctr.org.au/Trial/Registration/TrialReview.aspx?id=369261>. Accessed 17 Dec 2018. | Program not evaluated or primary health care program component not evaluated |
| Tracey K, Cossich T, Bennett P, Wright S, Ockerby C. A nurse-managed kidney disease program in regional and remote Australia. Renal Society of Australasia Journal. 2013;9(1):28-34. | Program not evaluated or primary health care program component not evaluated |
| Vindigni D, Polus B, Edgecombe G, Van Rotterdam J, Turner N et al. Bringing Chiropractic to Aboriginal Communities: The Durri Model. Chiropr J Aust. 2009;39(2):80-83. | Program not evaluated or primary health care program component not evaluated |
| Jones K, Wynne C, Anjou MD, Talor HR. Closing the gap for vision: developing a culturally appropriate Indigenous health promotion strategy for diabetes related eye care. Presented at: Indigenous Eye Health; 2015, Adelaide. Indigenous Eye Health. https://www.researchgate.net/publication/280918186_Closing_the_Gap_for_Vision_Developing_a_culturally_appropriate_Indigenous_health_promotion_strategy_for_diabetes_related_eye_care. Access 18 Dec 2018. | Program not evaluated or primary health care program component not evaluated |
| Ivers RG, Castro A, Parfitt D, Ballie RS, D’Abbs PH et al. Evaluation of a multi-component community tobacco intervention in three remote Australian Aboriginal communities. Aust N Z J Public Health. 2006;30(2):132-136. | Program not evaluated or primary health care program component not evaluated |
| Reilly RE, Cincotta M, Doyle J, Firebrace BR, Cargo M et al. A pilot study of Aboriginal health promotion from an ecological perspective. BMC Public Health. 2011;11:9. | Program not evaluated or primary health care program component not evaluated |
| Shephard M, O’Brien C, Burgoyne A, Croft J, Garlett T et al. Review of the cultural safety of a national Indigenous point-of-care testing program for diabetes management. Aust J Prim Health. 2016;22(4):368-374. | Program participant outcomes not reported |
| Clifford A, Pulver LJ, Richmond R, Shakeshaft A, Ivers R. Smoking, nutrition, alcohol and physical activity interventions targeting Indigenous Australians: rigorous evaluations and new directions needed. Aust N Z J Public Health. 2011;35(1):38-46. | Sub-studies met inclusion criteria but already included in search, or sub-studies did not meet inclusion criteria |
| DiGiacomo M, Davidson PM, Abbott PA, Davison J, Moore L et al. Smoking cessation in Indigenous populations of Australia, New Zealand, Canada, and the United States: elements of effective interventions. Int J Environ Res Public Health. 2011;8(2):388-410. | Sub-studies met inclusion criteria but already included in search, or sub-studies did not meet inclusion criteria |
| Dudgeon P, Walker R, Scrine C, Shephard C, Calma T et al. Effective strategies to strengthen the mental health and wellbeing of Aboriginal and Torres Strait Islander people. Closing the Gap Clearinghouse. 2014. <https://www.aihw.gov.au/getmedia/6d50a4d2-d4da-4c53-8aeb-9ec22b856dc5/ctgc-ip12-4nov2014.pdf.aspx?inline=true>. Accessed 18 Dec 2018. | Sub-studies met inclusion criteria but already included in search, or sub-studies did not meet inclusion criteria |
| Gibson O, Lisy K, Davy C, Aromataris E, Kite E et al. Enablers and barriers to the implementation of primary health care interventions for Indigenous people with chronic diseases: a systematic review. Implementation Sci. 2015;10:71. | Sub-studies met inclusion criteria but already included in search, or sub-studies did not meet inclusion criteria |
| Ivers R. Anti-tobacco programs for Aboriginal and Torres Strait Islander people. Australian Institute of Health and Welfare. 2011. https://www.aihw.gov.au/getmedia/95b3eccf-be44-4019-91c9-52e207456cf7/ctgc-rs04.pdf.aspx?inline=true. Accessed 12 Dec 2018. | Sub-studies met inclusion criteria but already included in search, or sub-studies did not meet inclusion criteria |
| Iyngkaran P, Toukhsati SR, Harris M, Connors C, Kangaharan N et al. Self-Managing Heart Failure in Remote Australia - Translating Concepts into Clinical Practice. Curr Cardiol Rev. 2013;12(4):270-284. | Sub-studies met inclusion criteria but already included in search, or sub-studies did not meet inclusion criteria |
| Johnston L, Doyle J, Morgan B, Atkinson-Briggs S, Firebrace B et al. A review of programs that targeted environmental determinants of Aboriginal and Torres Strait Islander health. Int J Environ Res Public Health. 2013;10(8):3518-3542. | Sub-studies met inclusion criteria but already included in search, or sub-studies did not meet inclusion criteria |
| Leske S, Harris MG, Charlson FJ, Ferrari AJ, Baxter AJ et al. Systematic review of interventions for Indigenous adults with mental and substance use disorders in Australia, Canada, New Zealand and the United States. Aust N Z J Psychiatry. 2016;50(11):1040-1054. | Sub-studies met inclusion criteria but already included in search, or sub-studies did not meet inclusion criteria |
| Liaw T, Furler J, Pyett P, Kelaher M, Rowley K et al. Chronic Disease interventions for Aboriginal Australians - a review of the literature. Aust N Z J Public Health. 2011;35(3):238-248. | Sub-studies met inclusion criteria but already included in search, or sub-studies did not meet inclusion criteria |
| Macniven R, Elwell M, Ride K, Bauman A, Richards J. A snapshot of physical activity programs targeting Aboriginal and Torres Strait Islander people in Australia. Health Promot J Austr. 2017;28(3):185-206. | Sub-studies met inclusion criteria but already included in search, or sub-studies did not meet inclusion criteria |
| McNamara BJ, Sanson-Fisher R, D’Este C, Eades S. Type 2 diabetes in Indigenous populations: Quality of intervention research over 20 years. Prev Med. 2011;52(1):3-9. | Sub-studies met inclusion criteria but already included in search, or sub-studies did not meet inclusion criteria |
| Moore E, Lawn S, Oster C, Morello A. Self-management programs for Aboriginal and Torres Strait Islander Peoples with chronic conditions: A rapid review. Chronic illness. 2017;0(0):1-41. | Sub-studies met inclusion criteria but already included in search, or sub-studies did not meet inclusion criteria |
| Passey ME, Bryant J, Hall AE, Sanson-Fisher R. How will we close the gap in smoking rates for pregnant Indigenous women? Med J Aust. 2013;199(1):39-41. | Sub-studies met inclusion criteria but already included in search, or sub-studies did not meet inclusion criteria |
| Power J, Grealy C, Rintoul D. Tobacco interventions for Indigenous Australians: a review of current evidence. Health Promot J Austr. 2009;20(3):186-194. | Sub-studies met inclusion criteria but already included in search, or sub-studies did not meet inclusion criteria |
| Schembri L, Curran J, Collins L, Prelinovskaia M, Bell H et al. The effect of nutrition education on nutrition-related health outcomes of Aboriginal and Torres Strait Islander people: a systematic review. Aust N Z J Public Health. 2016;40:42-47. | Sub-studies met inclusion criteria but already included in search, or sub-studies did not meet inclusion criteria |
| Sunshames A, Van Uffelen JGZ, Gebel K. Do physical activity interventions in Indigenous people in Australia and New Zealand improve activity levels and health outcomes? A systematic review. Int J Behav Nutr Phys Act. 2016;13:129. | Sub-studies met inclusion criteria but already included in search, or sub-studies did not meet inclusion criteria |
| Tapp RJ, Svoboda J, Fredericks B, Jackson AJ, Taylor HR. Retinal Photography Screening Programs to Prevent Vision Loss from Diabetic Retinopathy in Rural and Urban Australia: A Review. Opthalmic Epidemiol. 2015;22(1):52-59. | Sub-studies met inclusion criteria but already included in search, or sub-studies did not meet inclusion criteria |
| TNS Social Research. Environmental scan of tobacco control interventions in Aboriginal population: what works? What doesn't? Final report. Western Australian Department of Health. 2008. https://healthinfonet.ecu.edu.au/key-resources/publications/16663/?title=Environmental%20scan%20of%20tobacco%20control%20interventions%20in%20Aboriginal%20population%3A%20what%20works%3F%20what%20doesn%27t%3F%20Final%20report. Accessed 10 Dec 2018. | Sub-studies met inclusion criteria but already included in search, or sub-studies did not meet inclusion criteria |
| Tiwari T, Jamieson L, Broughton J, Lawrence HP, Batliner TS et al. Reducing Indigenous Oral Health Inequalities: A Review from 5 Nations. J Dent Res. 2018;97(8):869-877. | Sub-studies met inclusion criteria but already included in search, or sub-studies did not meet inclusion criteria |
| Upton P, Davey R, Evans M, Mikhailovich K, Simpson L et al. Tackling Indigenous Smoking and Healthy Lifestyle Programme review: a rapid review of the literature. University of Canberra. 2014. http://www.health.gov.au/internet/main/publishing.nsf/Content/904B8752C99678A1CA257EA00026976F/$File/TIS-and-Healthy-Lifestyle-Programme-Review_A-Rapid-Review-of-the-Literature.pdf. Access 12 Dec 2018. | Sub-studies met inclusion criteria but already included in search, or sub-studies did not meet inclusion criteria |
| Alston LV, Peterson KL, Jacobs JP, Allender S, Nichols M. A systematic review of published interventions for primary and secondary prevention of ischaemic heart disease (IHD) in rural populations of Australia. BMC Public Health. 2016:16(895);1-11. | Sub-studies met inclusion criteria but already included in search, or sub-studies did not meet inclusion criteria |
| Ashman AM, Brown LJ, Collins CE, Rollo ME, Rae KM. Factors associated with effective nutrition interventions for pregnant indigenous women: a systematic review. J acad nutr diet. 2017:117(6);1222-1253. | Sub-studies met inclusion criteria but already included in search, or sub-studies did not meet inclusion criteria |
| Black A. Evidence of effective interventions to improve the social and environmental factors impacting on health: informing the development of Indigenous Community Agreements. Department of Health. 2007. http://www.health.gov.au/internet/main/publishing.nsf/Content/health-oatsih-pubs-evidence. Accessed 16 Dec 2018. | Sub-studies met inclusion criteria but already included in search, or sub-studies did not meet inclusion criteria |
| Browne J, Adams K, Atkinson P, Gleeson D, Hayes R. Food and nutrition programs for Aboriginal and Torres Strait Islander Australians: an overview of systematic reviews. Aust Health Rev. 2017;42(6):689-697. | Sub-studies met inclusion criteria but already included in search, or sub-studies did not meet inclusion criteria |
| Calabria B, Clifford A, Shakeshaft A, Doran C. A systematic review of family-based interventions targeting alcohol misuse and their potential to reduce alcohol related harm in Indigenous communities. J Stud Alcohol drugs. 2012;73(3):477-488. | Sub-studies met inclusion criteria but already included in search, or sub-studies did not meet inclusion criteria |
| Brusse C, Gardner K, Dowden M. Social Media and Mobile Apps for Health Promotion in Australian Indigenous Populations: Scoping Review. J Med Internet Res. 2014;16(12):e280. | Sub-studies met inclusion criteria but already included in search, or sub-studies did not meet inclusion criteria |
| Carson K, Jayasinghe H, Ali A, Singh K, Peters M et al. Culturally-tailored interventions for smoking cessation in Indigenous populations: A cochrane systematic review and meta-analysis. Respirology. 2015;20:17. | Sub-studies met inclusion criteria but already included in search, or sub-studies did not meet inclusion criteria |
| Carson KV, Brinn MP, Peters M, Veale A, Esterman AJ et al. Interventions for tobacco use cessation in indigenous populations: A cochrane meta-analysis. Am J Respir Crit Care Med. 2014;189:A1086. | Sub-studies met inclusion criteria but already included in search, or sub-studies did not meet inclusion criteria |
| Carson KV, Brinn MP, Peters M, Veale A, Esterman AJ et al. Interventions for smoking cessation in Indigenous populations. Cochrane Database Syst Rev. 2012;1:1-52. | Sub-studies met inclusion criteria but already included in search, or sub-studies did not meet inclusion criteria |
| Clelland N, Gould T, Parker E. Searching for evidence: What works in Indigenous mental health promotion? Health Promot J Austr. 2007;18(3):208-216. | Sub-studies met inclusion criteria but already included in search, or sub-studies did not meet inclusion criteria |
| Clifford AC, Doran CM, Tsey K. A systematic review of suicide prevention interventions targetting Indigenous peoples in Australia, United States, Canada and New Zealand. BMC Public Health. 2013;13:463. | Sub-studies met inclusion criteria but already included in search, or sub-studies did not meet inclusion criteria |
| Day A, Francisco A. Social and emotional wellbeing in Indigenous Australians: identifying promising interventions. Aust N Z J Public Health. 2013;37(4):350-355. | Sub-studies met inclusion criteria but already included in search, or sub-studies did not meet inclusion criteria |
| Gibson OR, Segal L. Limited evidence to assess the impact of primary health care system or service level attributes on health outcomes of Indigenous people with type 2 diabetes: a systematic review. BMC Health Serv Res. 2015;15:154. | Sub-studies met inclusion criteria but already included in search, or sub-studies did not meet inclusion criteria |
| Gilligan C, Sanson-Fisher R, Eades S, D’Este C. Antenatal smoking in vulnerable population groups: An area of need. Journal of Obstetrics and Gynaecology. 2007;27(7):664-671. | Sub-studies met inclusion criteria but already included in search, or sub-studies did not meet inclusion criteria |
| Headey A, Pirkis J, Merner B, Vanden-Heuvel A, Mitchell P et al. A review of 156 local projects funded under Australia's National Suicide Prevention strategy: Overview and lessons learned. Aust E J Adv Mental Health. 2006;5(3):1-15. | Sub-studies met inclusion criteria but already included in search, or sub-studies did not meet inclusion criteria |
| Huffman MD, Galloway JM. Cardiovascular Health in Indigenous Communities: Successful programs. Heart Lung and Circ. 2010;19(5-6):351-260. | Sub-studies met inclusion criteria but already included in search, or sub-studies did not meet inclusion criteria |
| Jiwa A, Kelly L, St. Pierre-Hansen N. Healing the community to heal the individual: Literature review of aboriginal community-based alcohol and substance abuse programs. Can Fam Physician. 2008;54(7):1000-1000. | Sub-studies met inclusion criteria but already included in search, or sub-studies did not meet inclusion criteria |
| Johnston V, Westphal DW, Glover M, Thomas DP, Segan C et al. Reducing smoking among Indigenous populations: new evidence from a review of trials. Nicotine Tob Res. 2013;15(8):1329-1338. | Sub-studies met inclusion criteria but already included in search, or sub-studies did not meet inclusion criteria |
| Katz IJ, Hoy WE, Kondalsamy-Chennakesavan S, Gerntholtz T, Scheppingen J et al. Chronic kidney disease management--what can we learn from South African and Australian efforts? Blood purif. 2006;24(1):115-22. | Sub-studies met inclusion criteria but already included in search, or sub-studies did not meet inclusion criteria |
| Lindstedt S, Moeller-Saxone K, Black C, Herrman H, Szwarc J. Realist Review of programs, policies and interventions to enahnce the social, emotional and spiritual well-being of Aboriginal and Torres Strait Islander young people living in out-of-home care. International Indigenous Policy Journal. 2017;8(3):5. | Sub-studies met inclusion criteria but already included in search, or sub-studies did not meet inclusion criteria |
| McCalman J, Tsey K, Clifford A, Earles W, Shakeshaft A et al. Applying what works: a systematic search of the transfer and implementation of promising Indigenous Australian health services and programs. BMC Public Health. 2012;12:600. | Sub-studies met inclusion criteria but already included in search, or sub-studies did not meet inclusion criteria |
| McCalman J, Tsey K, Wenitong M, Wilson A, McEwan A et al. Indigenous men's support groups and social and emotional wellbeing: a meta-synthesis of the evidence. Aust J Prim Health. 2010;16(2):159-166. | Sub-studies met inclusion criteria but already included in search, or sub-studies did not meet inclusion criteria |
| Miller J, Knott V. Community-based participatory research in cancer control studies among Indigenous people of Australia, New Zealand, Canada and the US. Asia Pac J Clin Oncol. 2010;6:219. | Sub-studies met inclusion criteria but already included in search, or sub-studies did not meet inclusion criteria |
| Miller J, Knott V, Wilson C, Cunningham J, Condon J et al. Aboriginal and Torres Strait islander cancer control: Review of literature and programs. Asia Pac J Clin Oncol. 2010;6:215. | Sub-studies met inclusion criteria but already included in search, or sub-studies did not meet inclusion criteria |
| Ong KS, Carter R, Vos T, Kelaher M, Anderson I. Cost-effectiveness of interventions to prevent cardiovascular disease in Australia's Indigenous population. Heart Lung Circ. 2014;23(5):414-421. | Sub-studies met inclusion criteria but already included in search, or sub-studies did not meet inclusion criteria |
| Patel J, Durey A, Hearn L, Slack-Smith LM. Oral health interventions in Australian Aboriginal communities: a review of the literature. Aust Dent J. 2017;62(3):283-294. | Sub-studies met inclusion criteria but already included in search, or sub-studies did not meet inclusion criteria |
| Ridani R, Shand FL, Christensen H, McKay K, Tigher J et al. Suicide Prevention in Australian Aboriginal Communities: A review of past and present programs. Suicide Life Threat Behav. 2015;45(1):111-140. | Sub-studies met inclusion criteria but already included in search, or sub-studies did not meet inclusion criteria |
| Dudgeon P, Cox K, D’Anna D, Dunkley C, Hams K et al. Hear our voices: community consultations for the development of an empowerment, healing and leadership program for Aboriginal people living in the Kimberley, Western Australia: final research report. Telethon Institute for Child Health Research. 2012. https://www.telethonkids.org.au/globalassets/media/documents/aboriginal-health/hear_our_voices_final_report.pdf. Accessed 17 Dec 2018. | Sub-studies met inclusion criteria but already included in search, or sub-studies did not meet inclusion criteria |
| Freemantle J, Officer K, McAullay D. Indigenous health - within an international context. Cooperative Research Centre for Aboriginal Health. 2007. https://www.lowitja.org.au/sites/default/files/docs/AustIndigneousHealthReport.pdf. Accessed 12 Dec 2018. | Sub-studies met inclusion criteria but already included in search, or sub-studies did not meet inclusion criteria |
| Villarosa AC, Villarosa AR, Salamonson Y, Ramjan LM, Sousa MS et al. The role of indigenous health workers in promoting oral health during pregnancy: a scoping review. BMC Public Health. 2018;18:381. | Sub-studies met inclusion criteria but already included in search, or sub-studies did not meet inclusion criteria |
