# Supplementary material for: Geographical analysis of evaluated chronic disease programs for Aboriginal and Torres Strait Islander people in the Australian primary health care setting: a systematic scoping review
Source: BMC Public Health. 2019 Aug 14;19:1115. doi: 10.1186/s12889-019-7463-0 (PMC6694647; doi:10.1186/s12889-019-7463-0)
Supplement: Supplementary file 4 — Data extraction: evidence of partnerships and reference to ethical guidelines. This file contains a table of data extracted in relation to the secondary objectives of this review; scoping evidence of partnerships with Indigenous organizations and ethical approaches to undertaking a program evaluation. (DOCX 95 kb) [file 12889_2019_7463_MOESM4_ESM.docx]

**Additional File 4. Data extraction: evidence of partnerships and reference to ethical guidelines**

| **Program name** | **Citation** | **Involvement of Aboriginal Community-Controlled Health Organisation(s) (ACCHOs) (Y/N)** | **Involvement of other Indigenous organisations (Y/N)** | **Reference to National Health and Medical Research Council (NHMRC) ethical guideline (Y/N)*** | **Statement of Human Research Ethics Committee (HREC) review (Y/N)** | **Reference to other ethical protocols or community ethical review (Y/N)** |
| --- | --- | --- | --- | --- | --- | --- |
| Cooking classes for Diabetes Program | Aboriginal Health & Medical Research Council 2009(17)  Abbott, Davison, Moore & Rubinstein 2010(18)  Abbott, Davison, Moore & Rubinstein 2012(19) | Y  Y  Y | N  N  N | N  N  N | N  Y  Y | N  N  N |
| Health Lifestyle and Weight Management Program | Aboriginal Health & Medical Research Council 2009(17) | Y | N | N | N | N |
| Healthy Food Awareness Program | Aboriginal Health & Medical Research Council 2009(17) | Y | N | N | N | N |
| ‘No More Dhonga’ Short Course | Adams et al. 2006(20) | Y | N | N | N | N |
| Home-Based, Outreach case Management of chronic disease Exploratory (HOME) Study program | Askew et al. 2016(21) | Y | Y | Y | Y | Y |
| Renal Treatment Program | Bailie et al. 2006(22) | N | Y | N | Y | N |
| Moorditj Djena program | Ballestas et al. 2014(23) | Y | N | N | N | N |
| Nurse-led practitioner project for chronic kidney disease | Barrett et al. 2015(24) | Y | N | N | N | N |
| Flinders self-management model (CCSM) | Battersby et al. 2008(25) | Y | N | N | Y | N |
| Polycystic Ovarian Syndrome clinic program | Boyle et al. 2017(26) | N | Y | N | Y | N |
| Diabetic retinopathy screening program | Brazionis et al. 2018(27) | Y | Y | N | Y | N |
| Primary Health Care Outreach program of Aboriginal Health Checks | Burgess et al. 2011(28) | N | Y | N | Y | Y |
| 12 week exercise and nutrition program | Canuto 2013(29)  Canuto et al. 2012(30)  Canuto et al. 2013(29) | Y  Y  Y | Y  Y  Y | Y  N  N | Y  Y  Y | Y  Y  Y |
| Healthy Lifestyle Programme (HELP) | Chan et al. 2007(31) | N | N | N | Y | N |
| Cardiac failure education program | Clark et al. 2014(32)  Clark et al. 2015(33) | Y  Y | Y  Y | N  N | N  Y | N  Y |
| Drug and alcohol screening intervention | Clifford et al. 2013(34) | Y | N | N | Y | N |
| Health literacy intervention | Crengle et al. 2017(35) | Y | Y | N | Y | N |
| Grog mob | D'Abbs et al. 2013(36) | Y | N | N | Y | N |
| Cardiac and pulmonary secondary prevention program | Davey et al. 2014(37) | Y | N | N | Y | N |
| Smoking cessation program | DiGiacomo et al. 2007(38) | Y | N | N | N | N |
| Heart health program cardiac secondary prevention | Dimer et al. 2010(39)  Dimer et al. 2012(40)  Dimer et al. 2013(41)  Maiorana et al. 2012(42)  Maiorana et al. 2015(43) | Y  Y  Y  Y  Y | N  N  N  N  N | N  N  N  N  N | N  N  Y  N  N | N  N  N  N  N |
| Intensive quit smoking intervention | Eades et al. 2012(44) | Y | Y | N | Y | N |
| Give up the smokes program | Gould, McGechan & Zwan 2010(45) | Y | N | N | N | N |
| Diabetes Management and Care program | Gracey et al. 2006(46) | N | Y | N | N | N |
| Koorie Men's health day | Isaacs & Lampitt 2014(47) | Y | N | N | Y | Y |
| Oral health literacy program | Ju et al. 2017(48) | Y | N | N | Y | Y |
| Oral health periodontal program | Kapellas et al. 2013(49)  Kapellas et al. 2014a(50)  Kapellas et al. 2014b(51)  Kapellas et al. 2017(52) | Y  Y  Y  Y | N  N  N  N | N  N  N  N | Y  N  Y  Y | N  N  N  N |
| Structured chronic disease care planning program | Kowanko et al. 2012(53) | Y | Y | Y | Y | Y |
| Nurse-led Chronic Kidney Disease program | Lawton et al. 2016(54) | Y | N | N | N | N |
| Walk about Together Program (WAT) | Longstreet et al. 2008(55) | Y | N | N | Y | N |
| Be Our Ally Beat Smoking (BOABS) program | Marley et al. 2014a(56)  Marley et al. 2014b(10) | Y  Y | Y  Y | Y  Y | Y  Y | Y  Y |
| Getting better at chronic care program | McDermott et al. 2015(57)  Schmidt, Campbell & McDermott 2016(58)  Segal et al. 2016(59) | Y  Y  Y | Y  Y  Y | N  N  N | Y  Y  Y | Y  Y  N |
| Work it out program | Mills et al. 2017(60) | Y | N | Y | Y | Y |
| Mental illness brief intervention program | Nagel & Thompson 2008(61)  Nagel et al. 2008(62) | N  N | N  N | N  N | N  Y | N  N |
| Get Healthy Service program | Quinn et al. 2017(63) | N | N | N | Y | N |
| Antiviral therapy Hepatitis C program | Read et al. 2017(64) | N | N | N | Y | N |
| QAAMS program | Shephard 2006(65)  Shephard et al. 2017(66)  Spaeth, Shephard & Schatz 2014(67) | Y  Y  Y | N  N  N | N  N  N | N  N  Y | N  N  N |
| Point-of-Care in Aboriginal Hands | Shepherd et al. 2006(68) | Y | N | N | Y | Y |
| Western Desert Kidney Health Screening program | Sinclair et al. 2016(69) | Y | N | N | Y | N |
| COACH programme | Ski et al. 2017(70) | N | N | N | N | N |
| Diabetic retinopathy screening program | Spurling et al. 2010(71) | Y | N | N | Y | Y |
| Indigenous adult health checks program | Spurling, Hayman & Cooney 2009(72) | Y | N | N | Y | Y |
| Shared medical appointment program | Stevens et al. 2016(73) | Y | Y | N | Y | N |
| Community singing program | Sun & Buys 2012(74)  Sun & Buys 2013a(75)  Sun & Buys 2013b(76)  Sun & Buys 2013c(77)  Sun & Buys 2013d(78)  Sun & Buys 2013e(79)  Sun & Buys 2013f(80)  Sun & Buys 2016(81) | Y  Y  Y  Y  Y  Y  Y  Y | N  N  N  N  N  N  N  N | N  N  N  N  N  N  N  N | Y  Y  Y  Y  Y  Y  Y  Y | N  N  N  N  N  N  N  Y |
| Home Medicines Review program | Swain 2016(82)  Swain & Barclay 2015(83) | Y  Y | N  N | N  N | Y  Y | Y  Y |
| 'Yaka Narali' Tackling Indigenous Smoking program | Tane et al. 2016(84) | Y | N | N | N | Y |
| Ngangkari Program | Togni 2017(85) | N | Y | Y | N | Y |
| Deadly Liver Mob program | Treloar et al. 2018(86) | Y | N | N | Y | Y |
| Music therapy program | Truasheim 2014(87) | Y | N | N | N | N |
| Perinatal mental health program | Verrier et al. 2013(88) | N | Y | N | N | N |

* National Health and Medical Research Council (NHMRC). Values and Ethics: Guidelines for Ethical Conduct in Aboriginal and Torres Strait Islander Health Research [Internet]. 2003 [cited 2017 Nov 5]. Available from: <https://www.nhmrc.gov.au/_files_nhmrc/publications/attachments/e52.pdf>.

It is acknowledged since the date of extracting citations for the review, that the above guideline has been updated to the following two guidelines:

National Health and Medical Research Council (NHMRC). Ethical conduct in research with Aboriginal and Torres Strait Islander Peoples and communities [Internet]. 2018 [cited 2019 Jan 8]. Available from: <https://nhmrc.gov.au/about-us/publications/ethical-conduct-research-aboriginal-and-torres-strait-islander-peoples-and-communities>

National Health and Medical Research Council (NHMRC). Keeping research on track II [Internet]. 2018 [cited 2019 Jan 8]. Available from: <https://nhmrc.gov.au/about-us/publications/keeping-research-track-ii>
